# Iron Salts, but Not Iron Oxide Nanoparticles, Drive High‐Yield Production of Iron‐Engineered Extracellular Vesicles

**DOI:** 10.1002/jev2.70346

**Published:** 2026-08-09

**Authors:** Elliot Thouvenot, Aurore Van de Walle, Jean‐Michel Guigner, Ali Abou‐Hassan, Lorena Martin‐Jaular, Amanda K. A. Silva, Claire Wilhelm

**Affiliations:** ^1^ Laboratoire Physique des Cellules et Cancer Institut Curie, CNRS, Université PSL Paris France; ^2^ Institut de Minéralogie, de Physique des Matériaux et de Cosmochimie IMPMC MNHN CNRS Sorbonne Université Paris France; ^3^ Physicochimie des Électrolytes et Nanosystèmes InterfaciauX (PHENIX) CNRS Sorbonne Université Paris France; ^4^ Institut Universitaire de France (IUF) Paris France; ^5^ INSERM U932 Institut Curie Research Center PSL Research University Paris France; ^6^ CurieCoreTech ExtracellularVesicles Institut Curie Research Center Paris France; ^7^ Laboratoire Matière et Systèmes Complexes, MSC, CNRS UMR7057 Université Paris Cité Paris France

**Keywords:** EDXS cryo‐TEM, ferritin, hydrodynamic bioreactor, iron engineering, magnetic nanoparticles, mesenchymal stem cells

## Abstract

Efficient production of iron‐engineered extracellular vesicles (EVs) is of interest for their use as delivery systems in biotherapy. In this study, we investigated the role of iron in EV biogenesis and evaluated a scalable fluidic strategy for their mass production using turbulence stimulation in bioreactors. Murine mesenchymal stem cells were loaded with iron via ferric quinate or iron oxide nanoparticles (IONPs) and subsequently subjected to turbulence stimulation or serum starvation to induce EV secretion. Using elemental spectroscopy and cryogenic electron microscopy, we found that prolonged incubation with ferric quinate combined with turbulence stimulation resulted in the highest EV yield—60,000 EVs per cell in 3 h—and enabled efficient iron transfer from cells to EVs. In contrast, IONP treatment did not increase EV yield compared to control and showed high variability in iron transfer, as probed by EV magnetophoretic mobility. TEM and XEDS analyses confirmed ferritin‐bound iron encapsulation within EVs, whereas IONPs were poorly internalised. These results highlight the promising role of ionic iron in EV formation and content, demonstrating how iron source, concentration, and incubation duration influence both EV biogenesis and iron engineering.

## Graphical abstract

Soluble iron, but not iron oxide nanoparticles, is efficiently routed into extracellular vesicles (EVs) through physiological cargo‐sorting pathways. Prolonged ferric salt incubation combined with turbulence‐based hydrodynamic stimulation drives massive EV secretion and near‐complete intracellular iron transfer via ferritin. Imaging and elemental analyses reveal selective ferritin loading within EVs, while nanoparticles remain excluded from vesicle lumens. These findings uncover a central role of iron in EV biogenesis and establish a scalable strategy for producing iron‐engineered EVs.

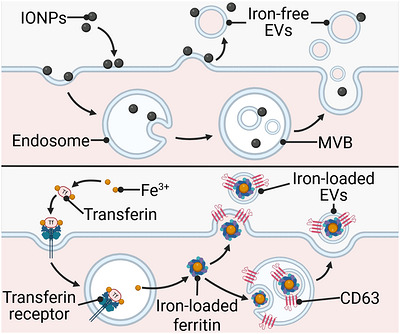

## 1 Introduction

Extracellular vesicles (EVs) are membrane‐delimited entities that play a pivotal role in far‐reaching intercellular communication, influencing many physiological and pathological processes. These vesicles, which include exosomes, ectosomes and apoptotic bodies from 50 to 5000 nm in size, are endogenously secreted by cells either constitutively or in response to stimuli (Hendrix et al. 2023; Couch et al. 2021; Yáñez‐Mó et al. 2015; Mathieu et al. 2019; Manno et al. 2025). Due to their ubiquitous presence, bioactive cargo (proteins, lipids, mRNAs and miRNAs) and multifaceted roles, EVs are increasingly recognised as major players in human healthcare.

Recent advances in EV research unveiled several promising opportunities in medicine at the forefront of emergent biotherapies with technologies poised to transition from bench to bedside. Given their ability to recapitulate a substantial part of the producer cell's biological effects, EVs are regarded as promising candidates for cell‐free biotherapies. This potential represents a significant paradigm shift in regenerative medicine. Previous studies showed the beneficial effect of mesenchymal stem cell (MSC) EVs in the therapy of brain, heart, liver, kidney and skin injuries (Arasu et al. 2017; Bruno et al. 2012; Lai et al. 2010; Azparren‐Angulo et al. 2021; Zhang et al. 2015; Kim et al. 2025).

EVs have the plasticity to be engineered and loaded with endogenous or exogenous therapeutic molecules, positioning them as an eligible bio‐mimetic delivery system. As genuine physiological carriers, EVs are potentially less immunogenic than artificial delivery vehicles. They may advantageously change drug pharmacokinetics, biodistribution an bioavailability by (i) protecting drugs, (ii) addressing them to the site of interest and (iii) facilitating membrane transport through biological barriers (Tatischeff and Alfsen 2011; de Abreu et al. 2021; Kang et al. 2021).

Among the promising classes of bioengineered EVs are those loaded with iron‐based material. First, because iron participates in several vital biological processes, including cellular respiration in mitochondria, cell signalling and host defence, besides oxygen transport in haemoglobin (Vogt et al. 2021). Therefore, preventing iron deficiency is a critical balance reached by a fine‐tuned interplay of uptake, storage and export (Galy et al. 2024). Secondly, because magnetic iron material, such as superparamagnetic iron oxide nanoparticles (IONP) are known to be promising therapeutic agents, being used for instance as imaging contrast agents for MRI, (Wang et al. 2025). allowing photothermal therapies in cancer (Espinosa et al. 2018; Beola et al. 2018) or magnetic targeting (Stanicki et al. 2022), and also being explored for their capacity to induce, after biodegradation, ferroptosis, a form of cellular death triggered by an excess of intracellular iron (Sant'Angelo et al. 2025; Fromain et al. 2023). And lastly, there is a growing body of evidence suggesting a strong interplay between iron and EVs. For instance, CD63, which is known to be intrinsically linked to EV secretion, has recently been shown to be regulated by intracellular iron levels via the iron‐responsive element‐iron regulatory protein (IRE‐IRP) system (Yanatori et al. 2021).

Traditional methods for engineering EVs often involve post‐secretion modifications, either via the functionalization of the EV membrane, which do not allow drug encapsulation and thus protection, or via forced incorporation strategies through membrane disruption, such as electroporation or use of ultrasounds, which can compromise EV integrity (Danilushkina et al. 2023). A gentler and potentially more efficient strategy, suitable for iron engineering, is to take advantage of the fine interplay between intracellular iron and EV secretion by preloading the parental cells with iron oxide nanoparticles and relying on natural cargo sorting mechanisms (Danilushkina et al. 2023). Already applied to IONP engineering, this indirect loading approach allowed to avoid post‐secretion modification but typically yielded low amounts of iron per vesicle, in addition of suffering from limited secretion efficiency (Silva et al. 2012; Andriola Silva et al. 2013; Silva et al. 2013; 2015).

An alternative approach would be to exploit forms of iron that can be incorporated into physiological storage pathways, in particular ferritin. Recent studies have indeed suggested that ferritin itself can be secreted into EVs, thereby offering a bio‐inspired route for iron loading (Truman‐Rosentsvit et al. 2018). Yet, despite this promising concept, ferritin‐based EV engineering remains constrained by the inherently low yields of conventional EV production methods. Therefore, there is a strong need for approaches enabling both highly efficient EV secretion and robust iron loading, while remaining standardised and scalable (Grangier et al. 2021).

Herein, we investigate a fluidic strategy for mass production of iron‐engineered EVs relying on standardised, scalable and high‐yield turbulence stimulation. This method can be implemented in bioreactors in which cells are cultured at a high density at the surface of microbeads stirred by the flow (Berger et al. 2021), and where the turbulent flow created can enhance EV release. In the search for an optimised technological bioprocess for producing iron‐engineered EVs, we therefore compared different strategies: internalization of iron salt or IONPs inside cells, followed by EV production using either serum starvation or turbulence stimulation. This comparison also aims to provide deeper insights into iron trafficking, shedding light on cell‐to‐EV exchange and how it is affected by the iron form and stimulation method to release EVs.

## 2 Material and Methods

### 2.1 Citrate‐Coated IONPs

Nanoparticles were synthesised as first described by Massart and later modified by us (Cabana et al. 2020). In brief, the pH of a mixture of 12 molar ratio of ferrous chloride (FeCl_2_•4H_2_O, 98%, Alfa Aesar) and ferric chloride (FeCl_3_•6H_2_O, >97%, Alfa Aesar) aqueous solutions, respectively, was increased to 12 by the addition of 1 M aqueous NaOH solution (Alfa Aesar) to induce the precipitation of magnetite. After magnetite decantation, the precipitate was washed with ethanol (96%, Sigma–Aldrich), ethyl acetate (>99.5%, Sigma–Aldrich), diethyl ether (100%, Sigma–Aldrich) and acetone (technical grade, Sigma–Aldrich) (three times each). The cleaned nanoparticles were finally oxidised by addition of a boiling aqueous solution of ferric nitrate (Fe(NO_3_)_3_•9H_2_O, >98%, Alfa Aesar) (1 M, 30 min). Afterwards, the nanoparticles were decanted, washed with nitric acid (HNO_3_, 70%, VWR) and later with the different solvents as previously. The obtained maghemite was coated with trisodium citrate (VWR) and resuspended in water. Citrate bound to the iron oxide surface, providing a negative charge through carboxylate groups (COO–) that stabilised the nanoparticles in water through electrostatic repulsion.

### 2.2 2D Cell Culture

mMSCs (Murine C3H/10T1/2, Clone 8, ATCC) were cultured at 37°C in a 5% CO_2_ atmosphere using T150 culture flask (TPP, 90150) and DMEM (Gibco, 61965‐026) supplemented with 10% heat‐inactivated FBS (Dutscher, S1900‐500C) and 1% penicillin and streptomycin (Gibco, 15140‐122). NIH/3T3 fibroblasts (ATCC, CRL‐1658) were cultured at 37°C in a 5% CO_2_ atmosphere using a T25 culture flask (TPP, 90026) and DMEM (Gibco, 61965‐026) supplemented with 10% calf serum (ATCC‐30‐2030) and 1 µg/mL puromycin (Gibco, A1113802).

### 2.3 3D Cell Culture

A total of 50 mL of a 10 g/L suspension of dextran microbeads (Cytiva, Cytodex 1, 17044801) in PBS (Sigma, D8537), corresponding to 2 × 10^6^ microbeads, was sterilised by autoclave and added to a 500 mL stirred tank (Chemglass, CLS‐1430‐500). The suspension was completed with 200 mL of either DMEM (Gibco, 61965‐026, control or ferric quinate conditions) or RPMI 1640 (Gibco, 31870‐025, IONP conditions) and incubated 1 h at 37°C for equilibration. After medium removal, microbeads were resuspended in 250 mL of the respective medium supplemented with 1% penicillin and streptomycin (Gibco, 15140‐122) and incubated for another hour. mMSCs (passage 5–20) were detached using TrypLE Express (Gibco, 12605‐010), and approximately 20 × 10^6^ cells were added to the microbead suspension. The bioreactor was incubated at 37°C in a 5% CO_2_ atmosphere and agitated at 30 RPM for 3 min every 45 min for 18 h on a magnetic stirrer (Wheaton, W900701‐C) to allow mMSC seeding on microbeads. After 1 h, 10% FBS (Dutscher, S1900‐500C) was added. Following the seeding step, the suspension was continuously agitated at 30 RPM for 24 h to amplify the cells on the microbeads.

### 2.4 Cell Counting on Microbeads

After amplification, 1 mL of microbead‐seeded mMSCs was collected, washed three times in PBS (Sigma, D8537), resuspended in 1 mL of PBS, and stained with 1 µL of a 1000× DAPI solution (Invitrogen, D5371). The suspension was agitated in the dark for 45 min at room temperature, washed three times in PBS and imaged under a Nikon Eclipse Ti inverted microscope in both bright field and fluorescence mode (excitation: 350 nm; emission: 460 nm). The number of nuclei per bead was counted to determine the cell‐seeding density.

### 2.5 Early‐Stage IONP Incubation

After amplification, microbead‐seeded mMSCs were washed three times in PBS (Sigma, D8537), resuspended in 50 mL of 220 nm filtered RPMI 1640 (Gibco, 32404‐014), and incubated with a final concentration of 2 mM IONPs and 1 mM citrate (Sigma, 71406) for 30 min at 4°C with regular agitation. After incubation, the suspension was washed three times in 220 nm filtered RPMI 1640 and resuspended in 350 mL of 220 nm filtered RPMI 1640 supplemented with 1% penicillin and streptomycin (Gibco, 15140‐122).

### 2.6 Late‐Stage IONP Incubation

When mMSCs reached ∼80% confluency, they were washed three times with 220 nm filtered RPMI 1640 (Gibco, 32404‐014) and incubated with a 1 mM citrate (Sigma, 71406) and 2 mM IONPs in 220 nm filtered RPMI 1640 for 30 min at room temperature. Cells were then washed three times and incubated overnight in 220 nm filtered RPMI 1640 supplemented with 10% FBS (Dutscher, S1900‐500C) and 1% penicillin and streptomycin (Gibco, 15140‐122).

### 2.7 Iron Salt (Ferric Quinate) Preparation and Incubation

A 100 mM ferric quinate solution was freshly prepared by dissolving 27 mg of ferric chloride (Sigma, 236489) and 19 mg of quinic acid (Sigma, 138622) in 1 mL of 220 nm filtered distilled water. After complete dissolution, the solution was filtered at 220 nm to remove any undissolved salts and to ensure sterilization. Once mMSCs reached ∼80% confluency, they were washed three times with PBS (Sigma, D8537) and incubated for 1–14 days with 0.25–4 mM ferric quinate solution in DMEM (Gibco, 31053‐028) supplemented with 10% FBS (Dutscher, S1900‐500C), 1% penicillin and streptomycin (Gibco, 15140‐122), and 1 mM citrate (Sigma, 71406). The culture medium was replaced with fresh ferric quinate‐supplemented medium every 3 days.

### 2.8 EV Production Through Turbulence Stimulation

Post‐amplification (control, late‐stage IONP and ferric quinate conditions) or post‐incubation (early‐stage IONP condition), microbead‐seeded mMSCs were washed three times with either 220 nm filtered DMEM (Gibco, 31053‐028, control and ferric quinate conditions) or RPMI 1640 (Gibco, 32404‐014, IONP conditions). They were then resuspended in 350 mL of the respective medium, supplemented with 1% penicillin and streptomycin (Gibco, 15140‐122). The bioreactor was incubated at 37°C in a 5% CO_2_ atmosphere and agitated at 200 RPM for 3 h. The conditioned medium was then separated from the microbead‐seeded mMSCs, centrifuged at 2000 RPM to remove cell de‐bris and stored at 4°C for further use.

### 2.9 EV Production Through Serum Starvation

Upon reaching ∼80% confluency (control condition) or after iron incubation (late‐stage IONP and ferric quinate conditions), cells were washed three times with either 220 nm filtered PBS (Sigma, D8537, control and ferric quinate conditions) or 220 nm filtered RPMI 1640 without phenol red (Gibco, 32404‐014, late‐stage IONP condition). The medium was then replaced with 20 mL of either 220 nm filtered DMEM (Gibco, 31053‐028, control and ferric quinate conditions) or 220 nm filtered RPMI 1640 (late‐stage IONP condition) supplemented with 1% penicillin and streptomycin (Gibco, 15140‐122) and incubated for 72 h in a 37°C and 5% CO_2_ environment. After incubation, the conditioned medium was collected, centrifuged at 2000 RPM to remove cell debris, and stored at 4°C for further analysis.

### 2.10 EV Concentration and Purification

Previously collected conditioned media were concentrated by filtration into 0.5 mL of concentrated conditioned medium (CCM) using 100 kDa cut‐off centrifugal filters (Sigma, Centricon Plus‐70 100 kDa, UFC710008) following the supplier's instructions. CCM were subsequently purified by size exclusion chromatography. Briefly, SEC columns (Izon Sciences, qEVoriginal/70 nm Gen2 SEC columns, ICO‐70) were rinsed with 25.5 mL of either 220 nm filtered DMEM (Gibco, 31053‐028, control and ferric quinate conditions) or 220 nm filtered RPMI 1640 (Gibco, 32404‐014, IONP conditions) and 0.5 mL of CCM were added to the top of the columns, followed by either DMEM or RPMI 1640, depending on the condition. The first 2 mL fraction was discarded, and the following 2.4 mL fraction (EV fraction) was collected. EV fractions were then reconcentrated by filtration into 50–100 µL of concentrated EV suspension (EV pool) using 10 kDa cut‐off centrifugal filters (Millipore, Amicon Ultra‐4, UFC8010). EV pools were then stored at 4°C for further analysis or preserved at –80°C for long‐term storage.

### 2.11 Inductively Coupled Plasma—Optical Emission Spectroscopy (ICP‐OES) Analysis

Between 1.5 × 10^5^ and 3 × 10^5^ cells per sample, or 1 × 10^9^ EVs per sample were digested in 290 µL of 69% nitric acid (Sigma). Upon minimum 2 days of digestion at room temperature, the samples were diluted in filtered ultrapure water to obtain a final concentration of 2% in nitric acid. The total iron content of the samples was subsequently measured by inductively coupled plasma—optical emission spectroscopy (ICP‐OES, SpectroGreen, SPECTRO). Nebulizer, auxiliary gas and coolant flowrates were set at 800, 900 and 8000 mL/min, respectively. Plasma power was set at 1300 W.

### 2.12 TEM

For electron microscopy cellular observations, cells were rinsed and fixed with 5% glutaraldehyde in 0.1 mol/L sodium cacodylate buffer and postfixed with 1% osmium tetroxide solution containing 1.5% potassium cyanoferrate. Samples were then gradually dehydrated in increasing concentrations of ethanol and embedded in Epon resin. Ultrathin 70 nm sections were deposited on 200 mesh copper grids, counterstained with lead citrate before being examined with a Hitachi HT 7700 TEM operated at 80 kV (Elexience, INRAE, GABI, France).

### 2.13 Perls Prussian Blue Iron Colouration

Nuclear Fast Red solution was prepared by adding 0.25 w/w% of Nuclear Fast Red (Sigma, 60700) and 5 w/w% of aluminium sulphate hydrate (Sigma, 368458) in distilled water. The solution was boiled while being agitated until complete dissolution, then filtered at 450 nm. Potassium ferrocyanide solution was freshly prepared by dissolving 5 w/w% of potassium hexacyanoferrate(II) trihydrate (Sigma, P3289) in distilled water and mixing it with 1 M hydrochloric acid (Fisher Chemical, J/4320/15) in a 1:1 volume ratio. mMSCs were cultured as previously described on FBS‐treated glass coverslips in 24‐well plates (TPP, 92024) until reaching ∼80% confluency, followed by iron incubation under previously described conditions. After incubation, mMSCs were washed once with either PBS (Sigma, D8537, control and quinate conditions) or RPMI 1640 (Gibco, 32404‐014, IONP conditions), and fixed with 4% paraformaldehyde (Electron Microscopy Sciences, 15710) for 1 h at room temperature. The cells were then washed three times with distilled water. 500 µL of potassium ferrocyanide solution was added to each well and the plate was agitated at room temperature for 10 min. Following two washes with distilled water, wells were incubated with 500 µL of Nuclear Fast Red solution and agitated at room temperature for 15 min. Cells were then dehydrated through successive 5‐min washes with 70%, 95% and 100% ethanol, followed by three 5‐min incubation with HistoChoice clearing agent (Sigma, H2779). Finally, the stained coverslips were mounted onto glass slides using Eukitt quick‐hardening mounting medium (Sigma, 03989) and imaged in bright‐field mode using a Nikon Eclipse Ti inverted microscope.

### 2.14 Nanoparticle Tracking Analysis (NTA)

EV samples were centrifuged at 2000 *g* and 4°C for 10 min to remove debris before being diluted between approximately 2 × 10^7^ and 2 × 10^8^ particles/mL. NTA was performed on the diluted samples using the NanoSight NS300 (Malvern) along with the NTA 3.4 software. During analysis, the camera level was set at 16, the flow rate was adjusted within the range of 20–30 µL/min and the detection threshold was set at 4. It should be noted that NTA has a lower detection limit of approximately 60 nm, and should therefore not be sensitive to nanoparticles in the 8–10 nm size range. Control experiments confirming the lack of detection of iron oxide nanoparticles under these conditions are shown in Figure S1.

### 2.15 Proliferation Assay

mMSCs were seeded at a density of 80,000 cells/cm^2^ into each well of a 24‐well plate (TPP, 92024). Following initial cell attachment, the monolayers were incubated with either IONPs or ferric quinate as described above. For the IONP condition, cells were incubated for 30 min, washed thoroughly with RPMI 1640 medium, and subsequently maintained in complete RPMI 1640 medium for durations ranging from 3 to 14 days. For the ferric quinate condition, continuous incubation was sustained for durations ranging from 1 to 14 days. At designated time points, the cells were detached using a dissociation reagent (TrypLE Express, Gibco, 12605‐010) and quantified using an automated cell counter (Countess 3 FL, Invitrogen). The proliferation index was calculated as the ratio of the final cell count at harvesting to the initial number of seeded cells.

### 2.16 Radioimmunoprecipitation Assay (RIPA) Lysis

EV samples or mMSCs were lysed using RIPA buffer (Thermo Fisher Scientific, 89901) containing 20 mM Tris.HCl pH 7.6, 150 mM NaCL, 1% NP‐40, 1% sodium deoxycholate and 0.1% SDS. A total of 2.5 µL of EV sample were mixed with 15 µL of RIPA and incubated for 30 min in ice, then sonicated for 30 s with 50% pulse. EV lysates were then diluted by adding 132.5 µL of PBS to achieve a dilution factor of 1:10 in RIPA. A total of 106 mMSCs were washed two times in PBS, then centrifuged at 2500 *g* for 5 min. Cell pellet was then resuspended in 200 µL of RIPA buffer, sonicated for 30 s with 50% pulse and incubated in ice for 30 min. Cell lysate was the centrifuged at 14,000 *g* to remove cell debris, and the supernatant was put in a 0.5 mL tube (Dutscher, 33283) to further protein quantification.

### 2.17 Protein Quantification Assay

Protein quantification of EVs or cell lysates was performed using the Micro BCA protein assay kit (Thermo Fisher Scientific, 23235). A standard curve was pre‐pared using Bovine Serum Albumine (BSA), with final concentrations of 200, 40, 20, 10, 5, 2.5, 1, 0.5 and 0 µg/mL. Protein quantification assays were then conducted as indicated in manufacturer's instructions. Briefly, 45 µL of each sample and of each standard were pipetted in triplicates in wells of a 384‐well microplate (PerkinElmer, Optiplate‐384 F HB, Black). A total of 45 µL of the prepared BSA working reagent were then added to each well, and the plate was placed on a plate shaker for 30 s, before being covered and incubated at 37°C for 2 h. Absorbance at 562 nm of each well was then measured using a plate reader (PerkinElmer, Ensight Multimode Plate Reader), and protein concentrations were derived from the standard curve. To exclude potential colorimetric interference caused by iron material, control assays were performed by spiking a 20 µg/mL bovine serum albumin (BSA) standard with varying concentrations of both IONPs and ferric quinate (Figure S2). The maximum variations in absorbance readings introduced by the presence of iron compared to iron‐free BSA controls were 2.4% for IONPs and 7.2% for ferric quinate. These deviations remained well within the typical experimental uncertainty of the microBCA assay, thereby validating the accuracy of the protein measurements for subsequent Western blot normalization.

### 2.18 Western Blotting Analysis

Volumes of purified extracellular vesicles or cell lysate corresponding to 5 µg of protein were resuspended in 4× Laemmli Sample buffer (Bio‐Rad), boiled 10 min at 95°C and loaded on 4%–15% Mini‐Protean TGX Stain‐Free gels (Bio‐Rad), under non‐reducing conditions. Transferred membranes (Immuno‐Blot PVDF Bio‐Rad) were incubated with primary anti‐mouse antibodies: CD9 (Clone MM2/57, Milipore CBL162, 1/1000), CD63 (Clone R5G2, MBL D263‐3, 1/200), CD81 (Clone B‐11, Santa Cruz Biotechnology sc‐166029, 1/1000), Syntenin‐1 (Clone C2C3, GeneTex 108470 1/1000), Ferritin (Clone 8E1E7, Abcam 69090, 1/1000) and 14‐3‐3 (Abcam ab125032, 1/1000). Secondary antibodies included HRP‐conjugated goat anti‐rabbit IgG (H + L) (Jackson 111‐035‐144) and HRP‐conjugated goat anti‐mouse IgG (H + L) (Jackson 111‐035‐146). Membranes were developed using Clarity western ECL substrate (Bio‐Rad) and the ChemiDoc Touch imager (Bio‐Rad). Band intensities were then measured with the FiJi software (version 1.54f) and renormalised by the total protein intensity of the stain‐free gels.

### 2.19 Nanomagnetophoresis Assay

Nanomagnetophoresis assays were performed using a custom‐designed microfluidic chip. The chip was fabricated with a 3D‐printed mould, created with a DigitalWax 028J Plus 3D‐printer and DigitalWax DS3000 resin. The mould featured a rectangular (2.5 × 5 mm) shape with a side hole and two side channels. A 500 µm nickel needle was inserted into the side hole, and unpolymerised polydimethylsiloxane (PDMS), prepared by combining SYLGARD 184 Silicone Elastomer Base and SYLGARD 184 Silicone Elastomer Curing Agent in a mass ratio of 10:1, was poured into the mould. To avoid the formation of air bubbles, the PDMS‐filled moulds were degassed and then incubated at 70°C for 24 h to facilitate PDMS polymerization. After curing, the needle was removed, the PDMS layer was carefully unmoulded, and 1 mm inlets and outlets were punched at the ends of the channels. The PDMS layer and a glass coverslip were cleaned with isopropanol and surface‐activated through plasma treatment using the Diener PICO plasma cleaner. The chip was then immediately assembled by aligning the PDMS layer on the coverslip and reinserting the needle into the side hole. EV samples were labelled with DIL fluorescent dye (Sigma, 42364) by mixing 5 µL of the EV solution with 5 µL of 2× DIL solution in 25 mM HEPES (Sigma, H4034) and incubated overnight at 4°C. The solution was then diluted 5‐fold in 25 mM HEPES before being injected into the magnetophoresis chip. The chip was placed under a Leica DMi8 spinning disk microscope, and a magnet was placed next to the chip, adjacent to the needle. Fluorescent images of the needle vicinity were acquired (excitation: 561 nm; emission: 607 nm) using a 63× objective with exposure times ranging from 30 to 200 ms. EV trajectories were then tracked using the TrackMate 7 plugin in FiJi software (version 1.54f), employing the LoG detector and the simple LAP tracker. Tracks were manually corrected, if necessary, using the TrackScheme tool.

### 2.20 Cryogenic Transmission Electron Microscopy (cryoTEM) and Electron Dispersive X‐Ray Spectroscopy (EDXS) Analysis

A 5 µL drop of the EV sample solution was deposited on quantifoil (Quantifoil Micro Tools GmbH) carbon membrane grids. The excess of liquid on the grid was absorbed with a filter paper and the grid was quench‐frozen quickly in liquid ethane to form a thin vitreous ice film using a homemade mechanical cryo plonger. Once placed in a Gatan 626 cryo‐holder cooled with liquid nitrogen, the samples were transferred in the microscope and observed at low temperature (–180°C). Cryo‐TEM images were recorded on Ultrascan 1000, 2k × 2k pixels CCD camera (Gatan), using a LaB6 JEOL JEM2100 (JEOL, Japan) cryo‐microscope operating at 200 kV with a JEOL low dose system (Minimum Dose System, MDS, JEOL) to protect the thin ice film from any irradiation before imaging and reduce the irradiation during the image capture. Electron Dispersive X‐ray Spectroscopy (EDXS) was conducted with a JEOL EDXS detector with a 140‐eV resolution.

### 2.21 In Vitro Scratch Wound Healing Assay

Murine fibroblasts (NIH/3T3, ATCC CRL‐1658) were seeded into 96‐well plates (TPP, 92096) at a density calibrated to achieve approximately 80% confluency after 24 h of expansion. On Day 0, a standardised linear scratch wound was introduced across the confluent cell monolayer using a sterile 200 µL pipette tip guided by a custom 3D‐printed homemade alignment tool to ensure reproducible scratch geometry. Cellular debris and detached cells were removed by washing the wells once with PBS (Sigma, D8537). The medium was then replaced with 100 µL per well of the respective treatment or control media: serum‐free DMEM (Gibco, 61965‐026), complete DMEM supplemented with 10% heat‐inactivated FBS (Dutscher, S1900‐500C) and 1% penicillin‐streptomycin (Gibco, 15140‐122), serum‐free DMEM supplemented with EVs secreted by untreated mMSCs (Starv. Ctrl, 2 × 10^8^ EVs/mL), or serum‐free DMEM supplemented with EVs secreted by mMSCs pre‐incubated with 1 mM ferric quinate for 14 days (Starv. Salt_14d_, 2 × 10^8^ EVs/mL). Bright‐field images of the wounded areas were captured at 0, 24 and 48 h post‐scratching using an Ensight Multimode Plate Reader (PerkinElmer) operating in bright‐field imaging mode. The cell‐free wound area was quantified at each time point using FiJi software (version 1.54p). The wound closure percentage was calculated by normalising the recovered migration area against the initial scratch area measured at 0 h for each individual well.

## 3 Results

### 3.1 Source and Incubation Parameters Influence Intracellular Iron Load and Distribution

Optimising the production of iron‐loaded EVs begins with efficient labelling of parental cells, specifically by maximising intracellular iron accumulation in a form compatible with secretion, while preserving cellular viability and metabolic function. To achieve this, murine MSCs (mMSCs) were incubated with either superparamagnetic IONPs or ferric quinate (iron salt). The latter was selected for its solubility, stability in culture medium, efficient cellular uptake and compatibility with iron‐related intracellular processes, as well as for its established use as an iron source in magnetosome biosynthesis in magnetotactic bacteria (Frankel et al. 1979; Staniland et al. 2007).

IONP internalization protocols had been established previously and were applied without modification (Silva et al. 2012, 2013, 2015). Briefly, mMSCs were incubated with 2 mM citrate‐coated maghemite (γ‐Fe_2_O_3_) IONPs for 30 min at room temperature in RPMI 1640 medium supplemented with 1 mM citrate, followed by a 2‐h chase. The biocompatibility of this IONP incubation protocol was confirmed by the lack of significant variance in cell proliferation up to 14 days post‐treatment between IONP‐treated and untreated control cells (Figure S3). In contrast, iron salt labelling had not yet been systematically evaluated and was explored by incubating cells with concentrations ranging from 0.25 to 4 mM for durations of 1–14 days.

**FIGURE 1:**
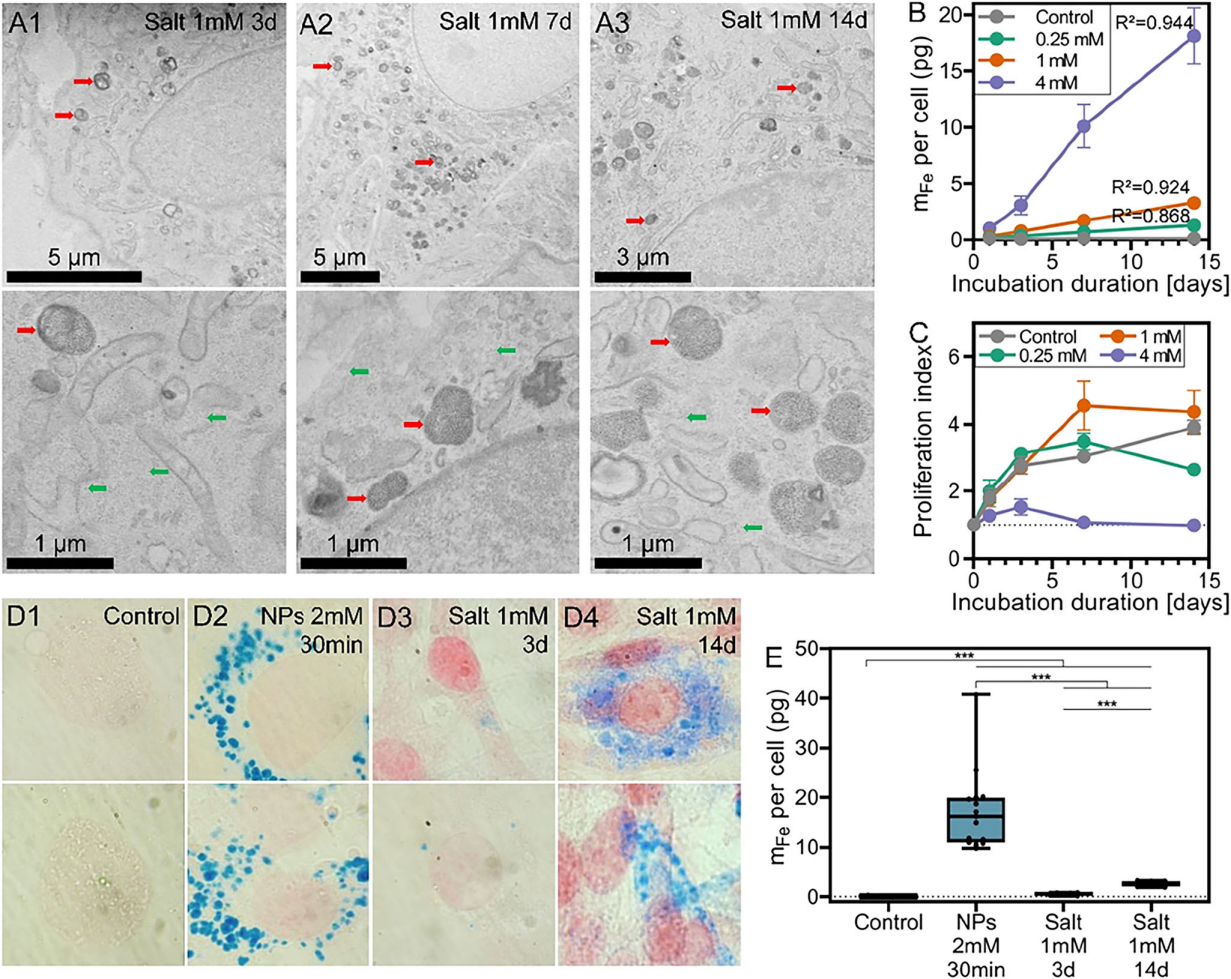
MSC loading with iron material. (A) Transmission electron microscopy of mMSCs incubated with 1 mM ferric salt for 3, 7, or 14 days (A1–A3). (B) Quantification of intracellular iron after exposure to 0–4 mM ferric salt for 1–14 days. Results are shown as mean ± SD. (C) Proliferation index after exposure to 0–4 mM ferric salt for 1–14 days. Results are shown as mean ± SD. (D) Perls’ staining of untreated mMSCs (D1), mMSCs incubated with 2 mM IONPs for 30 min followed by a 2‐h chase (D2), and mMSCs incubated with 1 mM ferric salt for 3 or 14 days (D3–D4). Magnification 375X. (E) Iron content measured in untreated mMSCs, and mMSCs treated with 2 mM IONPs for 30 min followed by a 2‐h chase, or with 1 mM ferric salt for 3 or 14 days. These findings demonstrate that ferric quinate exposure allows a dose‐ and time‐dependent intracellular iron accumulation without altering cell proliferation up to 14 days, providing a viable alternative to nanoparticle loading. Each data point represents an independent experiment. Statistical significance is indicated as *** = *p* < 0.001 (two‐tailed Student's *t*‐test).

Transmission electron microscopy (TEM) of cells incubated with 1 mM iron salt for 3 days (Figure 1A1, Figure S4), 7 days (Figure 1A2, Figure S5) and 14 days (Figure 1A3, Figure S6) revealed distinct electron‐dense particles (∼7 nm), consistent with iron‐loaded ferritin. These structures became more abundant over time and were observed both dispersed in the cytosol (green arrows) and clustered within endosomal compartments (red arrows). Total intracellular iron content was quantified by inductively coupled plasma optical emission spectrometry (ICP‐OES) (Figure 1B). Under control conditions (0 mM iron salt), basal intracellular iron was detected at 0.13 ± 0.02 pg/cell. Under iron supplementation, its accumulation increased in a dose‐ and time‐dependent manner, reaching up to ∼17.5 pg/cell with 4 mM for 14 days. Although this 4 mM condition resulted in the highest intracellular iron content at all‐time points, it also induced cytotoxicity as evidenced by reduced proliferation after just 1 day (Figure 1C). In contrast, 1 mM iron salt did not impair cell proliferation over 14 days and enabled substantial iron accumulation, from 0.34 ± 0.06 pg/cell after 1 day to 3.3 ± 0.5 pg/cell after 14 days. A strong linear correlation (*R* ^2^ > 0.99) between incubation time and intracellular iron content was observed for each concentration, with accumulation rates of 0.084, 0.228 and 1.34 pg/day for 0.25, 1 and 4 mM, respectively.

Based on these results, two conditions were selected for further analysis: 3‐day incubation with 1 mM iron salt (Salt_3d_) and 14‐day incubation with 1 mM iron salt (Salt_14d_), both maintaining cell viability while providing increasing levels of intracellular iron in cytosolic and endosomal compartments. These were compared with control cells (Ctrl) and IONP‐loaded cells (NPs).

Perl's Prussian blue staining and ICP‐OES analysis further characterised the intracellular iron load in each condition (Figure 1D,E). No staining was detected in untreated controls (Figure 1D1), despite a confirmed endogenous iron level of 0.13 ± 0.08 pg/cell measured by ICP. IONP treatment resulted in intense, endosome‐localised staining with consistent distribution among cells (Figure 1D2), and a high but variable iron content (17.2 ± 8.0 pg/cell). Iron salt‐treated cells showed both cytosolic and endosomal staining, increasing in intensity with incubation time (Figure 1D3,D4), although short‐term incubation induced a high cell‐to‐cell variability. These findings were confirmed by ICP, with 0.57 ± 0.29 pg/cell at 3 days and 2.65 ± 0.53 pg/cell at 14 days.

### 3.2 EV Production From Iron‐Loaded MSCs Under Serum Starvation

**FIGURE 2:**
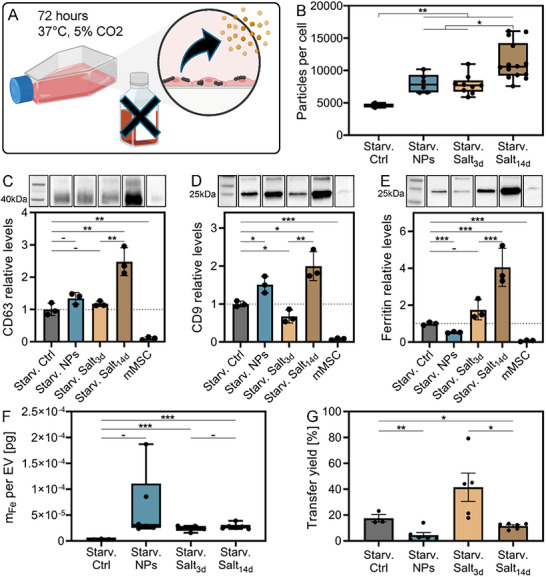
EV production from iron‐loaded MSCs through serum starvation. (A) Overview of the starvation‐based EV secretion protocol. Created with BioRender.com. (B) Particle yield per cell after 72 h of serum starvation from untreated mMSCs (Starv. Ctrl), mMSCs incubated with 2 mM IONPs for 30 min followed by a 2‐h chase (Starv. NPs) and mMSCs incubated with 1 mM ferric salt for 3 or 14 days (Starv. Salt_3d_ and Starv. Salt_14d_). (C–E) Western blot analysis of EV markers CD63 (C), CD9 (D) and ferritin (E) in EVs and parental cells, and corresponding relative levels with Starv. Ctrl as a reference. (F) Iron content per EV under each condition. (G) Iron transfer yield, calculated as the ratio of iron exported in EVs to intracellular iron content. Collectively, these results indicate that intracellular ferritin‐bound iron accumulation correlates with a robust boost in EV secretion and altered protein signatures under standard serum starvation. Each data point represents an independent experiment. Statistical significance is indicated as * = *p* < 0.05, ** = *p* < 0.01 and *** = *p* < 0.001 (two‐tailed Student's *t*‐test).

To evaluate whether the previously optimised iron‐loading protocols support the secretion of iron‐loaded EVs, serum starvation was used as a conventional stimulus for EV release. This widely adopted method in monolayer MSC culture involved placing iron‐preloaded cells into serum‐free medium for a defined duration, in this case 72 h (Figure 2A).

Nanoparticle tracking analysis (NTA), which allows the measurement of cell‐released particles in the nm‐size range, was performed on culture media collected after 72 h (Figure 2B). It revealed that intracellular iron, irrespective of its form, significantly enhanced particle secretion relative to control cells. Control MSCs (Starv. Ctrl) secreted 4617 ± 375 particles per cell, whereas IONP‐treated cells (Starv. NPs) and short‐term iron salt‐incubated cells (Starv. Salt_3d_) secreted 8019 ± 1433 and 7808 ± 1496 particles per cell, respectively. Long‐term incubation with iron salt (Starv. Salt_14d_) further increased secretion to 11,344 ± 2837 particles per cell, corresponding to a 2.5‐fold increase over control. These results indicate a positive correlation between intracellular iron accumulation and EV secretion, particularly with iron salt preconditioning.

Size of released particles was also modulated by iron treatment, with a significant reduction of 11.45–13.65 nm (–10.8% to –12.6%) in mean diameter being observed after long‐term iron salt exposure, compared to other conditions (Figure S7A). However, the coefficient of variation remained similar (∼51%) across all groups (Figure S7B), indicating stable polydispersity regardless of iron status. In addition to size distribution, the surface charge of the particles was evaluated using Tunable Resistive Pulse Sensing (TRPS). The zeta potential profiles remained unchanged between control condition (–10.9 ± 15.2 mV) and long‐term iron salt exposure condition (–10.5 ± 11.9 mV) (Figure S8). This identical charge signature indicates that long‐term iron salt treatment preserves the native electrochemical surface properties of the secreted particles.

Western blot analysis of purified samples confirmed the expression of canonical EV membrane proteins, in accordance with MISEV guidelines (Welsh et al. 2024). Specifically, CD63 (Figure 2C) and CD9 (Figure 2D), recognised EV markers, were robustly detected in the particles secreted across all conditions, with an enrichment of 9.5 ± 4.3‐fold for CD63 and 12.1 ± 4.2‐fold for CD9 in control conditions compared to parental cells. Compared to the control, iron incubations significantly increased CD63 expression. It is specifically marked for the prolonged iron salt incubation (Starv. Salt_14d_) that significantly increased CD63 expression by 2.5 ± 0.4‐fold, in accordance with the observations already described in the literature of a positive correlation between ferritin‐associated iron accumulation within the cells and secretion of CD63+ EVs (Yanatori et al. 2021). CD9 showed more complex regulation, with an increased expression by 1.5 ± 0.2‐fold and 2.0 ± 0.4‐fold following IONP or 14‐day ferric salt incubation, respectively, but reduced expression after short term salt treatment. Surprisingly, neither CD81 nor syntenin‐1, usually considered as canonical EV markers, were detected (Figure S9), suggesting potential subpopulation‐specific release or marker degradation. As expected for high‐purity EV preparations, the non‐vesicular negative marker 14‐3‐3 was undetectable in control vesicles (Starv. Ctrl), and prolonged pre‐incubation with iron salts (Starv. Salt_14d_) did not induce the expression or co‐isolation of this intracellular contaminant (Figure S10), thereby confirming the structural integrity and purity of the recovered vesicular fractions.

Given the accumulation of ferritin‐stored iron in cells treated with iron salts, we investigated ferritin expression in the secreted particles (Figure 2E). Notably, iron loading markedly modulated ferritin presence in the purified particles. While short iron salt incubation (Starv. Salt_3d_) had a small, non‐significant effect on the ferritin levels (1.8 ± 0.5‐fold increase), prolonged incubation induced a strong and significant 4.0 ± 1.0‐fold increase, confirming the direct link between intracellular ferritin‐stored iron accumulation and ferritin secretion within released particles. Interestingly, IONP exposure nearly halved ferritin expression in particles (0.53 ± 0.04‐fold), although the exact mechanism underlying this negative regulation remains to be elucidated. Furthermore, ferritin was significantly enriched in the purified particle samples relative to parental cells by 13.4 ± 7.9‐fold under control conditions, supporting the view of ferritin as a cytosolic EV marker. Therefore, the parallel enrichment of membrane markers CD63 and CD9 and cytosolic ferritin confirms that the secreted particles represent genuine and intact EVs. Moreover, the interplay between ferritin expression and iron exposure suggests that ferritin serves not only as a biomarker for EV identity, but also as a functional cargo reflecting the cell's iron status.

As ferritin alone is not sufficient to attest of an iron cargo within EVs, total vesicular iron content was quantified by inductive coupled plasma mass spectroscopy (ICP‐MS) (Figure 2F). Control EVs contained low but detectable amounts of endogenous iron (3.2 ± 0.7 × 10^−6^ pg/EVs), underscoring that iron secretion within EVs is a natural process even without abnormal intracellular iron accumulation. IONP‐loaded EVs had a higher average iron content (3.9 ± 2.6 × 10^−5^ pg/EV), though large variability across replicates rendered this difference statistically non‐significant. Ferric salt conditions, by contrast, yielded consistent and significant increase in iron per EV: 2.4 ± 0.5 × 10^−5^ pg/EV for the short incubation condition, and 2.8 ± 0.6 × 10^−5^ pg/EV for the long incubation condition. Interestingly, iron loading was found constant for both incubation durations, suggesting a potential saturation effect in EV loading capacity.

To evaluate more precisely the efficiency of iron export via EVs, we derived the iron transfer yield, defined as $\mathrm{Transfer}\;\mathrm{Yield}=\frac{\left(\mathrm{Iron}\;\mathrm{per}\;\mathrm{EV})\times (\mathrm{EVs}\;\mathrm{per}\;\mathrm{cell}\right)}{\mathrm{Iron}\;\mathrm{per}\;\mathrm{cell}}$ (Figure 2G). Under control conditions, 17.5 ± 4.9% of endogenous cellular iron was secreted into EVs, again confirming that EV‐mediated iron release is a natural process. IONP‐treated cells, however, showed drastically reduced transfer efficiency, with 2.6 ± 1.5%, indicating poor packaging of IONP into vesicles. Conversely, ferric salt‐treated cells exhibited better results, the short incubation condition (Starv. Salt_3d_) showing high but highly variable transfer rates (41.5 ± 24.6%), while the prolonged incubation condition (Starv. Salt1_4d_) yielded a lower but more consistent efficiency (11.6 ± 2.2%). This difference in variability is likely due to the iron loading heterogeneity across the cell population for the 3‐day incubation period, as evidenced by Perl's staining (Figure 1D).

Altogether, these results demonstrate that both EV secretion triggered by serum starvation and iron cargo incorporation are enhanced by iron preloading, with large discrepancies depending on the iron source. Ferric salts showed a greater impact on EV secretion, protein signature and iron cargo, highlighting the deep interplay between ferritin‐associated iron accumulation within producer cells and EV release. In contrast, IONPs failed to be efficiently sorted into vesicles, likely due to endosomal sequestration. Since IONP‐engineered EVs have a greater therapeutical interest due to their magnetic and photothermal properties, there remains a need for a more scalable production strategy that could both allow IONP sorting into EVs and that could enhance iron‐loaded EV secretion.

### 3.3 EV Production From Iron‐Loaded MSCs Through Hydrodynamic Turbulence

**FIGURE 3:**
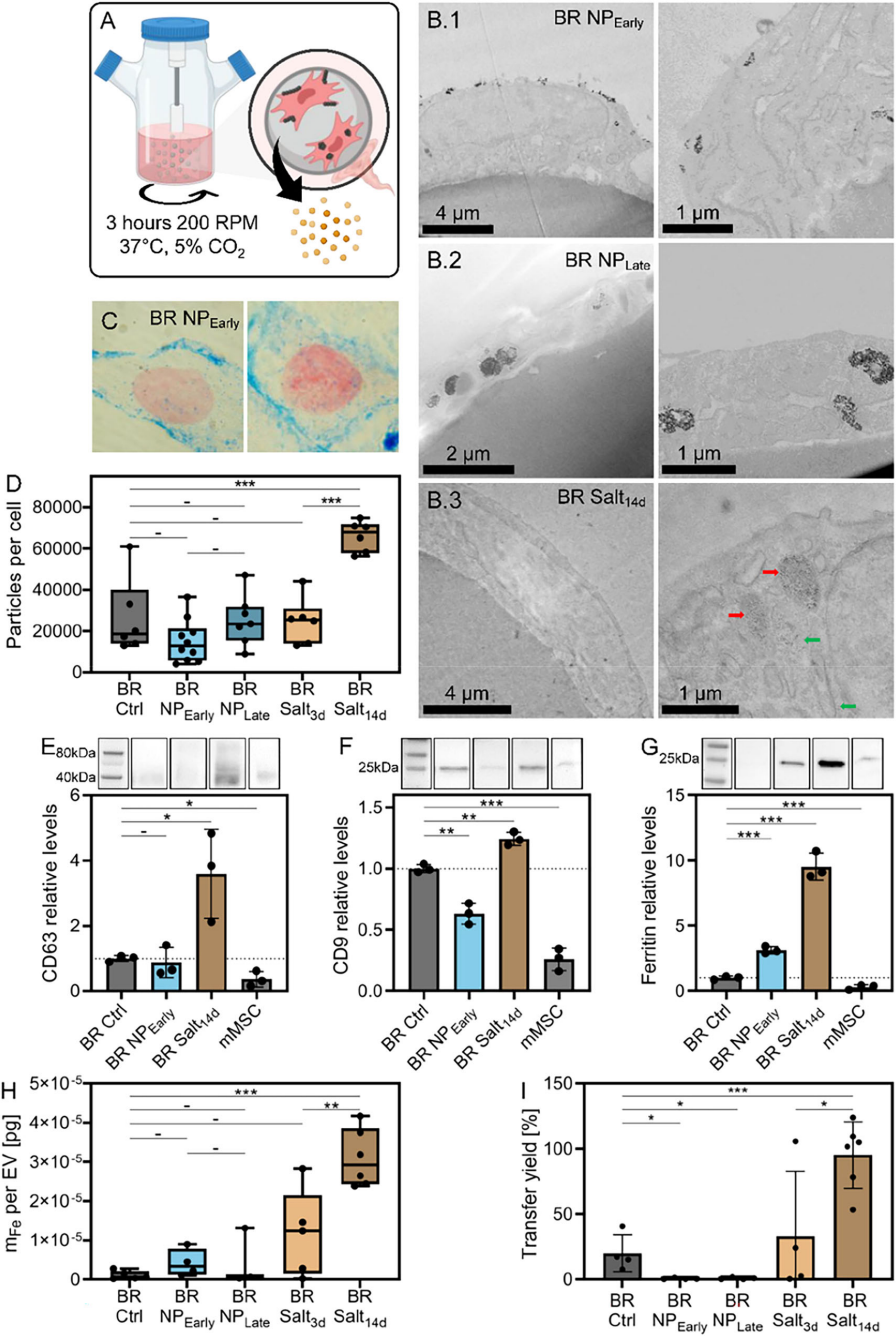
EV production from iron‐loaded MSCs through hydrodynamic turbulence. (A) Overview of the turbulence‐based EV secretion protocol. Created with BioRender.com. (B) Transmission electron microscopy imaging of mMSCs cultured on microbeads incubated with 2 mM IONPs for 30 min (BR NP_Early_) (B1), 30 min followed by a 2‐h chase (BR NP_Late_) (B2) or with 1 mM ferric salt for 14 days (BR Salt_14d_) (B3). (C) Perls’ staining of mMSCs incubated with 2 mM IONPs for 30 min (BR NP_Early_). Magnification 375×. (D) Particle yield per cell after 3 h of hydrodynamic turbulence from iron‐free mMSCs (BR Ctrl) and mMSCs incubated with 2 mM IONPs for 30 min (BR NP_Early_), 30 min followed by a 2‐h chase (BR NP_Late_) or with 1 mM ferric salt for 3 and 14 days (BR Salt_3d_ and BR Salt_14d_). (E–G) Western blot analysis of EV markers CD63 (E), CD9 (F) and ferritin (G) in EVs and parental cells, and corresponding relative levels with BR Ctrl as a reference. (H) Iron content per EV under each condition. (I) Iron transfer yield, calculated as the ratio of iron exported in EVs to intracellular iron content. Overall, these data establish that hydrodynamic turbulence dramatically scales up EV secretion rates while amplifying the targeted routing of ferritin‐bound iron into the vesicles. Each data point represents an independent experiment. Statistical significance is indicated as * = *p* < 0.05, ** = *p* < 0.01 and *** = *p* < 0.001 (two‐tailed Student's *t*‐test).

To overcome the limited yields of EV production and iron loading observed under serum starvation, particularly for IONP‐treated cells, we next explored the use of hydrodynamic stress as a scalable strategy to enhance both EV secretion and iron cargo incorporation. Using a stirred‐tank bioreactor, mMSCs were cultured on microbeads and subjected to high rotational speeds (200 rpm) for 3 h to stimulate EV release (Figure 3A). This approach not only aimed to increase EV output but also to constrain iron trafficking, especially in the context of IONP labelling, by shortening the overall incubation time and thus limiting endosomal internalization.

TEM imaging of cells cultured on microbeads confirmed our ability to modulate the subcellular localization of iron depending on the incubation protocol (Figure 3B). In the early internalization condition (BR NP_Early_), in which EV production was initiated immediately after a 30‐min IONP incubation, IONPs were predominantly found associated with the plasma membrane or localised in early endosomes (Figure 3B1, Figure S11). In contrast, when cells were left overnight in culture between IONP incubation and EV production (BR NP_Late_), IONPs were sequestered into late endosomes, as shown by their deeper positions within the cells and larger sizes, mimicking the localization observed in serum‐starved conditions (Figure 3B2, Figure S12). Ferric salt‐treated cells (BR Salt_14d_) exhibited a subcellular iron distribution identical to that observed under starvation, with electron‐dense ferritin‐like structures localised both diffusely in the cytosol (green arrows) and clustered in endosomal compartments (red arrows), as shown in Figure 3B3 (and Figure S13). Perl's Prussian blue staining further confirmed that IONPs remained near the plasma membrane in the early internalization condition (Figure 3C), supporting the efficacy of this strategy to restrict their intracellular trafficking.

After 3 h of turbulence‐induced production, particles were harvested and quantified by NTA (Figure 3D). As anticipated, turbulence stimulation significantly increases particle release compared to serum starvation. Iron‐free control cells (BR Ctrl) released an average of (2.6 ± 1.8) × 10^4^ particles per cell over just 3 h, a 5.7‐fold increase compared to 72 h of serum deprivation, or a 137‐fold enhancement when normalised per hour. Interestingly, whereas iron intracellular accumulation boosted EV secretion under starvation across all iron sources, this trend was less consistent under turbulence. Only cells preloaded with ferric salt for 14 days (BR Salt_14d_) showed a significant increase in particle production relative to the controls (2.5‐fold) and notably reduced inter‐experimental variability (11% vs. 70%). Conversely, IONP‐treated cells in the early internalization condition (BR NP_Early_) exhibited a non‐significant decrease in secretion, yielding (1.5 ± 1.0) × 10^4^ particles per cell. Notably, a similar yet non‐significant trend was observed for particle size distributions (Figure S14), with a slight increase in average size for the early IONP internalization condition and a more pronounced decrease after prolonged ferric salt incubation, thus suggesting a correlation between variations in diameters and secretion.

Western blot analysis confirmed the presence of EV markers CD63 and CD9 in particles secreted under turbulence stimulation (Figure 3E,F). Although both markers were enriched relative to parental cells, the degree of enrichment was lower than in serum‐starved EVs, 2.8‐fold versus 9.1‐fold for CD63 and 3.8‐fold versus 12.5‐fold for CD9 when compared to controls (BR Ctrl), highlighting a strong influence of the secretion trigger on vesicle composition. CD63 levels were particularly elevated following prolonged ferric salt incubation (BR Salt_14d_), with a 3.6 ± 1.4‐fold increase over the control, higher than that observed under serum starvation (2.5‐fold, Figure 2C). This reinforces the proposed link between intracellular ferritin accumulation and CD63 expression and suggests that turbulence further enhances this association. CD81 and syntenin‐1 remained undetectable (Figure S9), indicating that their absence is independent of the production method.

As in the serum starvation condition, ferritin levels in secreted particles were analysed by Western blot (Figure 3G). Ferritin was again enriched in control particles (BR Ctrl) compared to parental cells, albeit to a lesser extent than in starvation (3.3‐fold vs. 13.4‐fold). Importantly, the combined presence of EV‐specific membrane markers CD9 and CD63 with cytosolic ferritin validated that secreted particles represent genuine and intact EVs, similarly to starvation conditions. Early IONP internalization (BR NP_Early_) unexpectedly induced a significant 3.1 ± 0.3‐fold increase in ferritin levels compared to control EVs, in contrast to the downregulation observed in the serum‐starved IONP condition. Given the short experimental window, likely insufficient to allow for nanoparticle endosomal degradation into iron ions, this suggests a noncanonical interaction between IONP surface presence and ferritin regulation. As expected, prolonged ferric salt incubation (BR Salt_14d_) resulted in a remarkable increase in ferritin loading into EVs (9.5 ± 1.0‐fold), exceeding that observed under starvation (4.0 ± 1.0‐fold). These results suggest not only that ferritin remains a reliable reporter for iron content in EVs, even under dynamic conditions, but also that turbulence stimulation amplifies the ferritin cargo‐sorting process.

Quantification of vesicular iron by ICP‐MS, combined with transfer yield calculations, provided a comprehensive view on iron packaging efficiency under hydrodynamic stimulation (Figure 3H,I). In the iron‐free control (BR Ctrl) EVs contained a low but detectable amount of endogenous iron, (1.09 ± 1.06) × 10^−^^6^ pg per vesicle and corresponding to 20 ± 14% of total cellular iron, indicating that basal iron export through EVs persists independently of the secretion trigger. IONP‐treated conditions, whether involving early (BR NP_Early_) or late (BR NP_Late_) internalization, showed no significant increase in vesicular iron content compared to controls, (4.2 ± 3.5) × 10^−^^6^ and (4.7 ± 7.3) × 10^−^^6^ pg/EV, respectively. This was further confirmed by the corresponding transfer yields, which remained minimal, with 0.5 ± 0.5% for BR NP_Early_ and 0.7 ± 0.8% for BR NP_Late_, showing that nanoparticle‐based iron failed to be routed into EVs via physiological pathways under hydrodynamic stimulation. In contrast, ferric salt preloading resulted in a robust a dose‐dependent increase of both vesicular iron content and packaging efficiency. EVs derived from the 3‐day ferric salt condition contained (1.2 ± 1.1) × 10^−^^5^ pg of iron per vesicle, while those from the 14‐day condition reached (3.1 ± 0.7) × 10^−^^5^ pg/EV, values that correlated well with intracellular iron accumulation. This increased content translated into a striking 95 ± 25% transfer yield for BR Salt_14d_, indicating that nearly all cellular iron was secreted via EVs for this condition. As with the starvation protocol, variability was notably higher for the short incubation (149%) than for the prolonged condition (27%), likely reflecting the heterogeneous uptake of ferric salt for shorter incubation times.

Collectively, these results highlight the dual advantage of hydrodynamic turbulence: a strong enhancement in EV secretion and an amplification of ferric salt iron packaging into vesicles. Turbulence stimulation not only increased overall EV yield but also reinforced the link between cellular iron‐loaded ferritin accumulation and CD63 expression and, most critically, enabled near‐complete transfer of cellular iron into EVs. These results established prolonged ferric salt incubation combined with turbulence stimulation as a highly effective strategy for efficient iron transfer through EVs. In contrast, IONPs consistently failed to be efficiently sorted into vesicles, underscoring the limitations of nanoparticulate iron for EV bioengineering via physiological routes.

### 3.4 Multimodal Evidence of Selective Iron Encapsulation in EVs

**FIGURE 4:**
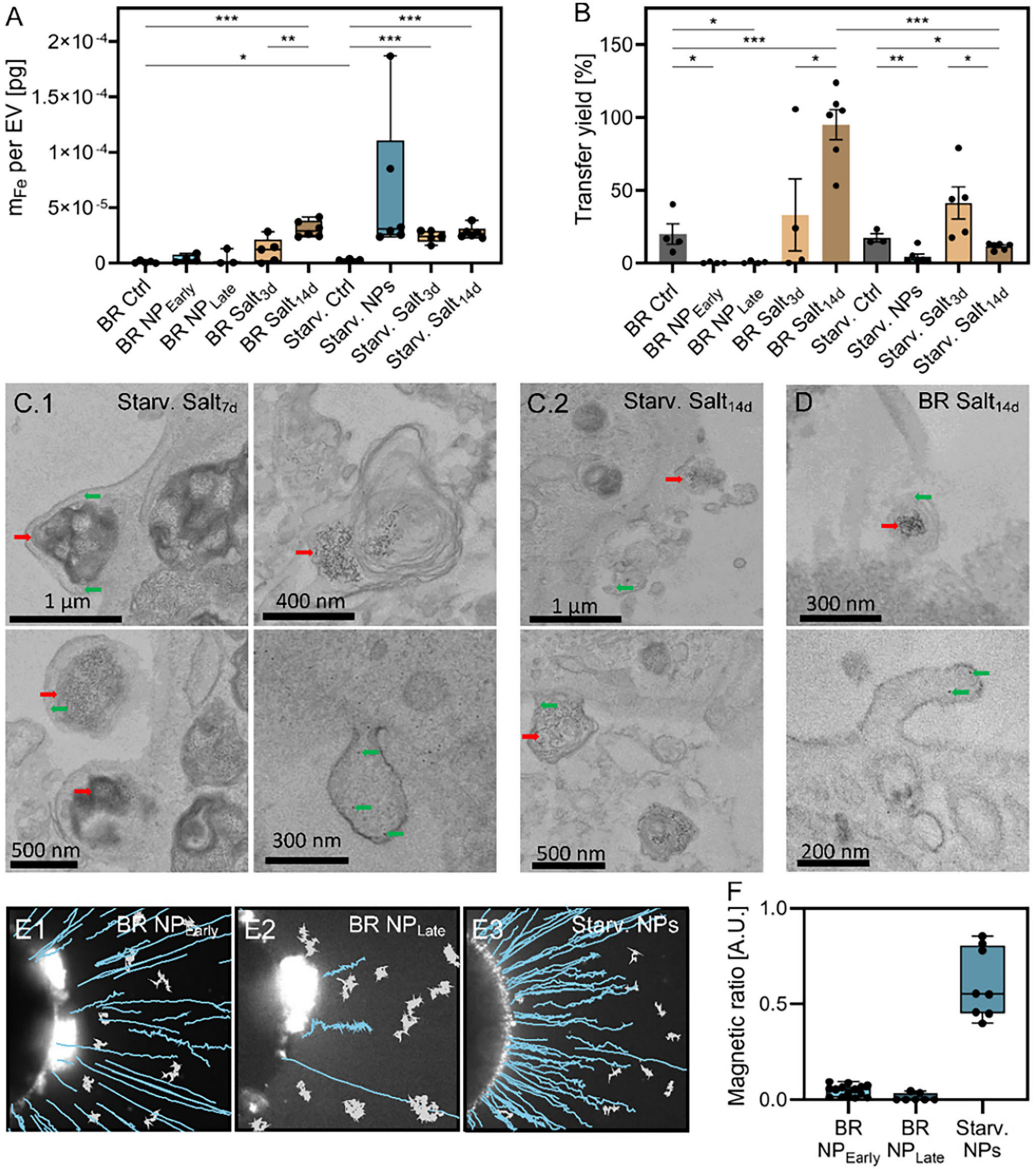
Multimodal evidence of iron encapsulation. (A) Comparative analysis of iron content in EVs released from iron‐treated mMSCs. (B) Comparative analysis of iron transfer yields for iron‐treated mMSCs. (C) Transmission electron microscopy images depicting the biogenesis of EVs from serum‐deprived mMSC monolayers incubated with 1 mM ferric salt for 7 days (C1) or 14 days (Starv. Salt_14d_) (C2). (D) Transmission electron microscopy depicting the biogenesis of EVs from turbulence‐stimulated mMSC cultured on microbeads incubated with 1 mM ferric salt for 14 days (BR Salt_14d_). (E) Magnetophoresis trajectories of EVs produced from mMSCs incubated with 2 mM IONPs for 30 min (BR NP_Early_), for 30 min followed by a 2‐h chase (BR NP_Late_) or via serum starvation after 30 min followed by a 2‐h chase (Starv. NPs) (E1–E3). Blue and gray lines represent magnetic and non‐magnetic trajectories, respectively. Scale bar = 25 µm. (F) Ratio of magnetic to non‐magnetic EVs in each condition (E). This multimodal analysis confirms that soluble iron species exploit physiological cargo‐sorting pathways for genuine endoluminal encapsulation, whereas nanoparticles remain restricted to the vesicle periphery.

The results obtained so far were compiled in a comparative analysis of vesicular iron content (Figure 4A) and iron transfer efficiency (Figure 4B) across all conditions. Ferric salt incubation, particularly after prolonged exposure, resulted in the highest and most consistent iron content per vesicle and the most efficient iron transfer, irrespective of the EV production method. Interestingly, no difference in iron content per EV was found between the starvation and turbulence conditions after prolonged exposure, suggesting that the near‐complete transfer efficiency observed in the turbulence condition was due to the drastic increase in EV secretion induced by hydrodynamic stress. In contrast, IONP‐treated cells exhibited consistently low iron transfer yields, but the vesicular iron content was found largely dependent on the mode of stimulation, with the highest, but very variable, iron content when secretion was triggered through starvation.

To investigate the mechanistic origin of these differences, EV biogenesis was examined by TEM, focusing on the presence or absence of iron‐rich structures in vesicle formation zones across the different conditions. In ferric salt‐treated cells, TEM imaging revealed the same electron‐dense structures consistent with iron‐loaded ferritin observed in Figure 1A, both dispersed in the cytosol (green arrows) and clustered into endosomal compartments (red arrows) being clearly encapsulated in budding vesicles. This transfer was observed both in starvation conditions for short (Figure 4C1 and Figure S15) and prolonged (Figure 4C2 and Figure S16) incubations and in turbulence conditions for prolonged incubation (Figure 4D and Figure S17), supporting the notion that iron‐rich ferritin is actively sorted into EVs during their formation. In contrast, IONP‐treated cells showed a complete absence of nanoparticles within sites of vesicle formation, under both serum starvation and turbulence stimulation, and across both early and late internalization protocols. This absence of IONPs in the forming vesicles stands in stark contrast to the high levels of intracellular iron quantified in these conditions and points to a failure in routing nanoparticles through the vesicle biogenesis pathways.

Although IONPs were not observed within budding EVs, serum starvation conditions still yielded a variable iron signal in EV preparations. To address this discrepancy and to further characterise this iron signal measured by ICP‐MS in starvation EVs that could also be explained by either a transfer of non‐magnetic endogenous iron or by measurement artefacts, nanomagnetophoresis analysis was conducted. Briefly, the trajectories of fluorescent EVs produced through serum starvation and turbulence stimulation in early and late internalisation conditions were recorded in the vicinity of a magnet tip to determine their magnetisation (Figure 4E). EVs produced under turbulence stimulation (Figure 4E1,E2), irrespective to the internalisation protocol, displayed only low magnetic responsiveness, as highlighted by the low number of attraction events. In contrast, EVs produced through serum starvation were significantly attracted by the magnet tip (Figure 4E3). The derivation of a magnetic ratio depicting the quantity of magnetic EVs across the different conditions (Figure 4F) further confirmed the higher abundance of magnetic iron in the starvation EVs compared to the turbulence EVs, but also allowed to show that early internalization condition led to a significantly higher, although still noticeably low, amount of magnetic EVs compared to late internalization condition. Overall, these results confirmed the IONP nature of the iron measured by ICP‐MS in the starvation condition. In the absence of direct observation of encapsulation, this magnetic signal most likely results from peripheral association of nanoparticles with EV membrane occurring either after nanoparticle exocytosis during the 3‐day time frame of serum starvation or with nanoparticles still being externally associated with the plasma membrane of the producer cells in the early turbulence condition.

Overall, these supplementary results supported the selective iron encapsulation in EVs, with ferric salt being handled by the EV cargo sorting system and successfully incorporated into vesicles, while IONPs are excluded from vesicle formation and are more likely only externally associated with already secreted EVs under starvation or early turbulence conditions.

### 3.5 Single‐Vesicle Evidence of Iron Transfer via CryoTEM and XEDS

To unambiguously confirm the encapsulation and nature of iron cargo within EVs, the vesicular iron content was investigated at the single‐particle level using cryogenic transmission electron microscopy (cryoTEM) coupled with X‐ray energy dispersive spectrometry (XEDS) after EV purification. This combination allows simultaneous visualisation of EV morphology with nanometre resolution and elemental analysis of iron, thus providing direct evidence of iron incorporation or association.

**FIGURE 5:**
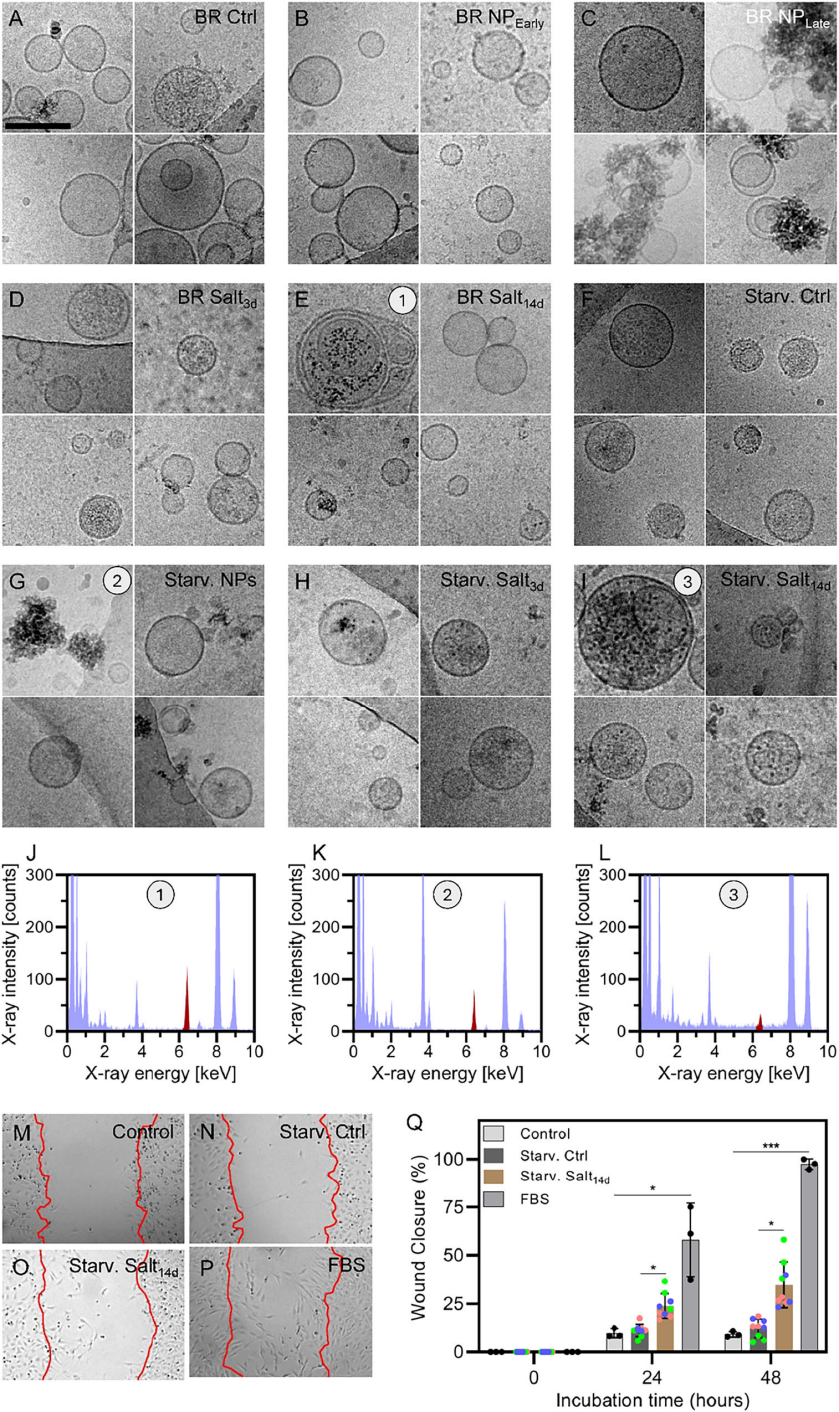
Single‐EV analysis of vesicular iron cargo. (A–E) CryoTEM of EVs produced under turbulence from untreated mMSCs (BR Ctrl) (A), or mMSCs incubated with 2 mM IONPs for 30 min (BR NP_Early_) (B), 30 min followed by a 2‐h chase (BR NP_Late_) (C), or with 1 mM ferric salt for 3 or 14 days (BR Salt_3d_, BR Salt_14d_) (D, E). The scale bar shown on A measures 150 nm. All images are to the same scale bar. (F–I) CryoTEM of EVs produced under serum starvation from untreated mMSCs (Starv. Ctrl) (F), mMSCs incubated with 2 mM IONPs for 30 min followed by a 2‐h chase (Starv. NPs) (G), or with 1 mM ferric salt for 3 or 14 days (Starv. Salt_3d_, Starv. Salt_14d_) (H, I). Scale bar is 150 nm. (J–L) XEDS spectra recorded on single EVs from BR Salt_14d_ shown in (E) (J), Starv. NPs shown in (G) (K), and Starv. Salt_14d_ shown in (I) (L). Iron Kα peaks are highlighted in red. (M–P) Bright field images of wound closure in murine fibroblasts after 24 h of incubation with serum‐free medium (Control) (M), medium supplemented with EVs produced under serum starvation from untreated mMSCs (Starv. Ctrl, 2 × 10^8^ EVs/mL) (N), medium supplemented with EVs produced under serum starvation from mMSCs incubated with 1 mM ferric salt for 14 days (Starv. Salt_14d_, 2 × 10^8^ EVs/mL) (O), and medium with 10% FBS (FBS) (P). (Q) Quantification of wound closure rate of murine fibroblasts under continuous treatment with Starv. Ctrl or Starv. Salt14d EVs (2 × 10^8^ EVs/mL). Data points for EV treatments are color‐coded by independent biological experiments (three technical replicates per independent biological experiment). Together, single‐vesicle elemental analysis and functional assay confirm the successful intraluminal encapsulation of iron‐ferritin and validate that iron‐engineered EVs retain their therapeutic wound healing potential under continuous exposure. Statistical significance is indicated as * = *p* < 0.05, ** = *p* < 0.01 and *** = *p* < 0.001 (two‐tailed Student's *t*‐test).

CryoTEM analysis confirmed the presence of intact, membrane‐bound vesicles across all conditions (Figures 5A–I and Figures S18–S26), including both single‐membrane and multi‐membrane structures. EV size distributions varied depending on production method and iron loading (Figure S27), with EVs produced through serum starvation being approximately 10% larger than those generated by turbulence stimulation, consistent with NTA measurements. All iron‐loading strategies, regardless of source or production method, resulted in smaller EVs compared to their respective controls, with the most pronounced size reduction observed after prolonged ferric salt exposure: –32.8% for serum‐starved (Starv. Salt_14d_) and –32.4% for turbulence‐derived EVs (BR Salt_14d_) EVs. Interestingly, EVs secreted after early‐stage IONP treatment followed by turbulence stimulation (BR NP_Early_) were 13.7% smaller than those produced after late‐stage treatment (BR NP_Late_), suggesting that plasma membrane crowding by surface‐bound nanoparticles may constrain vesicle formation.

CryoTEM imaging revealed distinct electron‐dense areas within or associated with EVs, except in BR NP_Early_ samples (Figure 5B and Figure S19). Two types of electron‐dense structures were distinguished. The first, observed in late‐stage IONP samples (Figure 5C and Figure S20), appeared as diffuse and clustered aggregates (3–12 nm), often membrane‐associated but not enclosed within vesicles. These aggregates were more frequent in non‐purified samples (Figures S28–S31), suggesting partial removal during size exclusion chromatography. XEDS analysis failed to detect iron in these structures, both in purified (Figure S32, spectra number 1, 4, 6 and 8) and non‐purified EV samples (Figure S33, spectra number 1, 2, 3, 4, 5, 6, 9 and 10), supporting the hypothesis that they represent proteinaceous aggregates rather than iron‐rich domains. The second type of electron‐dense structure was observed in both iron‐salt‐treated and IONP‐treated conditions. In iron salt‐treated conditions, they measured 6–8 nm (Figure 5E,I), consistent with the mineral core of iron‐loaded ferritins. In IONP‐treated samples, larger (10 nm) and more aggregated spots were detected (Figure 5G, Figure S24), corresponding to the administered nanoparticles. These electron‐dense areas persisted after purification, indicating strong EV association. XEDS analysis confirmed the presence of iron in these zones, sometimes clearly at the single vesicle level (Figure 5J–L). No electron‐dense iron‐containing areas were detected in EVs from iron‐free control conditions (Figure 5A,F, Figures S18 and S23).

In IONP conditions, iron‐rich zones were detected only in EVs collected after serum starvation, both in purified (Figure 5G,K) and non‐purified (Figure S31, spectrum number 19) samples. However, these iron deposits always adhered to the outer EV membrane rather than enclosed within the lumen. This confirms the absence of nanoparticle internalization within vesicles and further supports that the iron signal measured in IONP‐EVs via ICP‐MS or nanomagnetophoresis originates from surface‐bound nanoparticles or residual extracellular aggregates. Furthermore, no iron‐related electron‐dense areas were found in EVs produced under turbulence‐stimulation conditions after IONP treatment (Figure 5B,D, Figures S19 and S20).

In contrast, cryoTEM and XEDS consistently revealed electron‐dense ferritin‐like cores within EVs produced after prolonged iron salt treatment, both in turbulence (Figure 5E,J, Figures S22 and S32) and serum starvation (Figure 5I,L, Figures S26 and S32) conditions. Iron‐rich ferritin cores were also identified in non‐purified EVs after short‐term salt incubation (Figures S30 and S33), and suspected, though not confirmed by XEDS, in short‐term purified samples, both under turbulence stimulation (Figure 5D and Figure S21) and serum starvation (Figure 5H and Figure S25). Additionally, ferritin cores were observed in both single‐membrane and multi‐membrane vesicles.

Altogether, this multimodal analysis provided conclusive evidence that EVs secreted after prolonged ferric salt exposure encapsulate iron in the form of ferritin. Conversely, IONPs are excluded from vesicle lumens and remain adsorbed at the membrane periphery, failing to exploit the physiological cargo‐sorting mechanisms for encapsulation. These findings strengthen the notion that only soluble iron species can be effectively routed into EVs via natural pathways.

To assess whether the functional properties of the secreted vesicles were altered or enhanced by the iron salt cargo, EVs produced via serum starvation were evaluated for their capacity to promote cell migration and wound closure using a two‐dimensional in vitro scratch assay on murine fibroblast (Figure 5M‐Q and Figure S34). As anticipated, scratch clearance in fibroblasts maintained under continuous incubation with serum‐free control medium (Figure 5M) was severely restricted, reaching only 9.7 ± 2.3% after 24 h and 9.2 ± 1.5% after 48 h, alongside noticeable cell death. Conversely, the scratch wound was almost fully resolved after 48 h of incubation with complete, serum‐supplemented medium (Figure 5P). Native, iron‐free EVs produced via starvation (Starv. Ctrl) (Figure 5N) failed to rescue the absence of serum factors, yielding closure rates similar to the negative control group (11.1 ± 3.1% at 24 h, and 12.2 ± 4.8% at 48 h). In striking contrast, continuous exposure to iron‐loaded EVs (Starv. Salt14d) (Figure 5O) partially rescued the absence of nutrients and allowed a 23.7 ± 6.4% closure at 24 h, and 34.7 ± 11.8% at 48 h. These results demonstrate that the encapsulated vesicular iron load is successfully transferred to recipient cells and functionally metabolised upon co‐incubation, confirming the potential of these iron‐engineered EVs to act as biologically active iron transporters.

## 4 Discussion

The present study provides evidence for a high‐efficiency method to produce iron‐loaded EVs through a combination of turbulence stimulation and prolonged ferric salt incubation. Besides, this investigation sheds light on iron trafficking, from cellular uptake to EV transfer, highlighting its reliance on various factors, including the source of iron, the incubation duration, and the stimulation methods employed for EV production.

Consistent with the literature (Truman‐Rosentsvit et al. 2018; Chen and Hou 2023; Lee et al. 2020), nanoparticles (IONPs) were first internalised by cells followed by their localisation in intracellular vesicles. Distinguishing specific types of intracellular vesicle, such as endosomes, multivesicular bodies or lysosomes, remains challenging due to their similar appearance in TEM imaging. Indeed, subcellular iron localisation and loading efficiency are expected to be highly dependent on the incubation conditions. It is expected that IONPs internalisation will occur through the clathrin‐mediated endocytosis pathway (Chen and Hou 2023; Lee et al. 2020), with an early storage stage potentially on outer plasma membrane and in early endosomes (BR NP_Early_), and a late storage stage in late endosomes and lysosomes (BR NP_Late_, Starv. NPs).

**FIGURE 6:**
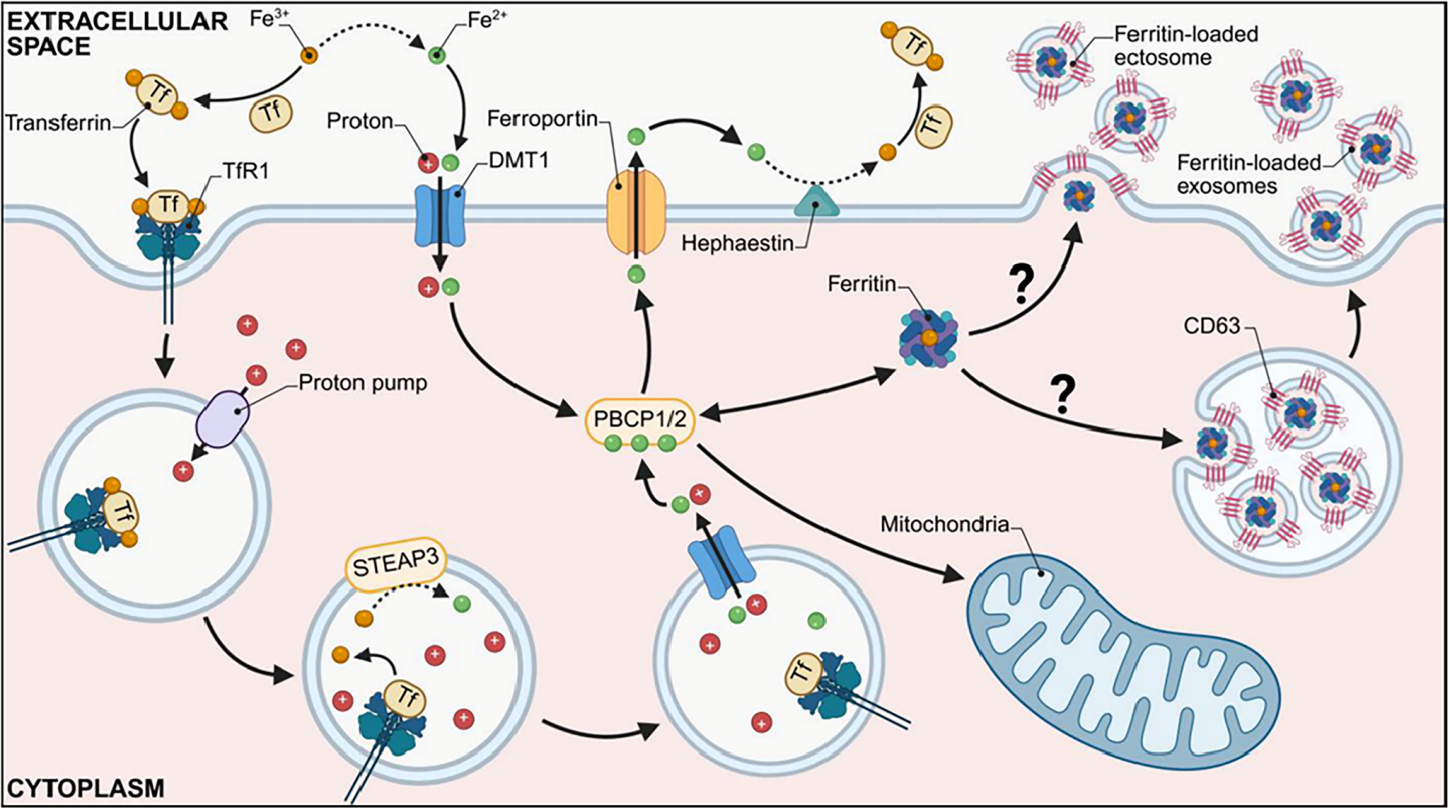
Schematic model of the intracellular routes of iron ions and EV‐mediated ferritin secretion pathway. Soluble ferric ions (Fe^3^^+^), internalised via transferrin‐dependent or independent routes, accumulate in endosomal compartments and are sequestered into ferritin. Exposure leads to ferritin routing to multivesicular bodies and secretion in CD63+ EVs, resulting in iron encapsulation within ferritin‐containing vesicles. Created with BioRender.com.

In contrast, iron salts enter the intracellular space via two distinct mechanisms depending on their oxidation state (Figure 6) (Vogt et al. 2021). Ferric iron (Fe^3^^+^), such as in ferric quinate, is internalised through receptor‐mediated endocytosis. First, ferric iron binds to extracellular transferrin, which interacts with transferrin receptor 1 (TfR1) on the cell membrane. After internalisation into endosomes, the decreasing pH within late endosomes causes ferric iron to dissociate from transferrin. STEAP3 proteins then reduce Fe^3^^+^ to ferrous iron (Fe^2^^+^), which is transported into the cytosol by divalent metal transporter 1 (DMT1). Once in the cytosol, Fe^2^^+^ can be utilised in cellular metabolic processes, exported or stored in cytosolic ferritin. Alternatively, ferric quinate may be reduced to Fe^2^^+^ in the extracellular space by reducing agents. In this case, Fe^2^^+^ is directly internalised into the cytosol through the DMT1 pathway, bypassing the endocytic route (Vogt et al. 2021).

**FIGURE 7:**
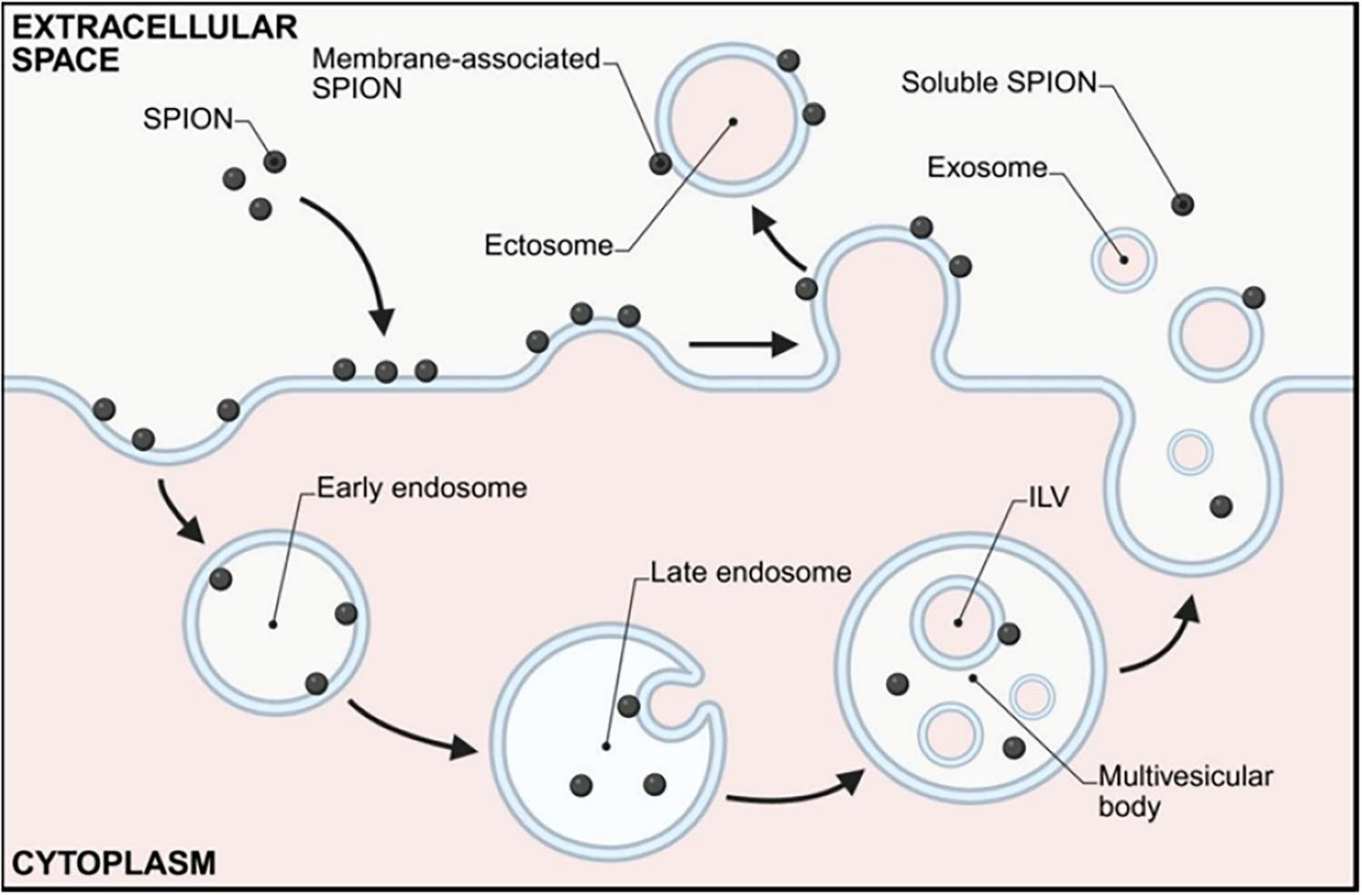
Schematic representation of topological analysis of IONP routes and EV secretion pathways. IONPs associate with the plasma membrane and are internalised in endosomal compartments but remain excluded from intraluminal vesicles (ILV) and ectosomes. EVs produced under these conditions carry surface‐bound IONPs without internal encapsulation, leading to minimal iron transfer via EVs. Created with BioRender.com.

Our observation that IONPs are not internalised into EVs represents a significant divergence from some prior studies, including ours (Silva et al. 2012, 2013, 2015), which suggested successful encapsulation. This discrepancy may also arise from differences in experimental protocols. For instance, some of our previous studies were based on magnetic sorting for isolating iron‐loaded EVs (Silva et al. 2012, 2013, 2015). Other studies were based on electroporation approach or extrusion method to produce IONP‐loaded EVs and EV‐like particles, respectively (Hu et al. 2015; Kim et al. 2020). In fact, topological considerations would suggest that IONPs need to be localised in (i) intra‐luminal vesicles within multi‐vesicular bodies to end up in small EVs/exosomes or (ii) in the cytosol to be sorted into microvesicles through cytosolic trafficking, both options being prevented by the inability of IONPs to cross biological barriers due to their size (Figure 7). In contrast, iron‐loaded ferritins were abundantly observed, both directly in the cytosol and within endosomal compartments, possibly multi‐vesicular bodies or secretory autolysosomes, considering the extended incubation durations for ferric salt conditions (Truman‐Rosentsvit et al. 2018). This could explain the striking 95% transfer of intracellular iron into EVs for the combination of turbulence stimulation and prolonged incubation with ferric salt in contrast to the negligible transfer for the conditions in which incubation was performed with IONP. To address IONP encapsulation into EVS, future work could explore endosomal escape strategies, which may enable cytosolic localization of IONPs (Smith et al. 2019). Such an approach could allow IONP encapsulation within EVs and open up new possibilities for EV engineering. Importantly, the multimodal evidence presented in this work provides strong support for a clear distinction between iron oxide nanoparticles and ferritin‐associated iron. Beyond structural and elemental analyses, magnetic susceptibility–based approaches have been shown to enable quantitative discrimination between different iron species (Fernández‐Afonso et al. 2022). Integration of such orthogonal magnetic methods in future studies could therefore provide an independent validation of iron speciation across experimental conditions.

The substantial transfer of 95% of intracellular iron into EVs through the combination of turbulence stimulation and prolonged incubation with ferric salt can also be attributed to the significant increase in EV release associated with turbulence stimulation. As shown above, the hourly rate of EV secretion increased of a factor of 137 with turbulence stimulation compared to starvation conditions. While some of our previous studies have reported the use of the turbulence method to produce anti‐tumoural drug‐loaded EVs (Millard et al. 2020; Pinto et al. 2021), this is the first time we present the design of iron‐loaded EVs utilising turbulence stimulation. Furthermore, our data indicates the presence of iron‐loaded ferritins within EVs. While previous literature has reported EVs enriched with iron stored in ferritin complexes (van Meteren et al. 2020), this study is the first to demonstrate massive EV production alongside simultaneous high‐yield iron transfer associated to ferritin.

We also investigated the effect of the source of iron, the incubation duration, and the stimulation methods employed for EV production on EV markers. EVs produced via serum starvation exhibited higher expression levels of CD9, CD63 and ferritin markers compared to those produced through turbulence stimulation. This may be attributed to a different biogenesis mechanism and could also relate to the fact that the total pool of these cellular markers is shed in 137 times more EVs under turbulence conditions, which mathematically reduces the total amount of protein per EV. Consistent with existing literature (Yanatori et al. 2021), CD63 vesicular level was increased for iron‐treated cells (Figure 6). This increase was more pronounced with ferric salt treatment with a positive correlation with the incubation duration. In fact, it is known that iron regulates CD63 expression through iron regulatory elements (IREs) present at CD63 corresponding mRNA (Yanatori et al. 2021). However, the exact molecular mechanism linking ferritin‐stored iron accumulation, CD63 expression and secretion of CD63 positive iron‐loaded EVs remains to be elucidated.

Iron‐loaded EVs have been extensively investigated for magnetic drug targeting in the case of IONP‐loaded EVs. These applications encompass oncotherapy, treatments for spinal cord injuries, ischemic stroke and cardiac repair for a localised and enhanced drug delivery, as reviewed elsewhere (Zhuo et al. 2021). Therefore, a specific limitation of the bioprocess presented herein is that the resulting iron‐engineered EVs do not exhibit magnetic properties. Because the iron is integrated in the vesicles as physiological ferritin complexes rather than iron oxide nanoparticles, these vesicles lack the high magnetic susceptibility required for active magnetic drug targeting or contrast enhancement. Second, this work was strictly conducted using murine cell models. Translating these bioprocesses from mouse to human will be mandatory to accurately assess the clinical and translational therapeutic potential of these vesicles. Nevertheless, these current limitations do not compromise the biological value of our system, as demonstrated by our in vitro wound healing assay. Indeed, continuous exposure to these iron‐rich, non‐magnetic EVs significantly accelerated fibroblast migration and enhanced scratch wound closure compared to native EV controls. This pivotal finding provides direct evidence that the encapsulated intraluminal iron‐ferritin cargo is successfully internalised, bio‐available and functionally metabolised by recipient cells, highlighting their remarkable potential as biologically active iron transporters for tissue regeneration. Beyond regenerative medicine, targeting cell‐recipient iron trafficking pathways opens up other distinct therapeutic avenues. For instance, while EVs containing iron‐loaded ferritin can transport iron in a safe state, their accumulation inside specific target cells could be exploited to induce localised oxidative DNA damage for anti‐tumour therapy (van Meteren et al. 2020). Furthermore, such massive intraluminal iron delivery could contribute to the induction of ferroptosis in cancer cells, triggering catalytic Fe(II)‐dependent necrosis coupled with lipid peroxidation (Stockwell et al. 2017). Consequently, the evaluation of the functional biological activity of these vesicles, although still limited, shifts the paradigm of iron‐engineering from a physical targeting tool to a highly potent metabolic biotherapy.

Overall, the present results demonstrate a highly efficient method to produce iron‐loaded EVs without the need for post‐production modifications, combining turbulence stimulation with prolonged ferric salt incubation of parental cells. Using this iron‐engineering approach, we achieved over 60,000 EVs per cell within 3 h of stimulation, with 95% of intracellular iron successfully transferred into the EVs. The technological advancements presented in this study are essential for the development of scalable methods to produce iron‐loaded EVs, which could serve as innovative biotherapies targeting iron trafficking. Beyond their bioproduction implications, these findings provide valuable insights into iron trafficking, from cellular uptake to EV‐mediated transfer, highlighting its dependence on iron source, concentration, incubation duration and stimulation method. Ultimately, this investigation deepens our understanding of the intricate interplay between cells and EVs in regulating intracellular iron storage.

## Author Contributions

**Elliot Thouvenot**: methodology, conceptualization, investigation, software, validation, visualization, writing – original draft, writing – review and editing, data curation. **Aurore Van de Walle**: conceptualization, validation, supervision, writing – review and editing, methodology. **Jean‐Michel Guigner**: writing – review and editing, investigation, resources. **Ali Abou‐Hassan**: writing – review and editing, resources. **Lorena Martin‐Jaular**: writing – review and editing, methodology, investigation, resources. **Amanda K. A. Silva**: conceptualization, writing – review and editing, formal analysis, methodology, validation. **Claire Wilhelm**: conceptualization, formal analysis, funding acquisition, project administration, supervision, resources, validation, visualization, writing – original draft, investigation, writing – review and editing.

## Conflicts of Interest

The authors declare no conflict of interest.

## Geolocation Information

This study was conducted in Paris, France. Cell culture, extracellular vesicle production and biochemical analyses were performed at Institut Curie. Cryo‐electron microscopy and elemental analyses were carried out at Sorbonne Université‐associated facilities, France. Inductively coupled plasma measurements were performed at the Institut de Physique du Globe de Paris (IPGP), Université Paris Cité.

## Supporting information




**Supplementary Figures**: jev270346‐sup‐0001‐Figures.pdf

## Data Availability

The data that support the findings of this study are available from the corresponding author upon reasonable request.

## References

[jev270346-bib-0001] Andriola Silva, A. K. , R. Di Corato , T. Pellegrino , et al. 2013. “Cell‐Derived Vesicles as a Bioplatform for the Encapsulation of Theranostic Nanomaterials.” Nanoscale 5: 11374. 10.1039/c3nr01541f. 23827988

[jev270346-bib-0002] Arasu, U. T. , R. Kärnä , K. Härkönen , et al. 2017. “Human Mesenchymal Stem Cells Secrete Hyaluronan‐Coated Extracellular Vesicles.” Matrix Biology 64: 54–68. 10.1016/j.matbio.2017.05.001. 28483644

[jev270346-bib-0003] Azparren‐Angulo, M. , F. Royo , E. Gonzalez , et al. 2021. “Extracellular Vesicles in Hepatology: Physiological Role, Involvement in Pathogenesis, and Therapeutic Opportunities.” Pharmacology & Therapeutics 218: 107683. 10.1016/j.pharmthera.2020.107683. 32961265

[jev270346-bib-0004] Beola, L. , L. Asín , R. M. Fratila , et al. 2018. “Dual Role of Magnetic Nanoparticles as Intracellular Hotspots and Extracellular Matrix Disruptors Triggered by Magnetic Hyperthermia in 3D Cell Culture Models.” ACS Applied Materials & Interfaces 10: 44301–44313. 10.1021/acsami.8b18270. 30480993

[jev270346-bib-0005] Berger, A. , I. Araújo‐Filho , M. Piffoux , et al. 2021. “Local Administration of Stem Cell‐Derived Extracellular Vesicles in a Thermoresponsive Hydrogel Promotes a Pro‐Healing Effect in a Rat Model of Colo‐Cutaneous Post‐Surgical Fistula.” Nanoscale 13: 218–232. 10.1039/D0NR07349K. 33326529

[jev270346-bib-0006] Bruno, S. , C. Grange , F. Collino , et al. 2012. “Microvesicles Derived From Mesenchymal Stem Cells Enhance Survival in a Lethal Model of Acute Kidney Injury.” PLoS ONE 7: e33115. 10.1371/journal.pone.0033115. 22431999 PMC3303802

[jev270346-bib-0007] Cabana, S. , A. Curcio , A. Michel , C. Wilhelm , and A. Abou‐Hassan . 2020. “Iron Oxide Mediated Photothermal Therapy in the Second Biological Window: A Comparative Study Between Magnetite/Maghemite Nanospheres and Nanoflowers.” Nanomaterials 10: 1548. 10.3390/nano10081548. 32784579 PMC7466508

[jev270346-bib-0008] Chen, Y. , and S. Hou . 2023. “Recent Progress in the Effect of Magnetic Iron Oxide Nanoparticles on Cells and Extracellular Vesicles.” Cell Death Discovery 9: 195. 10.1038/s41420-023-01490-2. 37380637 PMC10307851

[jev270346-bib-0009] Couch, Y. , E. I. Buzàs , D. Di Vizio , et al. 2021. “A Brief History of Nearly EV‐Erything—The Rise and Rise of Extracellular Vesicles.” Journal of Extracellular Vesicles 10: e12144. 10.1002/jev2.12144. 34919343 PMC8681215

[jev270346-bib-0010] Danilushkina, A. A. , C. C. Emene , N. A. Barlev , and M. O. Gomzikova . 2023. “Strategies for Engineering of Extracellular Vesicles.” International Journal of Molecular Sciences 24: 13247. 10.3390/ijms241713247. 37686050 PMC10488046

[jev270346-bib-0011] de Abreu, R. C. , C. V. Ramos , C. Becher , et al. 2021. “Exogenous Loading of miRNAs Into Small Extracellular Vesicles.” Journal of Extracellular Vesicles 10: e12111. 10.1002/jev2.12111. 34377372 PMC8329988

[jev270346-bib-0012] Espinosa, A. , J. Kolosnjaj‐Tabi , A. Abou‐Hassan , et al. 2018. “Magnetic (Hyper)Thermia or Photothermia? Progressive Comparison of Iron Oxide and Gold Nanoparticles Heating in Water, in Cells, and In Vivo.” Advanced Functional Materials 28: 1803660. 10.1002/adfm.201803660.

[jev270346-bib-0013] Fernández‐Afonso, Y. , L. Asín , L. Beola , et al. 2022. “Iron Speciation in Animal Tissues Using AC Magnetic Susceptibility Measurements: Quantification of Magnetic Nanoparticles, Ferritin, and Other Iron‐Containing Species.” ACS Applied Bio Materials 5: 1879–1889. 10.1021/acsabm.1c01200. PMC9115797 35179873

[jev270346-bib-0014] Frankel, R. B. , R. P. Blakemore , and R. S. Wolfe . 1979. “Magnetite in Freshwater Magnetotactic Bacteria.” Science 203: 1355–1356. 10.1126/science.203.4387.1355. 17780480

[jev270346-bib-0015] Fromain, A. , J. E. Perez , A. Van De Walle , Y. Lalatonne , and C. Wilhelm . 2023. “Photothermia at the Nanoscale Induces Ferroptosis via Nanoparticle Degradation.” Nature Communications 14: 4637. 10.1038/s41467-023-40258-1. PMC10397343 37532698

[jev270346-bib-0016] Galy, B. , M. Conrad , and M. Muckenthaler . 2024. “Mechanisms Controlling Cellular and Systemic Iron Homeostasis.” Nature Reviews Molecular Cell Biology 25: 133–155. 10.1038/s41580-023-00648-1. 37783783

[jev270346-bib-0017] Grangier, A. , J. Branchu , J. Volatron , et al. 2021. “Technological Advances Towards Extracellular Vesicles Mass Production.” Advanced Drug Delivery Reviews 176: 113843. 10.1016/j.addr.2021.113843. 34147532

[jev270346-bib-0018] Hendrix, A. , L. Lippens , C. Pinheiro , et al. 2023. “Extracellular Vesicle Analysis.” Nature Reviews Methods Primers 3: 56. 10.1038/s43586-023-00240-z. PMC12968065 41809514

[jev270346-bib-0019] Hu, L. , S. A. Wickline , and J. L. Hood . 2015. “Magnetic Resonance Imaging of Melanoma Exosomes in Lymph Nodes.” Magnetic Resonance in Medicine 74: 266–271. 10.1002/mrm.25376. 25052384 PMC4422779

[jev270346-bib-0020] Kang, M. , V. Jordan , C. Blenkiron , and L. W. Chamley . 2021. “Biodistribution of Extracellular Vesicles Following Administration Into Animals: A Systematic Review.” Journal of Extracellular Vesicles 10: e12085. 10.1002/jev2.12085. 34194679 PMC8224174

[jev270346-bib-0021] Kim, E. H. , J. P. Son , G. S. Oh , et al. 2025. “Clinical Scale MSC‐Derived Extracellular Vesicles Enhance Poststroke Neuroplasticity in Rodents and Non‐Human Primates.” Journal of Extracellular Vesicles 14: e70110. 10.1002/jev2.70110. 40545933 PMC12183388

[jev270346-bib-0022] Kim, H. Y. , T. J. Kim , L. Kang , et al. 2020. “Mesenchymal Stem Cell‐Derived Magnetic Extracellular Nanovesicles for Targeting and Treatment of Ischemic Stroke.” Biomaterials 243: 119942. 10.1016/j.biomaterials.2020.119942. 32179302

[jev270346-bib-0023] Lai, R. C. , F. Arslan , M. M. Lee , et al. 2010. “Exosome Secreted by MSC Reduces Myocardial Ischemia/Reperfusion Injury.” Stem Cell Research 4: 214–222. 10.1016/j.scr.2009.12.003. 20138817

[jev270346-bib-0024] Lee, S. H. , D. J. Park , W. S. Yun , et al. 2020. “Endocytic Trafficking of Polymeric Clustered Superparamagnetic Iron Oxide Nanoparticles in Mesenchymal Stem Cells.” Journal of Controlled Release 326: 408–418. 10.1016/j.jconrel.2020.07.032. 32711024

[jev270346-bib-0025] Manno, M. , A. Bongiovanni , L. Margolis , P. Bergese , and P. Arosio . 2025. “The Physico‐Chemical Landscape of Extracellular Vesicles.” Nature Reviews Bioengineering 3: 68–82. 10.1038/s44222-024-00255-5.

[jev270346-bib-0026] Mathieu, M. , L. Martin‐Jaular , G. Lavieu , and C. Théry . 2019. “Specificities of Secretion and Uptake of Exosomes and Other Extracellular Vesicles for Cell‐to‐Cell Communication.” Nature Cell Biology 21: 9–17. 10.1038/s41556-018-0250-9. 30602770

[jev270346-bib-0027] Millard, M. , S. Posty , M. Piffoux , et al. 2020. “mTHPC‐Loaded Extracellular Vesicles Significantly Improve mTHPC Diffusion and Photodynamic Activity in Preclinical Models.” Pharmaceutics 12: 676. 10.3390/pharmaceutics12070676. 32709026 PMC7407764

[jev270346-bib-0028] Pinto, A. , I. Marangon , J. Méreaux , et al. 2021. “Immune Reprogramming Precision Photodynamic Therapy of Peritoneal Metastasis by Scalable Stem‐Cell‐Derived Extracellular Vesicles.” ACS Nano 15: 3251–3263. 10.1021/acsnano.0c09938. 33481565

[jev270346-bib-0029] Sant'Angelo, D. , G. Descamps , V. Lecomte , et al. 2025. “Therapeutic Approaches With Iron Oxide Nanoparticles to Induce Ferroptosis and Overcome Radioresistance in Cancers.” Pharmaceuticals 18: 325. 10.3390/ph18030325. 40143107 PMC11945075

[jev270346-bib-0030] Silva, A. K. A. , J. Kolosnjaj‐Tabi , S. Bonneau , et al. 2013. “Magnetic and Photoresponsive Theranosomes: Translating Cell‐Released Vesicles Into Smart Nanovectors for Cancer Therapy.” ACS Nano 7: 4954–4966. 10.1021/nn400269x. 23641799

[jev270346-bib-0031] Silva, A. K. A. , N. Luciani , F. Gazeau , et al. 2015. “Combining Magnetic Nanoparticles With Cell Derived Microvesicles for Drug Loading and Targeting.” Nanomedicine: Nanotechnology, Biology and Medicine 11: 645–655. 10.1016/j.nano.2014.11.009. 25596340

[jev270346-bib-0032] Silva, A. K. A. , C. Wilhelm , J. Kolosnjaj‐Tabi , N. Luciani , and F. Gazeau . 2012. “Cellular Transfer of Magnetic Nanoparticles via Cell Microvesicles: Impact on Cell Tracking by Magnetic Resonance Imaging.” Pharmaceutical Research 29: 1392–1403. 10.1007/s11095-012-0680-1. 22271049

[jev270346-bib-0033] Smith, S. A. , L. I. Selby , A. P. R. Johnston , and G. K. Such . 2019. “The Endosomal Escape of Nanoparticles: Toward More Efficient Cellular Delivery.” Bioconjugate Chemistry 30: 263–272. 10.1021/acs.bioconjchem.8b00732. 30452233

[jev270346-bib-0034] Stanicki, D. , T. Vangijzegem , I. Ternad , and S. Laurent . 2022. “An Update on the Applications and Characteristics of Magnetic Iron Oxide Nanoparticles for Drug Delivery.” Expert Opinion on Drug Delivery 19: 321–335. 10.1080/17425247.2022.2047020. 35202551

[jev270346-bib-0035] Staniland, S. , B. Ward , A. Harrison , G. van der Laan , and N. Telling . 2007. “Rapid Magnetosome Formation Shown by Real‐Time X‐Ray Magnetic Circular Dichroism.” Proceedings of the National Academy of Sciences 104: 19524–19528. 10.1073/pnas.0704879104. PMC2148322 18032611

[jev270346-bib-0036] Stockwell, B. R. , J. P. Friedmann Angeli , H. Bayir , et al. 2017. “Ferroptosis: A Regulated Cell Death Nexus Linking Metabolism, Redox Biology, and Disease.” Cell 171: 273–285. 10.1016/j.cell.2017.09.021. 28985560 PMC5685180

[jev270346-bib-0037] Tatischeff, I. , and A. Alfsen . 2011. “A New Biological Strategy for Drug Delivery: Eucaryotic Cell‐Derived Nanovesicles.” Journal of Biomaterials and Nanobiotechnology 02: 494–499. 10.4236/jbnb.2011.225060.

[jev270346-bib-0038] Truman‐Rosentsvit, M. , D. Berenbaum , L. Spektor , et al. 2018. “Ferritin Is Secreted via 2 Distinct Nonclassical Vesicular Pathways.” Blood 131: 342–352. 10.1182/blood-2017-02-768580. 29074498 PMC5774206

[jev270346-bib-0039] van Meteren, N. , D. Lagadic‐Gossmann , N. Podechard , et al. 2020. “Extracellular Vesicles Released by Polycyclic Aromatic Hydrocarbons‐Treated Hepatocytes Trigger Oxidative Stress in Recipient Hepatocytes by Delivering Iron.” Free Radical Biology and Medicine 160: 246–262. 10.1016/j.freeradbiomed.2020.08.001. 32791186

[jev270346-bib-0040] Vogt, A.‐C. S. , T. Arsiwala , M. Mohsen , M. Vogel , V. Manolova , and M. F. Bachmann . 2021. “On Iron Metabolism and Its Regulation.” International Journal of Molecular Sciences 22: 4591. 10.3390/ijms22094591. 33925597 PMC8123811

[jev270346-bib-0041] Wang, W. , Z. Han , S. Aafreen , et al. 2025. “Magnetically Labelled iPSC‐Derived Extracellular Vesicles Enable MRI/MPI‐Guided Regenerative Therapy for Myocardial Infarction.” Journal of Extracellular Vesicles 14: e70178. 10.1002/jev2.70178. 41063447 PMC12508251

[jev270346-bib-0042] Welsh, J. A. , D. C. I. Goberdhan , L. O'Driscoll , et al. 2024. “Minimal Information for Studies of Extracellular Vesicles (MISEV2023): From Basic to Advanced Approaches.” Journal of Extracellular Vesicles 13: e12404. 10.1002/jev2.12404. 38326288 PMC10850029

[jev270346-bib-0043] Yanatori, I. , D. R. Richardson , H. S. Dhekne , S. Toyokuni , and F. Kishi . 2021. “CD63 is Regulated by Iron via the IRE‐IRP System and is Important for Ferritin Secretion by Extracellular Vesicles.” Blood 138: 1490–1503. 10.1182/blood.2021010995. 34265052 PMC8667049

[jev270346-bib-0044] Yáñez‐Mó, M. , P. R.‐M. Siljander , Z. Andreu , et al. 2015. “Biological Properties of Extracellular Vesicles and Their Physiological Functions.” Journal of Extracellular Vesicles 4: 27066. 10.3402/jev.v4.27066. 25979354 PMC4433489

[jev270346-bib-0045] Zhang, B. , M. Wang , A. Gong , et al. 2015. “HucMSC‐Exosome Mediated‐Wnt4 Signaling Is Required for Cutaneous Wound Healing.” Stem Cells 33: 2158–2168. 10.1002/stem.1771. 24964196

[jev270346-bib-0046] Zhuo, Z. , J. Wang , Y. Luo , et al. 2021. “Targeted Extracellular Vesicle Delivery Systems Employing Superparamagnetic Iron Oxide Nanoparticles.” Acta Biomaterialia 134: 13–31. 10.1016/j.actbio.2021.07.027. 34284151

