# Supplementary material for: Iron Salts, but Not Iron Oxide Nanoparticles, Drive High‐Yield Production of Iron‐Engineered Extracellular Vesicles
Source: J Extracell Vesicles. 2026 Aug 9;15(8):e70346. doi: 10.1002/jev2.70346 (PMC13453940; doi:10.1002/jev2.70346)
Supplement: Supplementary file 1 [file JEV2-15-e70346-s001.pdf]

Iron salts, but not iron oxide nanoparticles, drive high-yield production of iron-engineered extracellular vesicles

Elliot Thouvenot[1], Aurore Van de Walle[1], Jean-Michel Guigner[2], Ali Abou-Hassan[3,4], Lorena Martin-Jaular[5,6], Amanda K. A. Silva[7], Claire Wilhelm[1]*

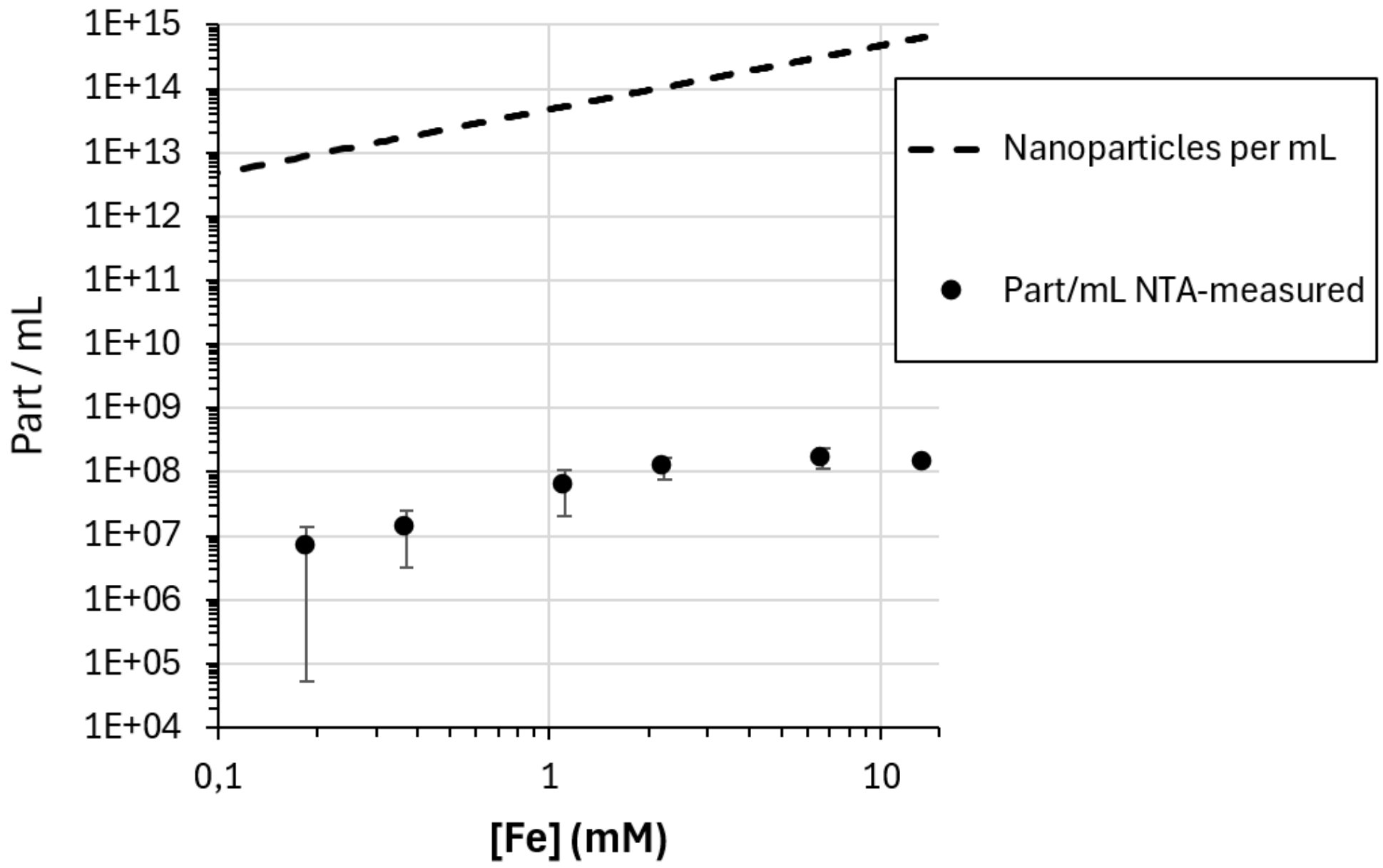

Figure S1. Iron oxide nanoparticles contribution to NTA measurements: NTA was performed on iron oxide nanoparticle suspensions prepared in the same culture medium used throughout the study, across a range of iron concentrations. No particles were detected below 0.2 mM iron. At higher concentrations, a detectable signal increased with nanoparticle concentration. However, the measured particle concentrations remained approximately six orders of magnitude lower than the actual nanoparticle concentrations in solution. Importantly, under the experimental conditions used throughout this study, iron concentrations in samples analyzed by NTA were systematically below 0.1 mM and typically in the low micromolar range. The contribution of free iron oxide nanoparticles to NTA-based particle quantification is therefore negligible.

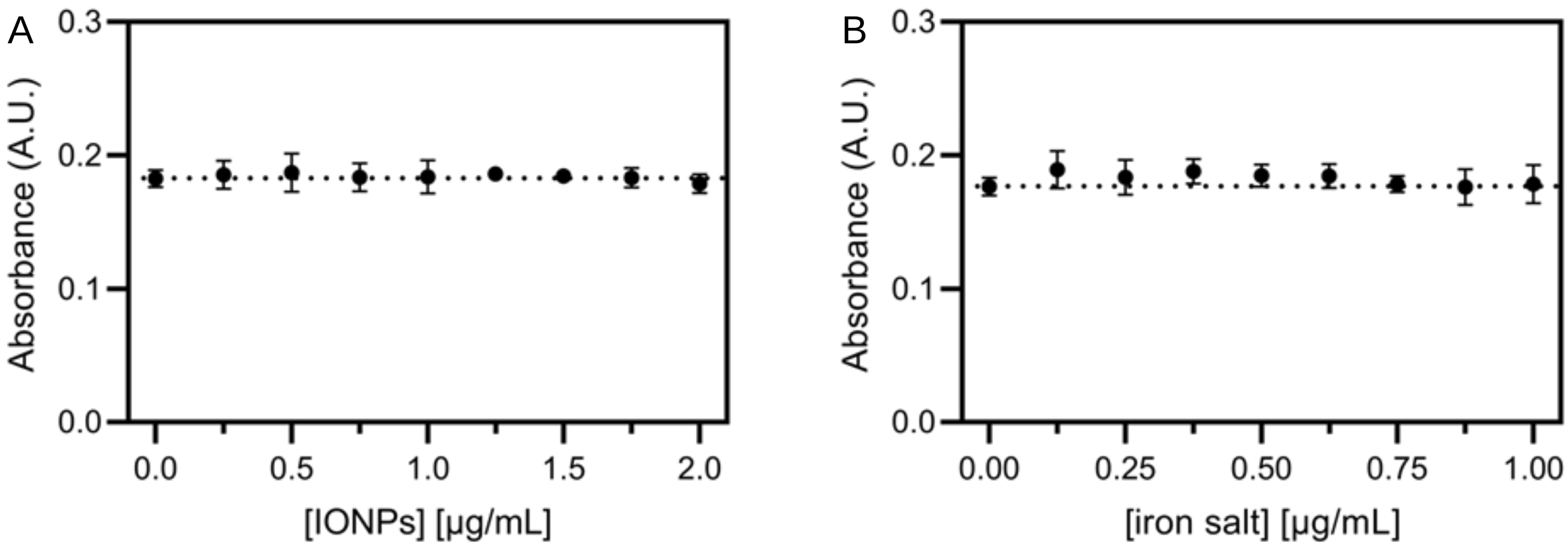

Figure S2. Evaluation of potential colorimetric iron interference on the microBCA protein assay. (**A**) Absorbance of a 20 µg/mL BSA standard spiked with increasing concentrations of IONPs. (**B**) Absorbance of a 20 µg/mL BSA standard spiked with increasing concentrations of iron salt.

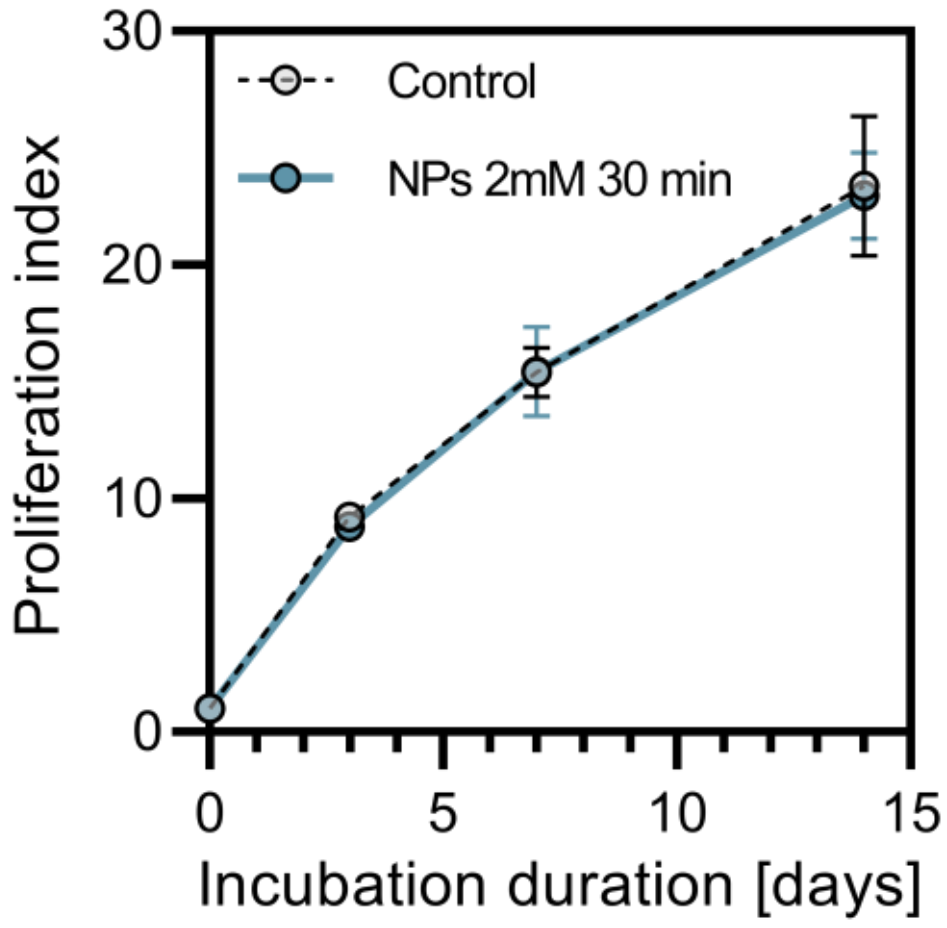

Figure S3. Proliferation index after exposure to 2mM IONPs for 30 minutes (NPs 2mM 30 min), compared to untreated cells (Control). Results are shown as mean ± SD.

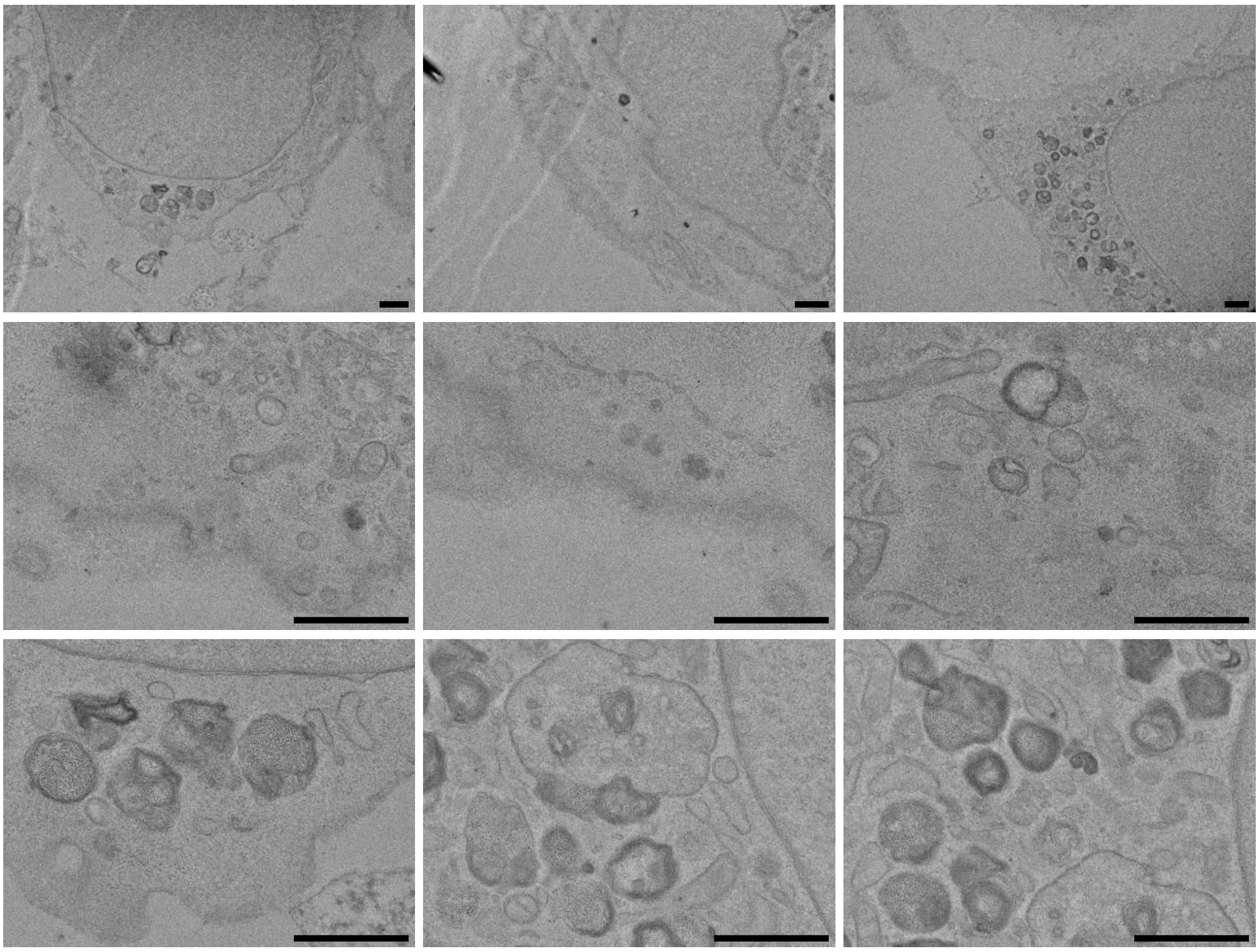

Figure S4. Transmission electron microscopy imaging of mMSCs monolayers incubated with 1 mM quinate for 3 days (Salt 1mM 3d). Scale bars: 1 µm.

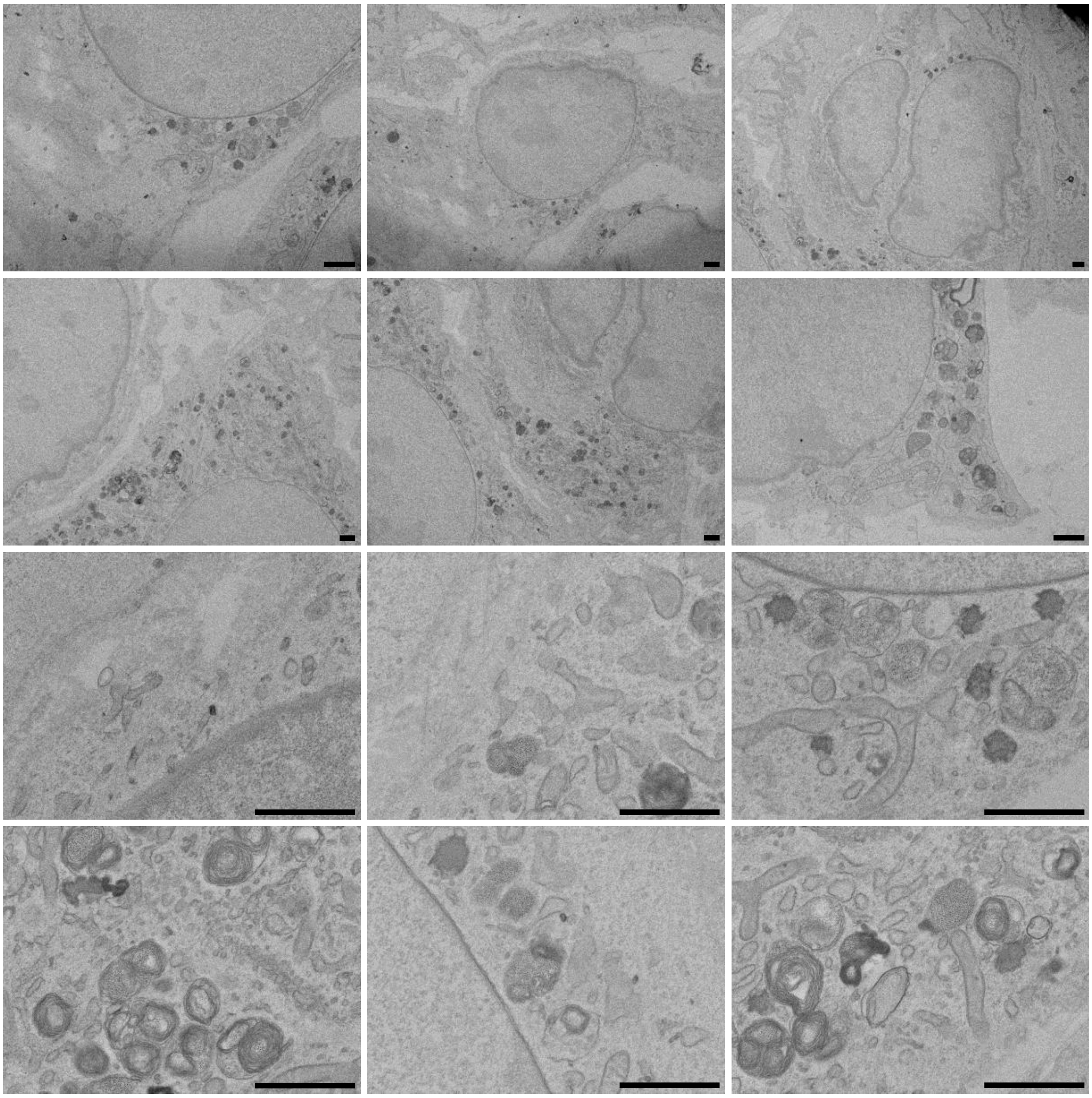

Figure S5. Transmission electron microscopy imaging of mMSCs monolayers incubated with 1 mM quinate for 7 days. Scale bars: 1 µm.

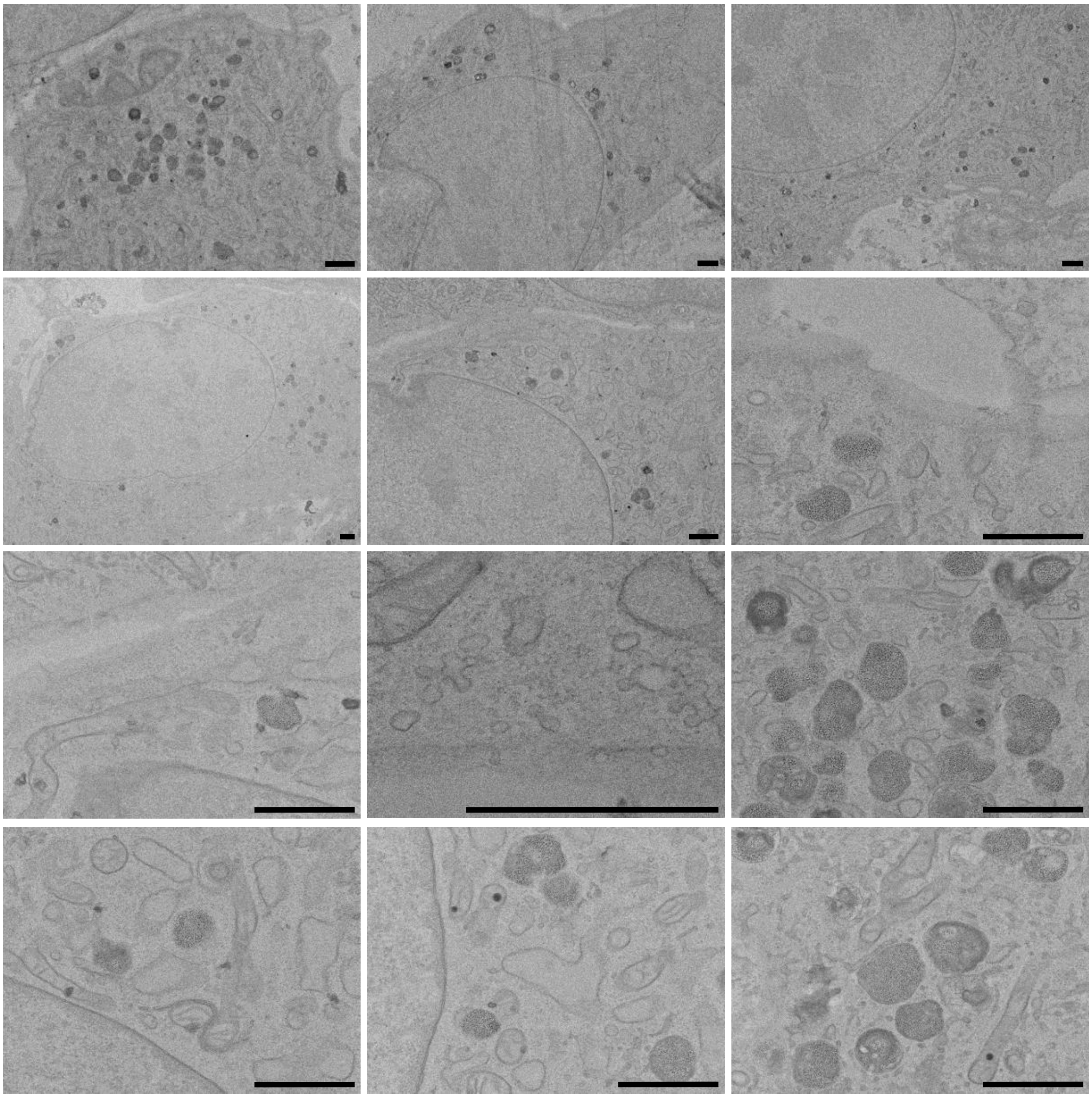

Figure S6. Transmission electron microscopy imaging of mMSCs monolayers incubated with 1 mM quinate for 14 days (Salt 1mM 14d). Scale bars: 1 μm.

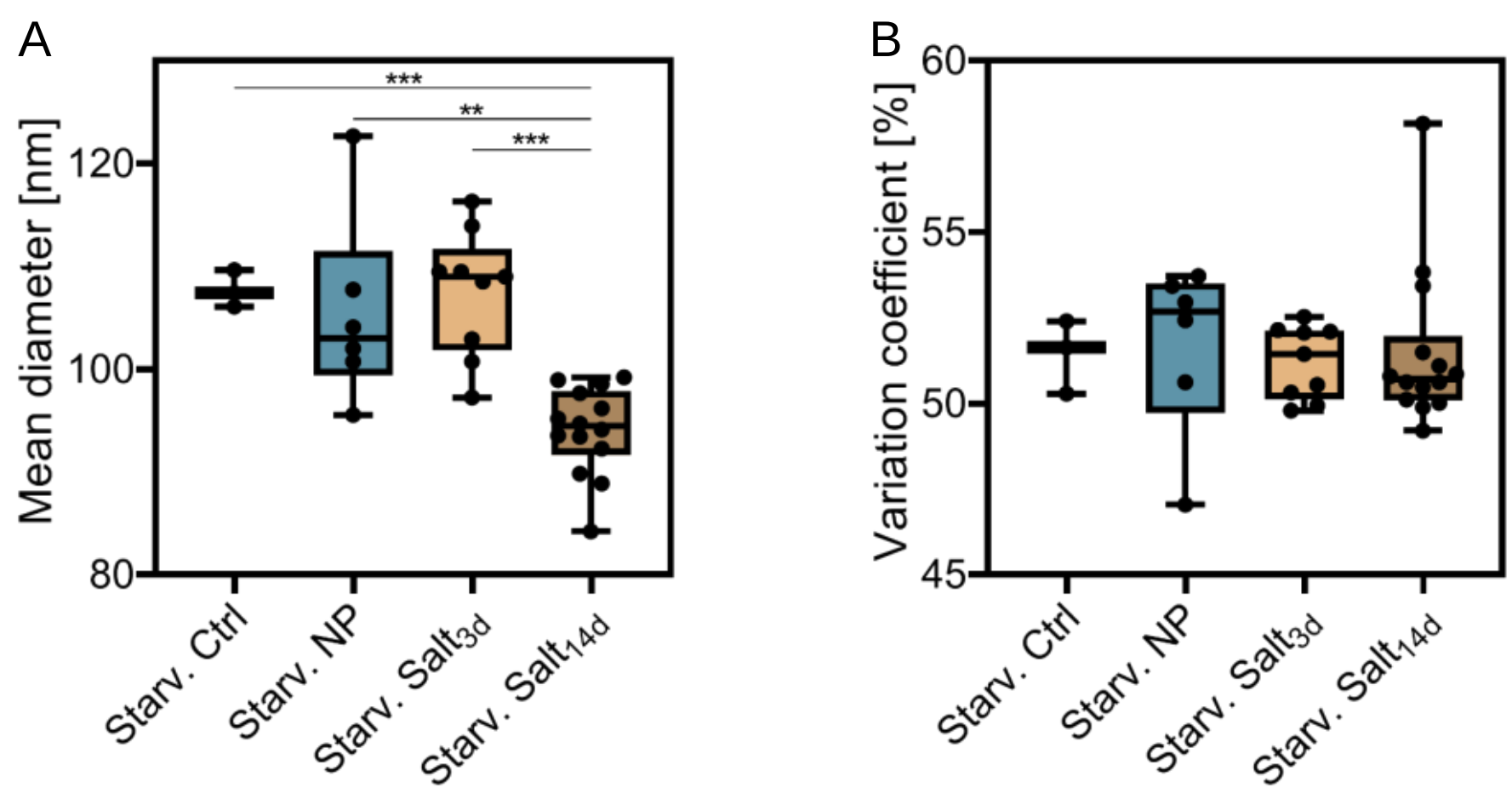

Figure S7. (A) Mean particle diameters in conditioned media collected after starvation-induced secretion, as measured by NTA. (B) Coefficients of variation of particle diameters in conditioned media collected after starvation-induced secretion, as measured by NTA.

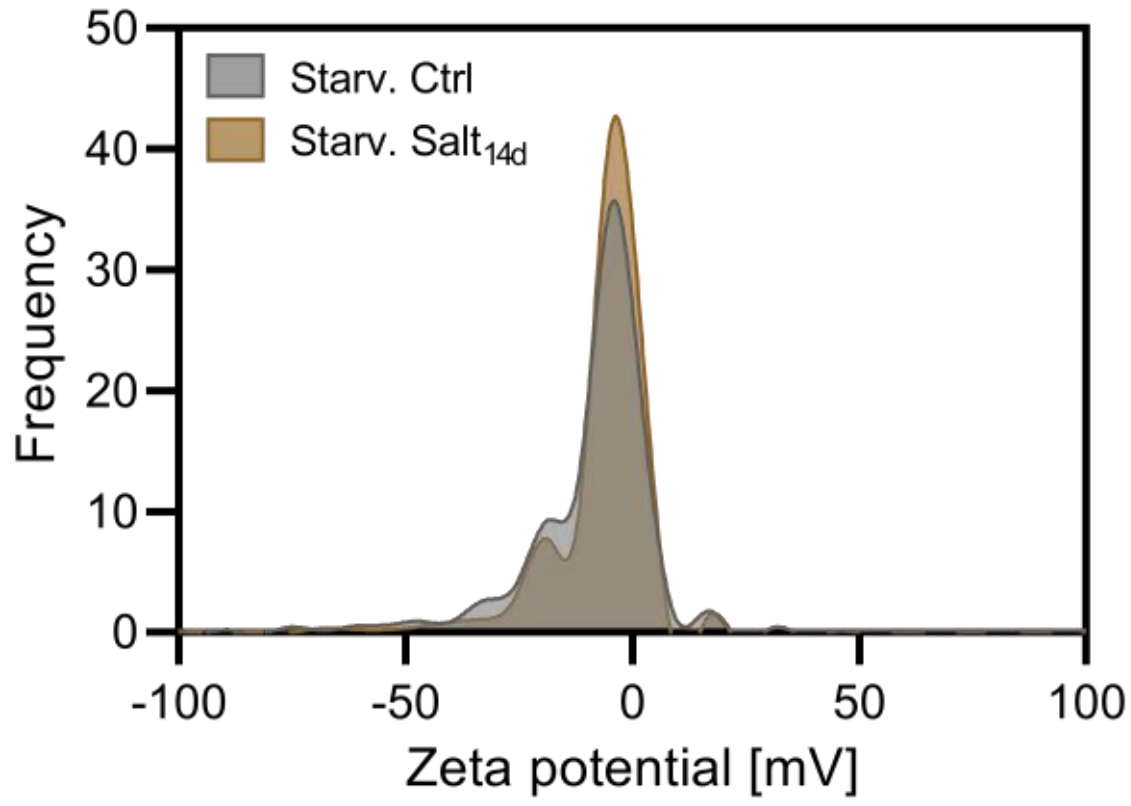

Figure S8. Single-particle zeta potential distributions of extracellular vesicles produced under serum starvation from untreated mMSCs (Starv. Ctrl) or from mMSCs subjected to a 14-day pre-incubation with 1 mM ferric quinate (Starv. $Salt_{14d}$). Profiles were acquired using Tunable Resistive Pulse Sensing (TRPS).

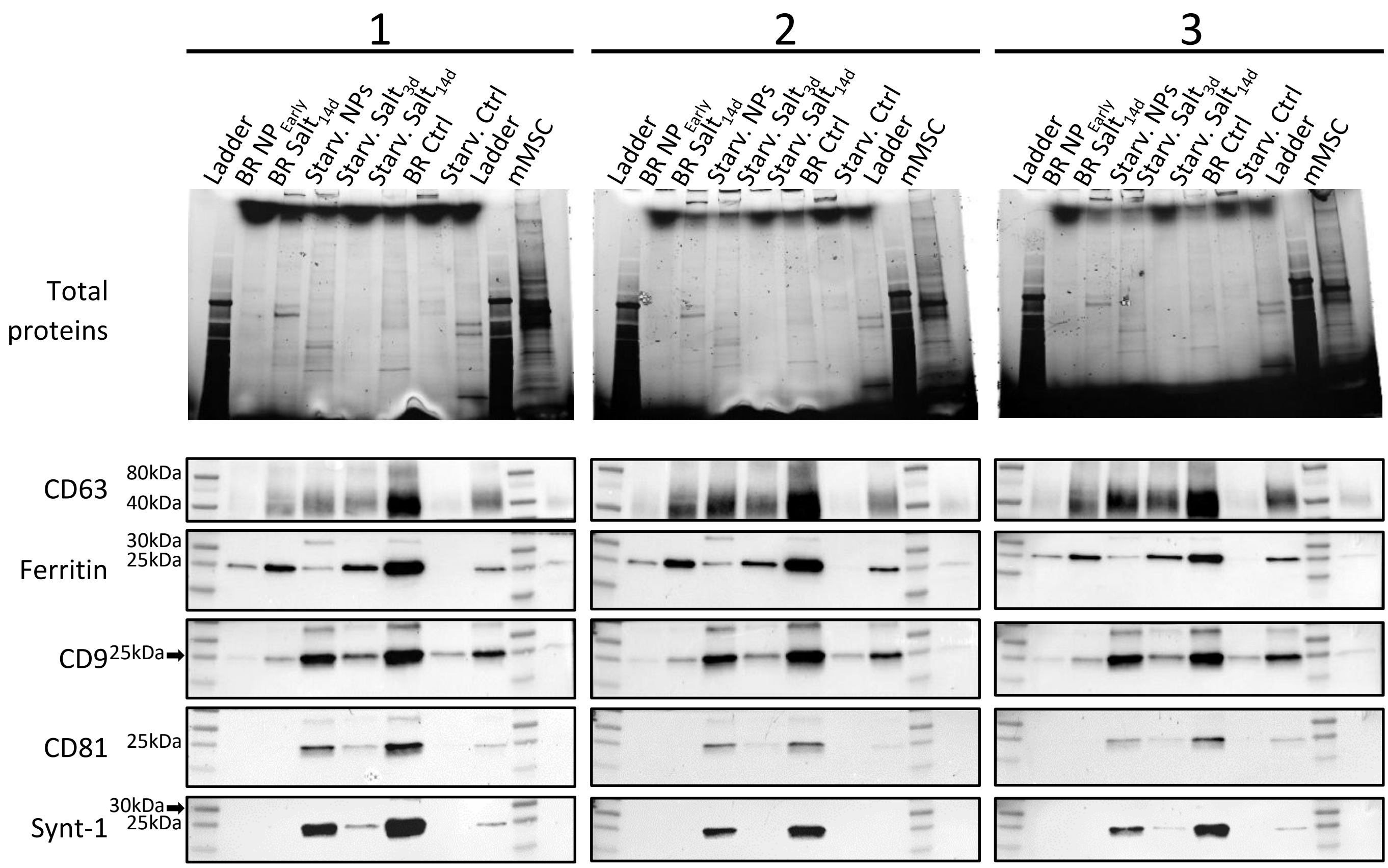

Figure S9. Western blot analysis of EV-specific membrane markers (CD9, CD63, CD81), EV-specific cytosolic marker (Syntenin-1) and cytosolic ferritin in EVs produced from iron-treated mMSCs. The conditions include turbulence stimulation with early-stage IONP incubation (BR NP_{Early}), turbulence stimulation with 14-day ferric quinate incubation (BR $Salt_{14d}$), serum starvation with IONP incubation (Starv. NPs), serum starvation with 3-day ferric quinate incubation (Starv. $Salt_{3d}$), serum starvation with 14-day ferric quinate incubation (Starv. $Salt_{14d}$), turbulence stimulation control (BR Ctrl) and serum starvation control (Starv. Ctrl). Whole mMSC lysates (mMSC) are included. CD81 and Syntenin-1 bands were not detected; the visible bands correspond to CD9.

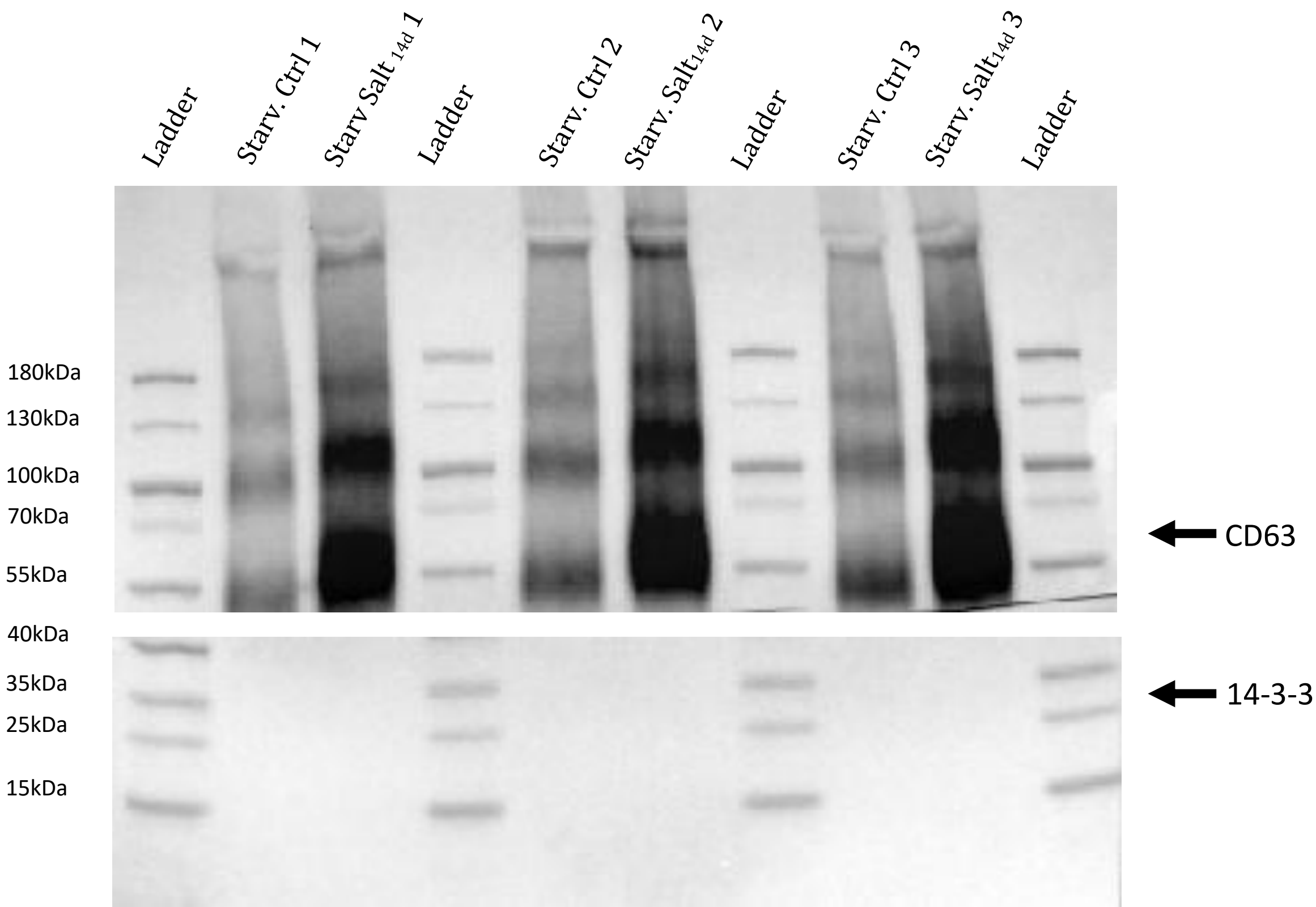

Figure S10. Western blot analysis of membrane EV-marker CD63 and non-EV negative marker 14-3-3 in EVs produced from iron-treated mMSCs via serum starvation. The conditions include serum starvation control (Starv. Ctrl) and serum starvation with 14-day ferric quinate incubation (Starv. $Salt_{14d}$).

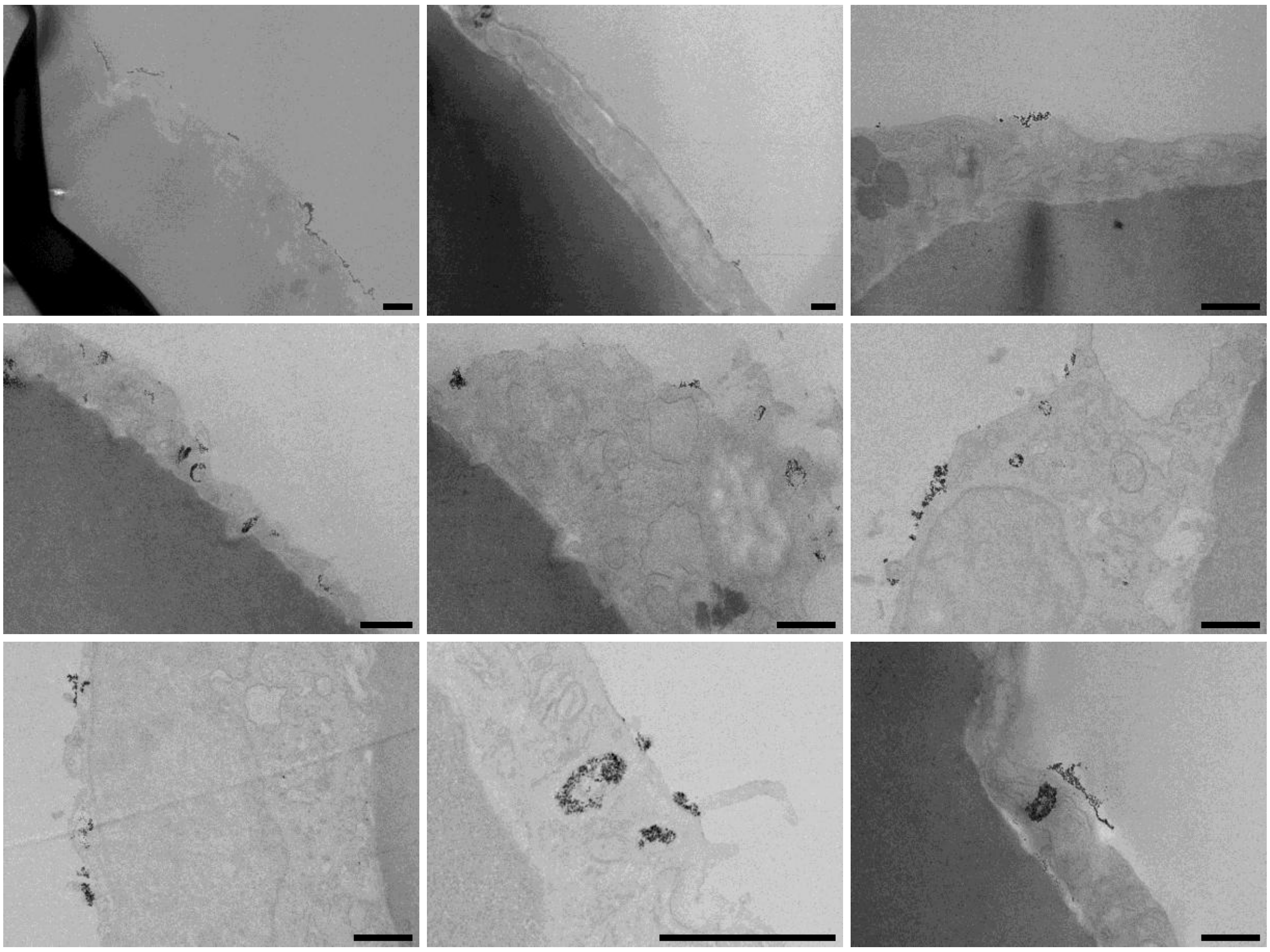

Figure S11. Transmission electron microscopy imaging of mMSCs cultured on microbeads incubated with 2 mM iron oxide nanoparticles for 30 minutes (BR NP_{Early}). Scale bars: 1 µm.

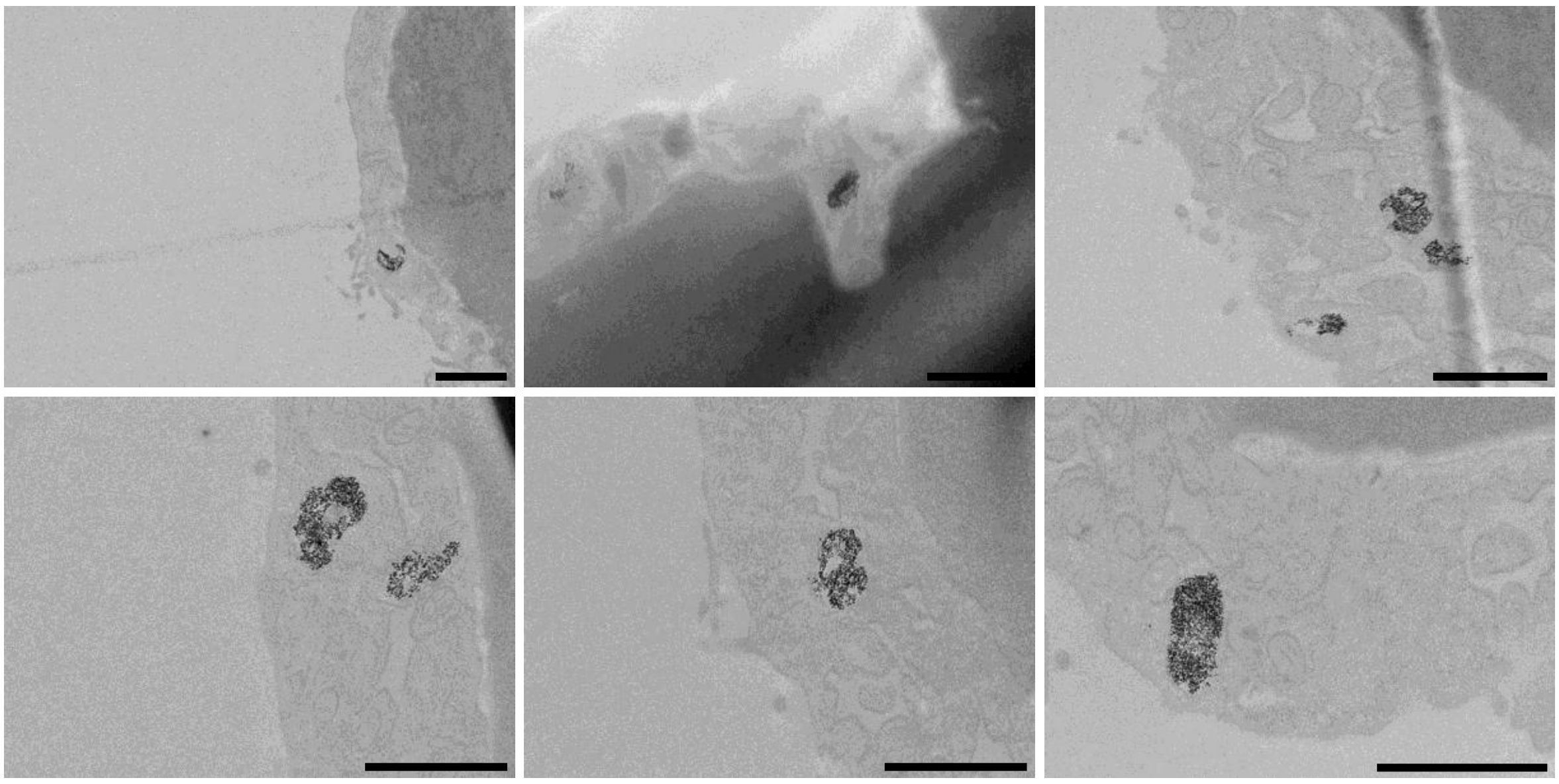

Figure S12. Transmission electron microscopy imaging of mMSCs cultured on microbeads incubated with 2 mM IONP for 30 minutes followed by 2 hours of chase (BR NP_{Late}). Scale bars: 1 µm.

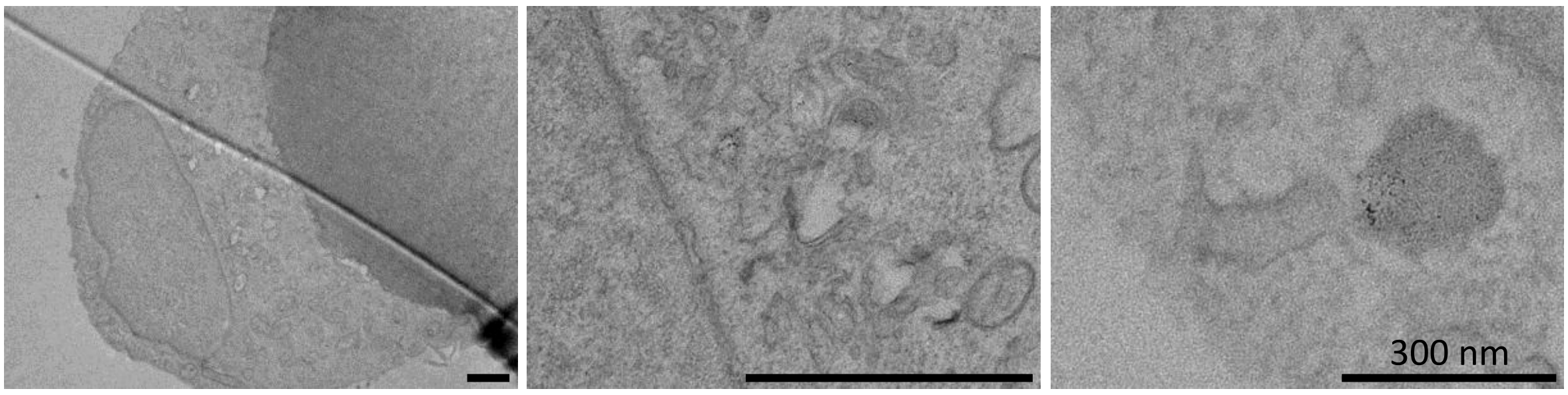

Figure S13. Transmission electron microscopy imaging of mMSCs cultured on microbeads incubated with 1 mM quinate for 14 days (BR $Salt_{14d}$). Scale bars: 1 µm (unless otherwise indicated).

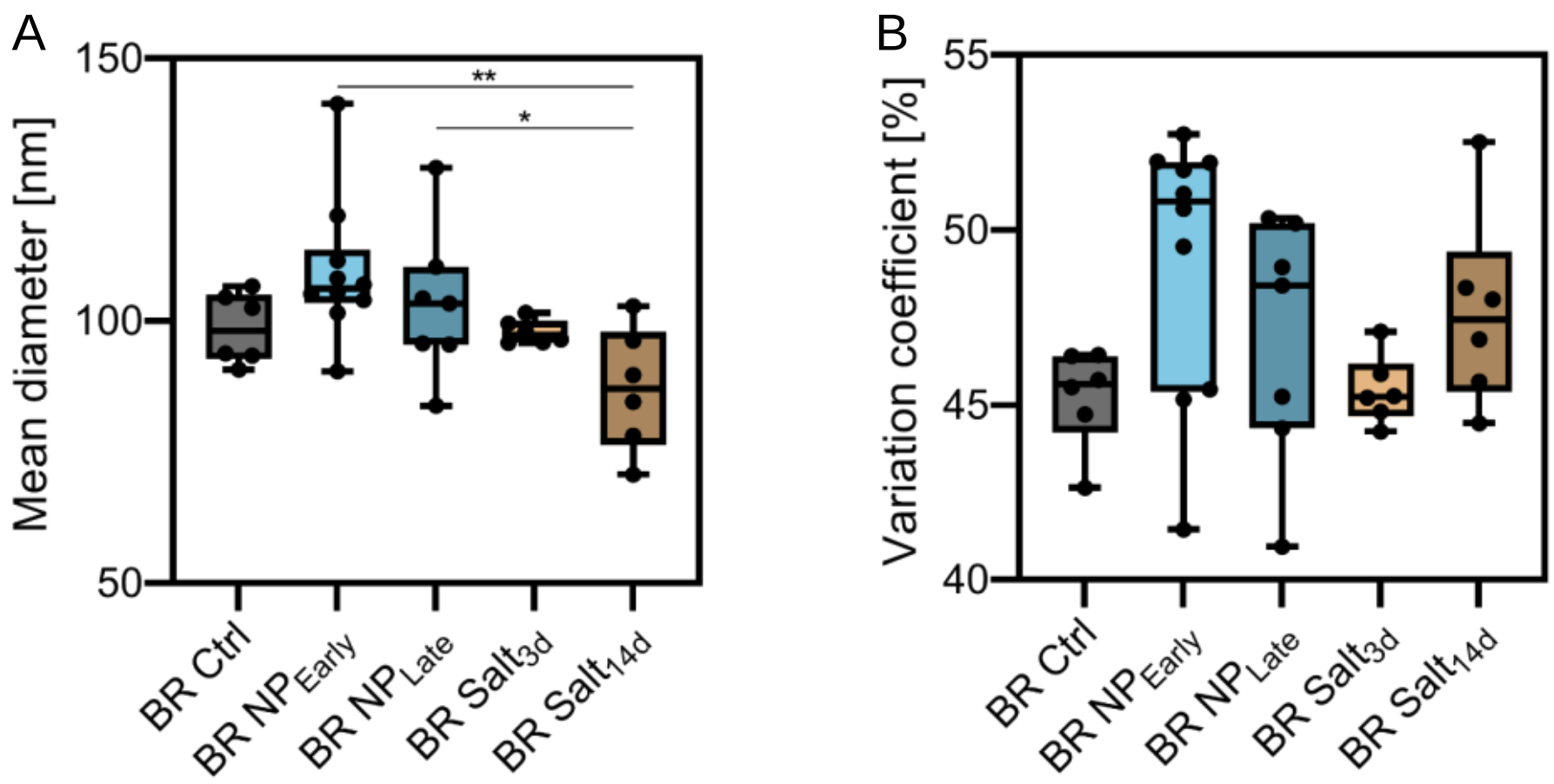

Figure S14. (**A**) Mean particle diameters in conditioned media collected after turbulence-induced secretion, as measured by NTA. (**B**) Coefficients of variation of particle diameters in conditioned media collected after turbulence-induced secretion, as measured by NTA.

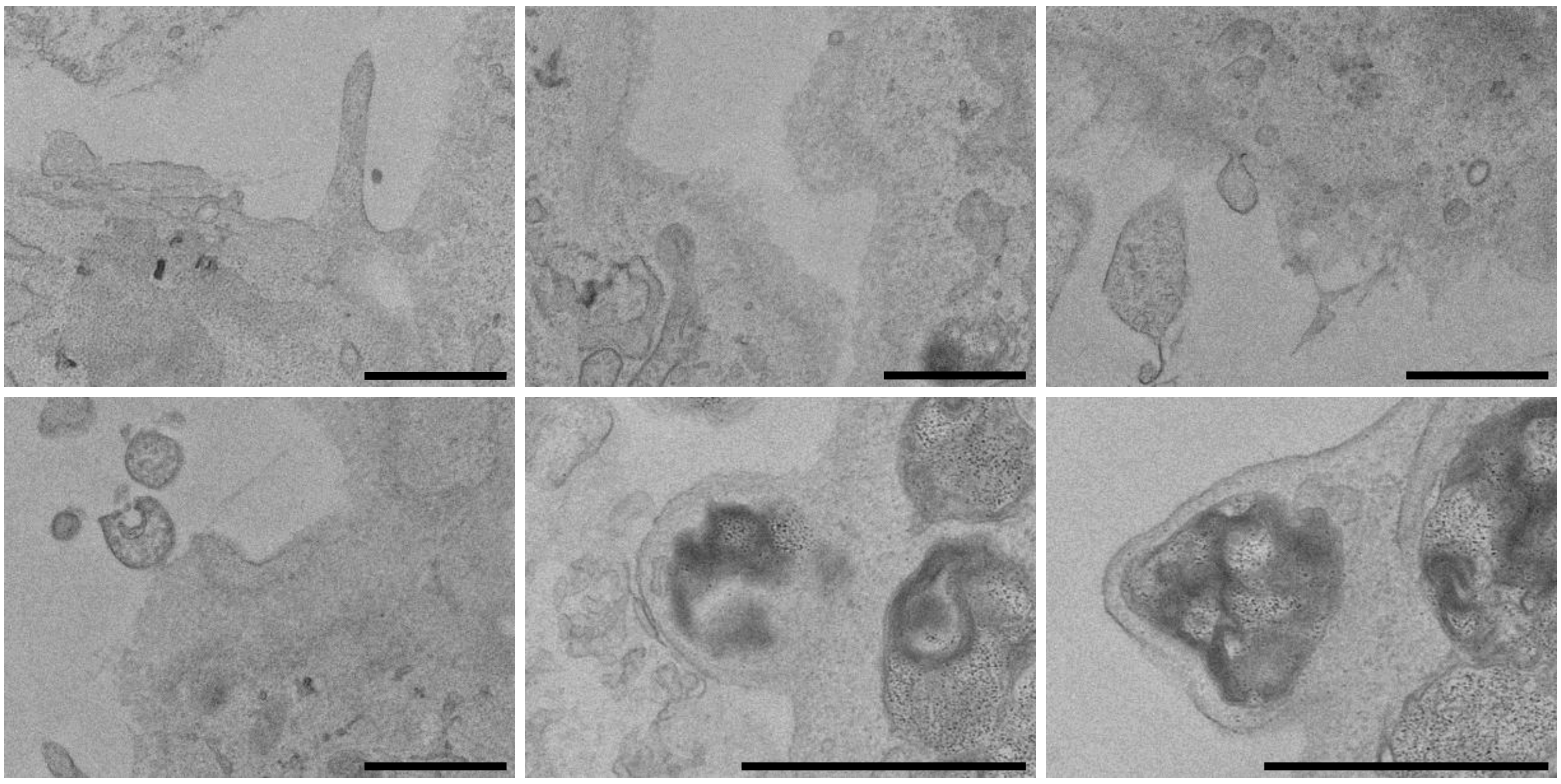

Figure S15. Transmission electron microscopy imagine depicting the biogenesis of ferritine-loaded EVs from monolayers mMSCs incubated with 1 mM quinate for 3 days. Scale bars: 1 µm.

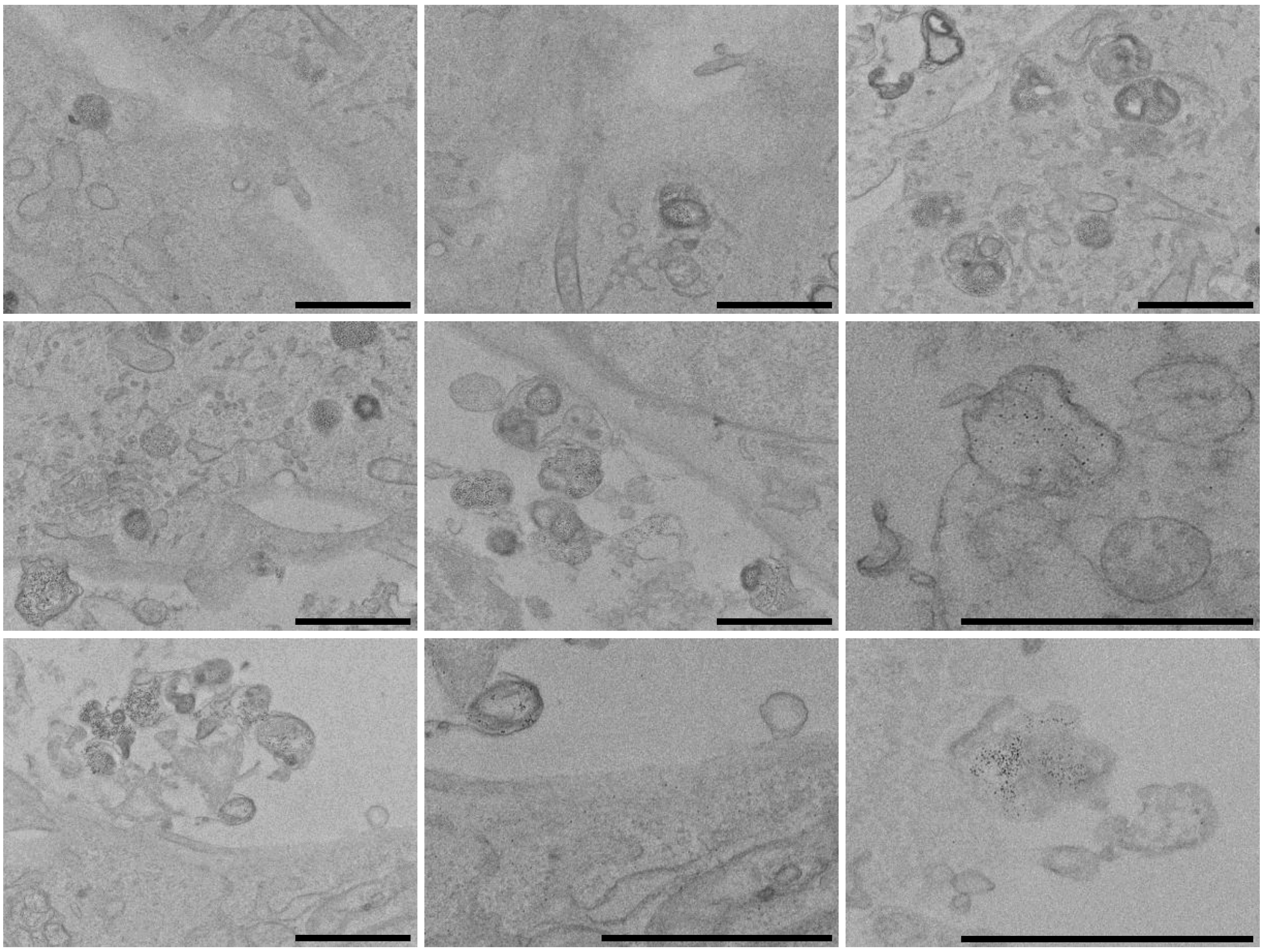

Figure S16. Transmission electron microscopy imaging depicting the biogenesis of ferritine-loaded EVs from monolayers mMSCs incubated with 1 mM quinate for 14 days (Starv. $Salt_{14d}$). Scale bars: 1 µm.

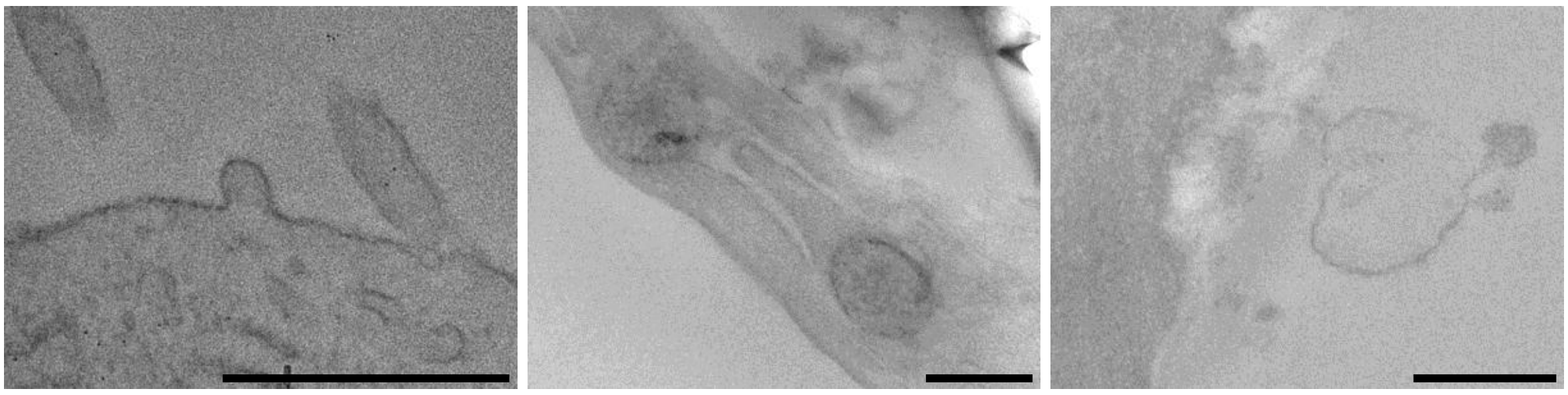

Figure S17. Transmission electron microscopy imaging depicting the biogenesis of ferritine-loaded EVs from mMSCs cultured on microbeads incubated with 1 mM quinate for 14 days (BR $Salt_{14d}$). Scale bars: 500 nm.

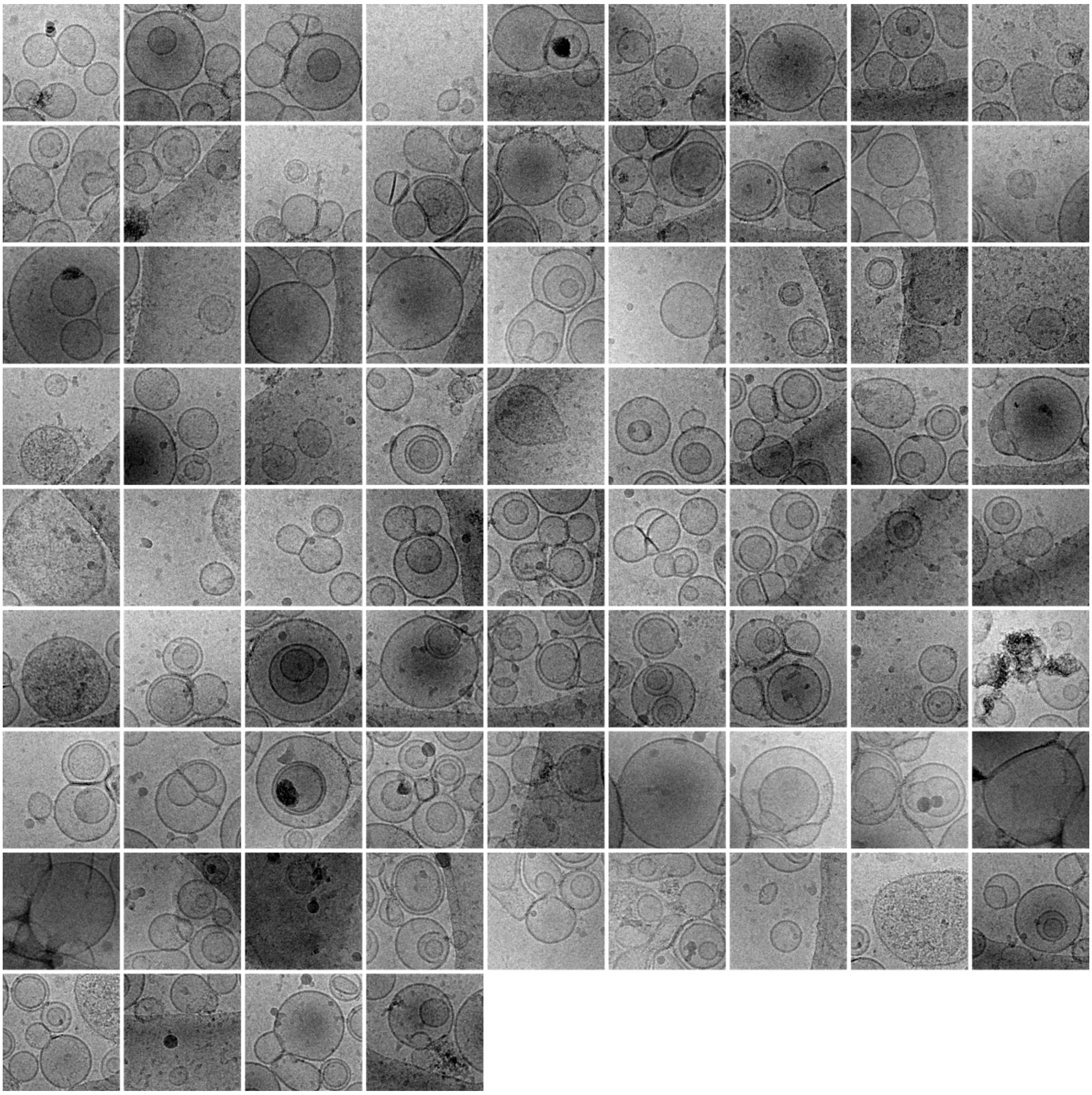

Figure S18. Cryogenic transmission electron microscopy (cryoTEM) of extracellular vesicles (EVs) produced through turbulence stimulation from control cells (BR Ctrl). Each image has a scale of 300 nm per side.

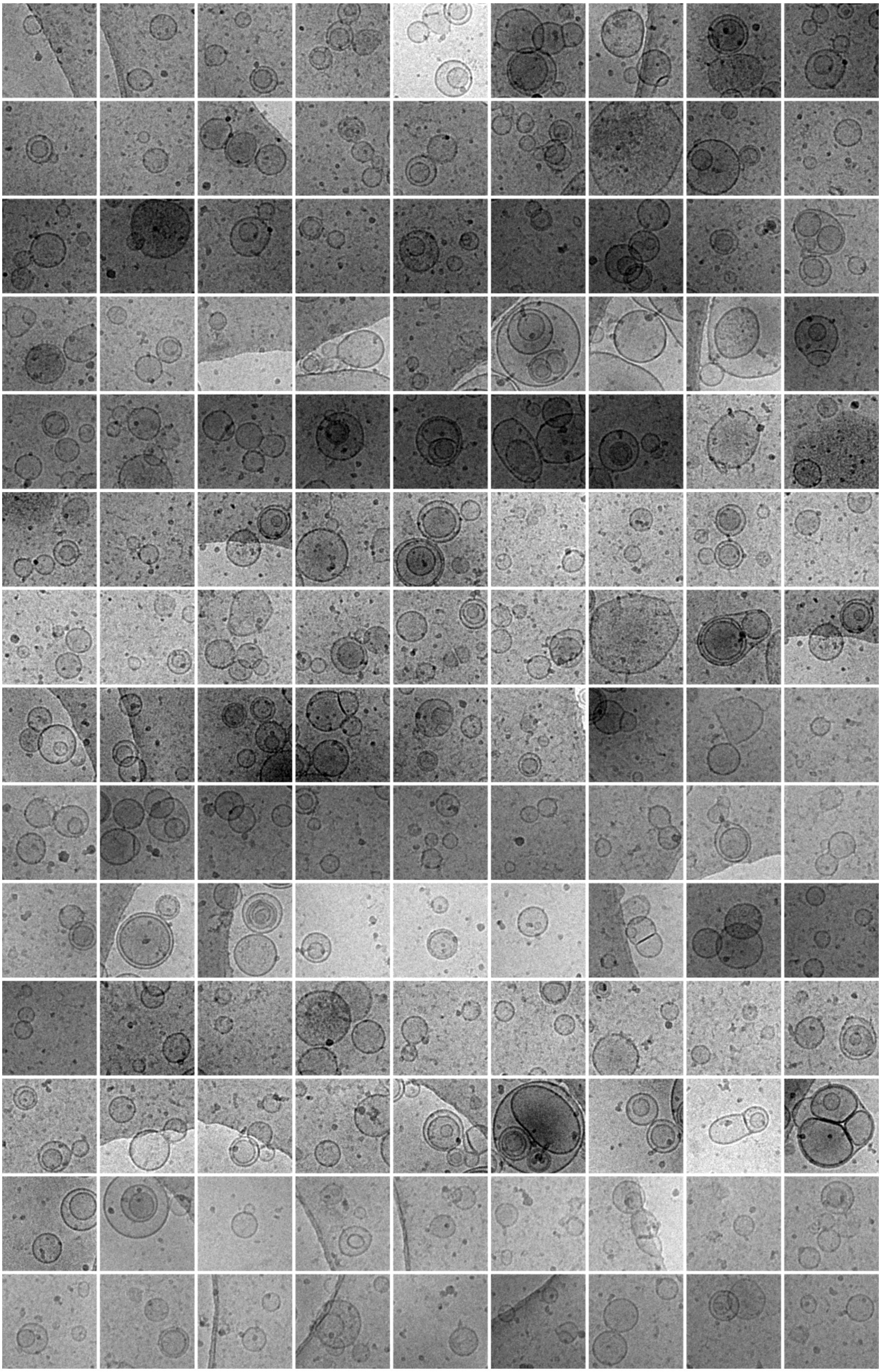

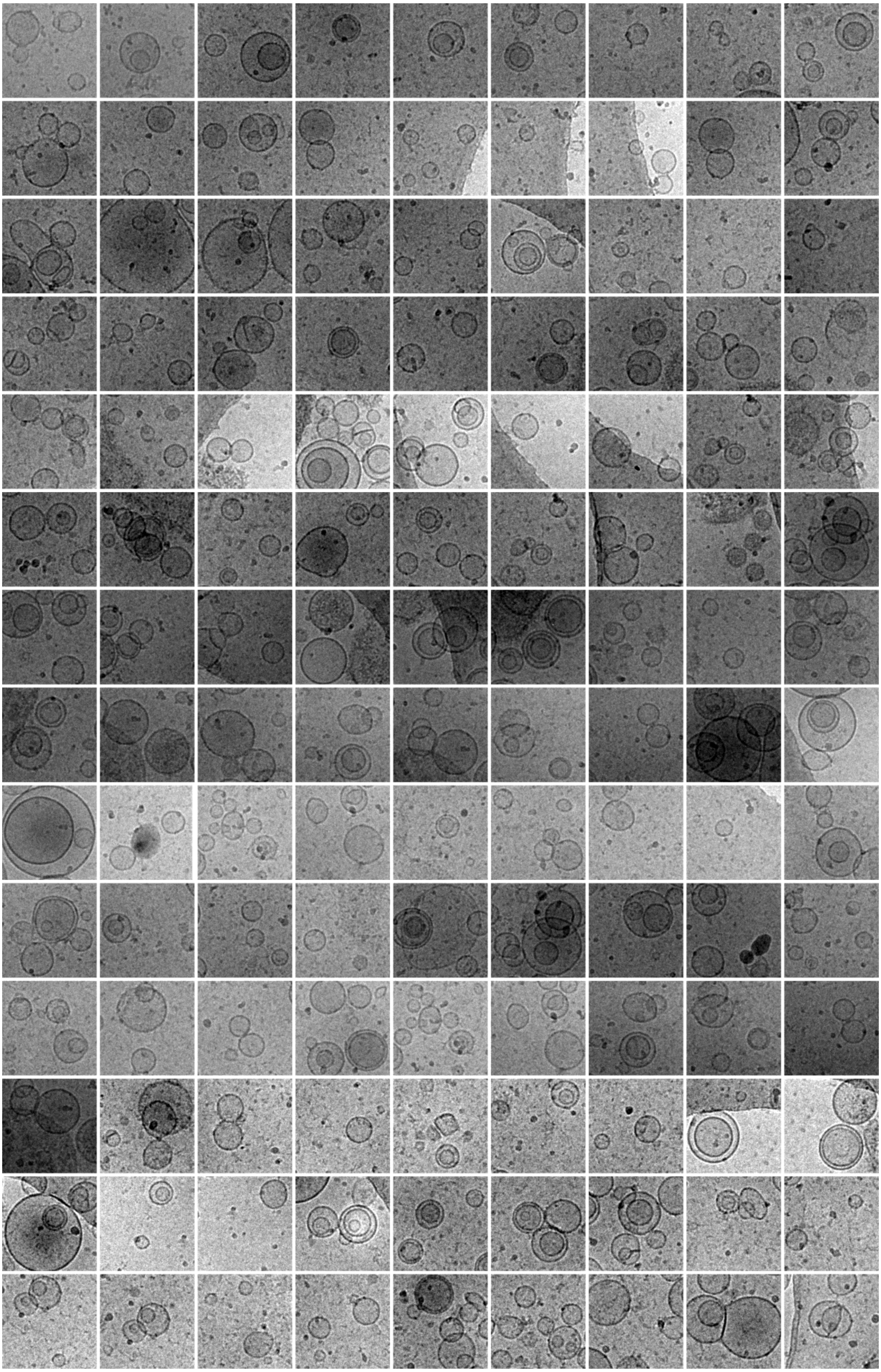

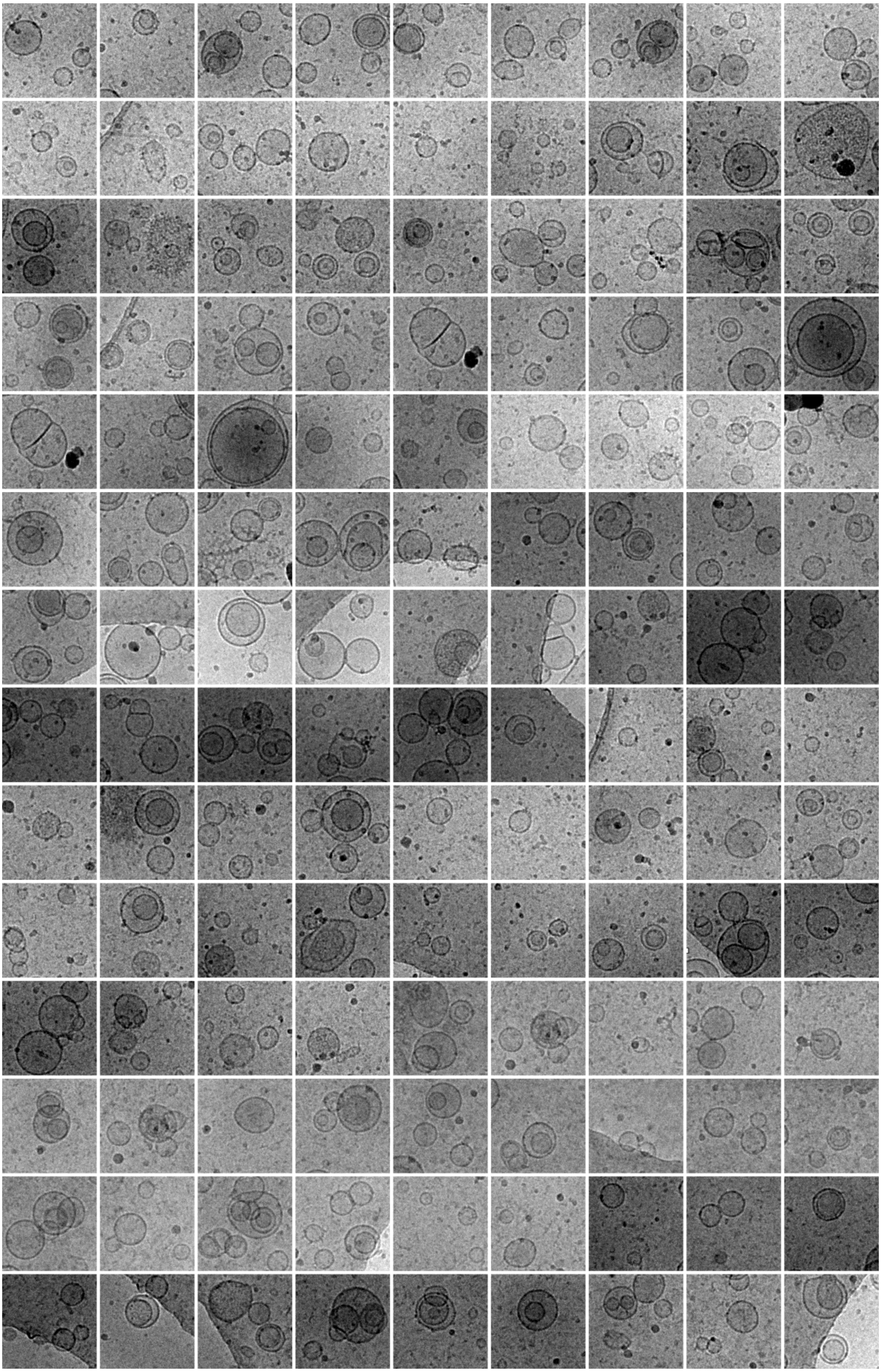

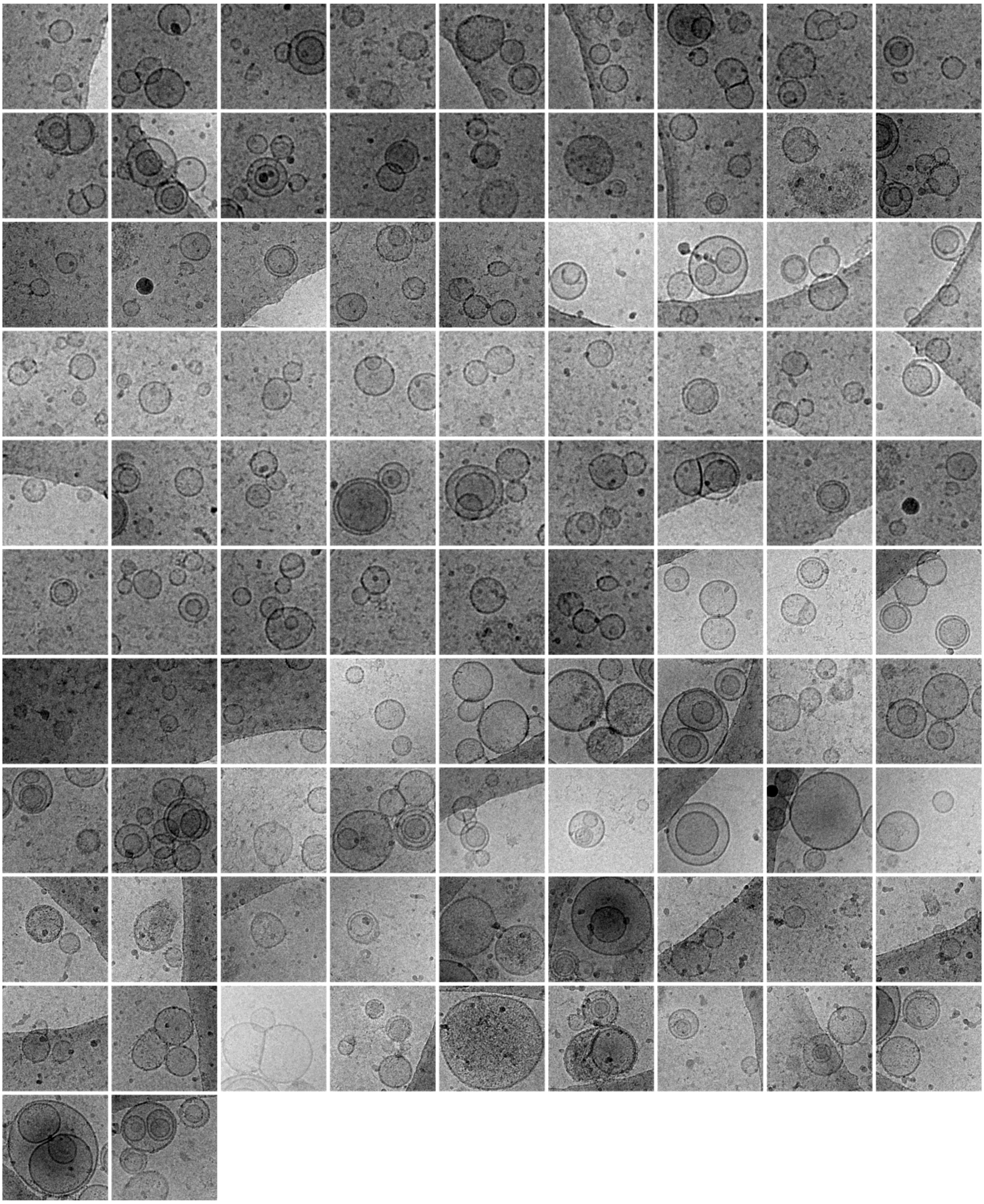

Figure S19. Cryogenic transmission electron microscopy (cryoTEM) of extracellular vesicles (EVs) produced through turbulence stimulation from early-stage IONP-treated cells (BR NP_{Early}). Each image has a scale of 300 nm per side.

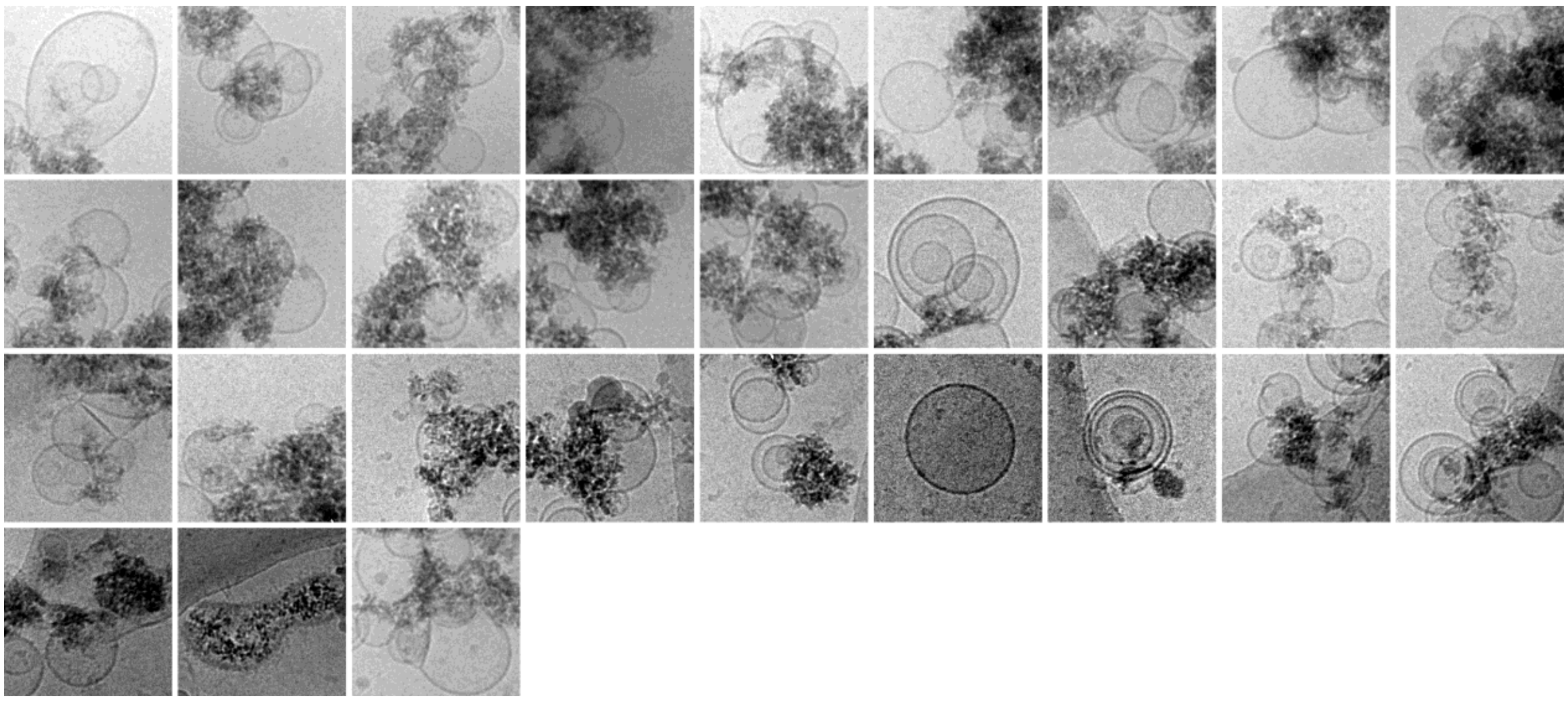

Figure S20. Cryogenic transmission electron microscopy (cryoTEM) of extracellular vesicles (EVs) produced through turbulence stimulation from late-stage IONP-treated cells (BR NP_{Late}). Each image has a scale of 300 nm per side.

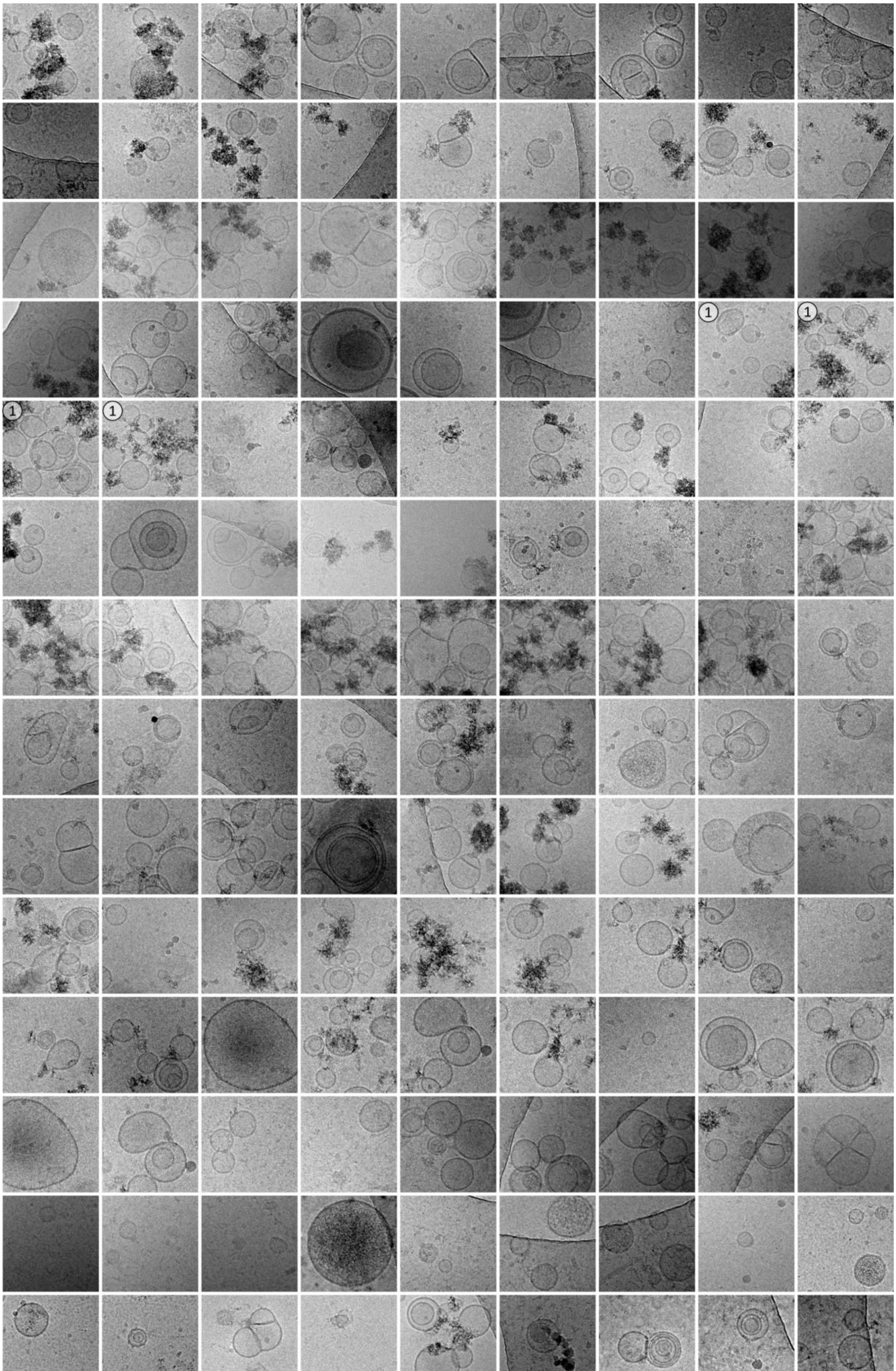
1
1
1
1
1

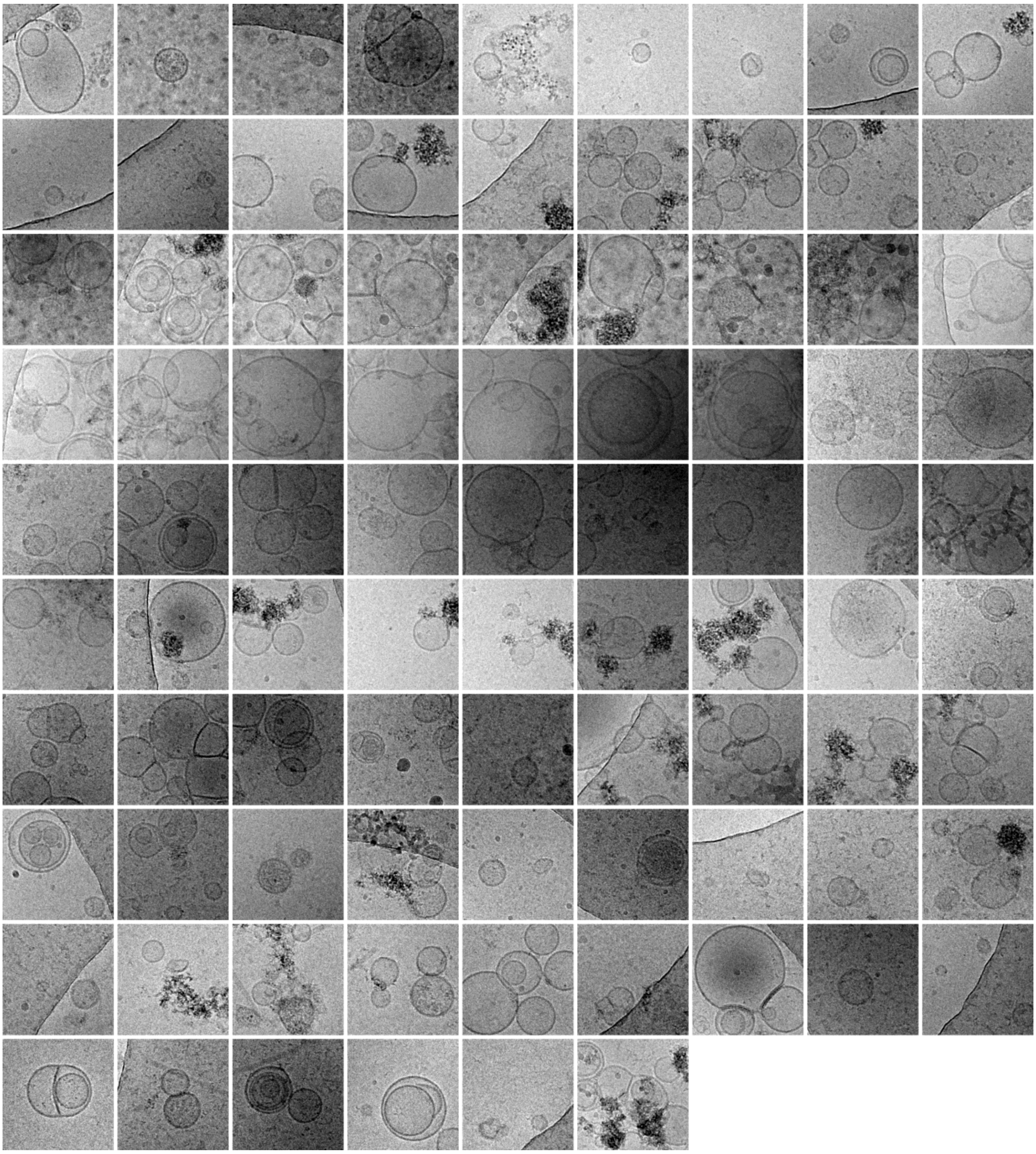

Figure S21. Cryogenic transmission electron microscopy (cryoTEM) of extracellular vesicles (EVs) produced through turbulence stimulation from short-term ferric quinate-treated cells (BR $Salt_{3d}$). Each image has a scale of 300 nm per side. Circled numbers correspond to the respective X-ray energy dispersive spectroscopy (XEDS) spectrum for the indicated areas.

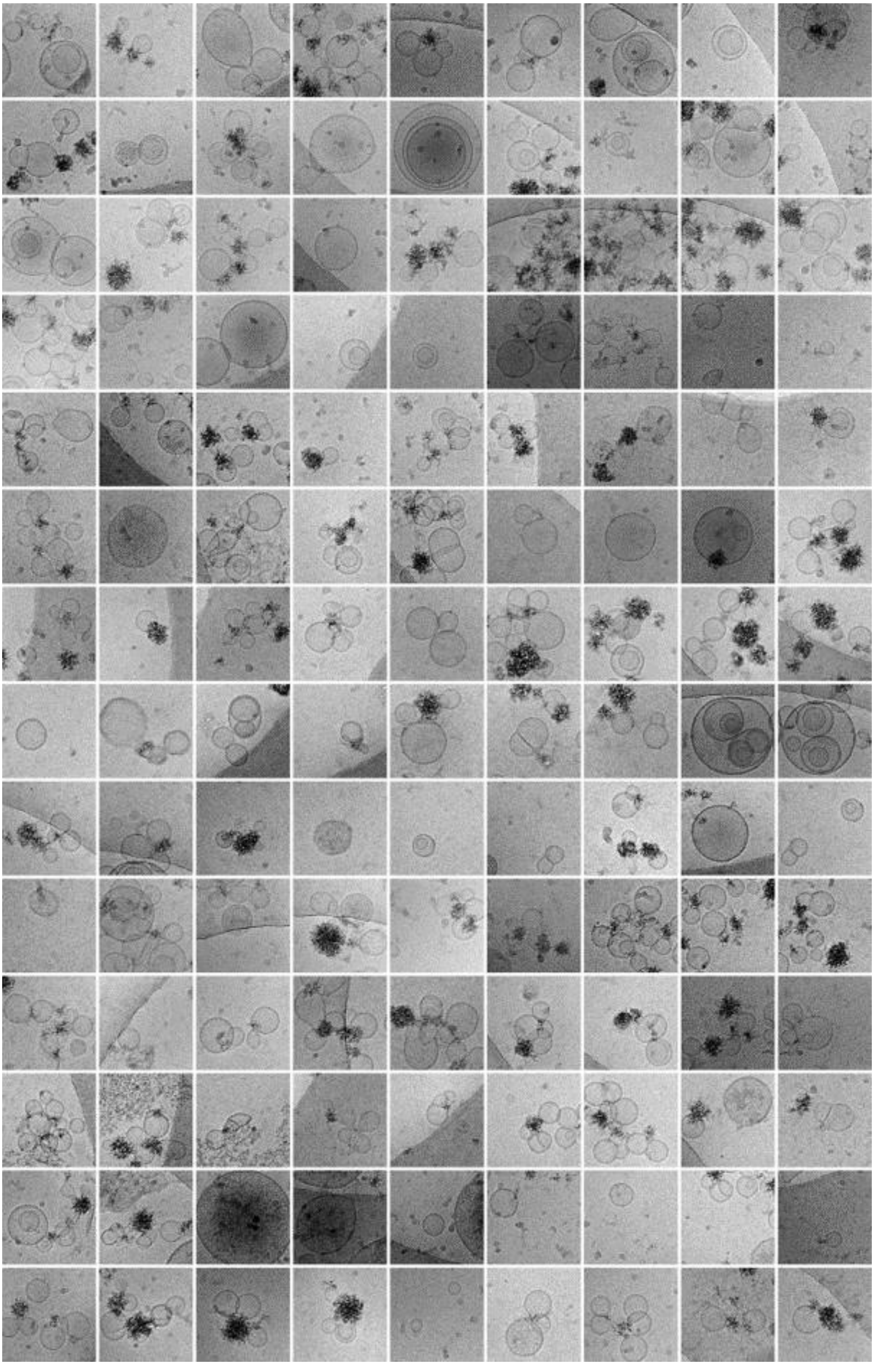

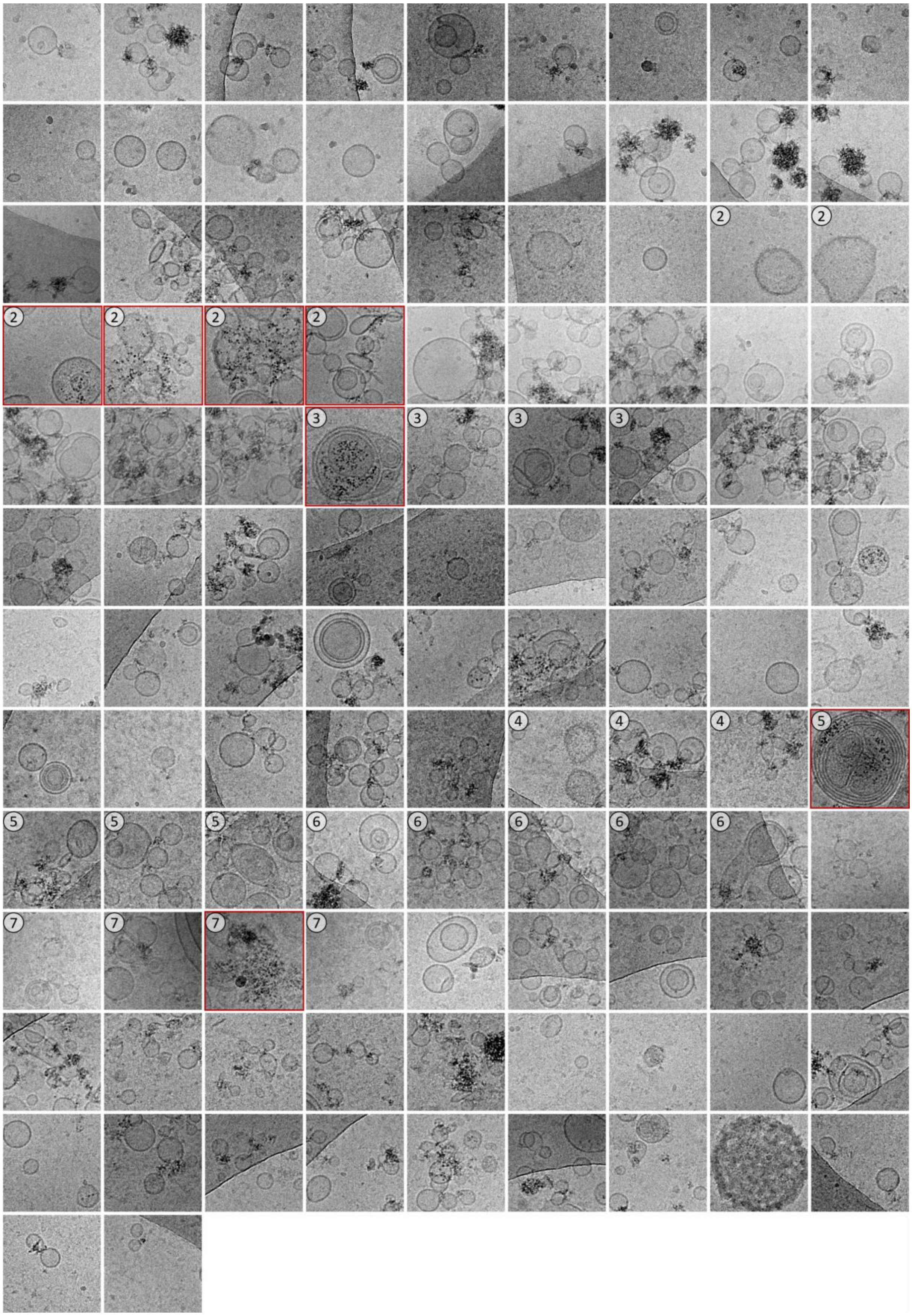

Figure S22. Cryogenic transmission electron microscopy (cryoTEM) of extracellular vesicles (EVs) produced through turbulence stimulation from prolonged ferric quinate-treated cells (BR $Salt_{14d}$). Each image has a scale of 300 nm per side. Circled numbers correspond to the respective X-ray energy dispersive spectroscopy (XEDS) spectrum for the indicated areas. Images exhibiting iron material are framed in red.

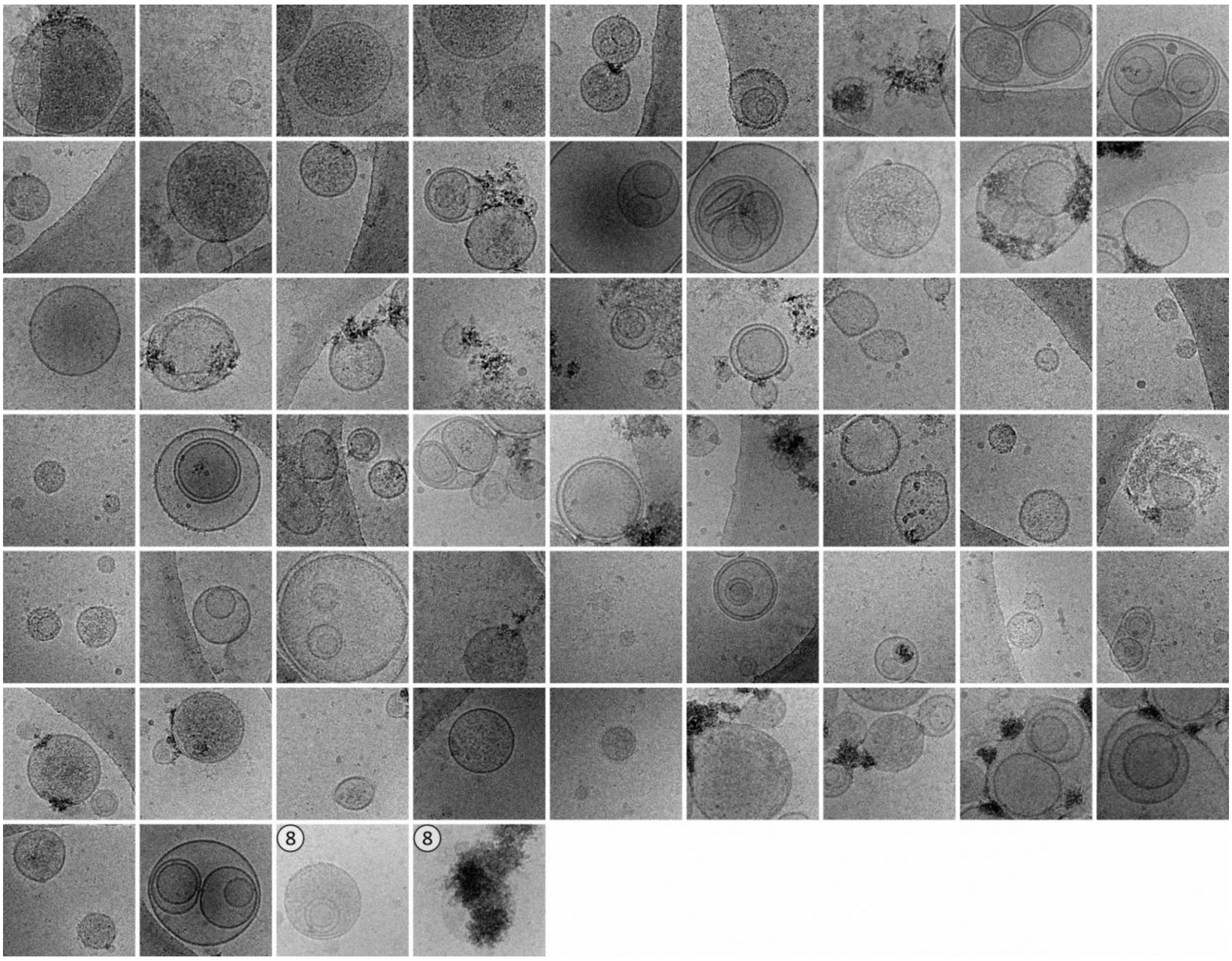

Figure S23. Cryogenic transmission electron microscopy (cryoTEM) of extracellular vesicles (EVs) produced through serum starvation from control cells (Starv. Ctrl). Each image has a scale of 300 nm per side.

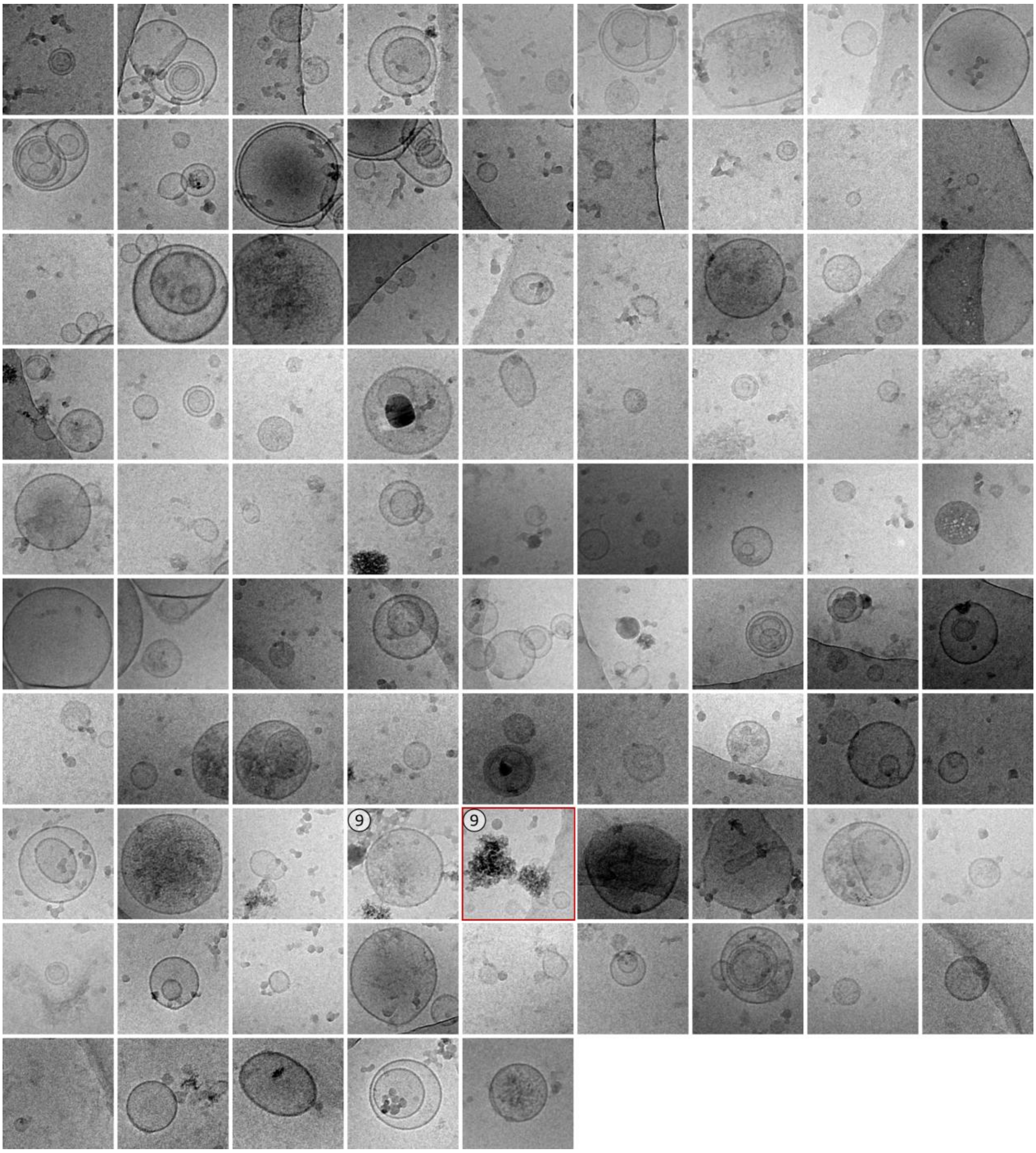

Figure S24. Cryogenic transmission electron microscopy (cryoTEM) of extracellular vesicles (EVs) produced through serum starvation from IONP-treated cells (Starv. NPs). Each image has a scale of 300 nm per side. Circled numbers correspond to the respective X-ray energy dispersive spectroscopy (XEDS) spectrum for the indicated areas. Images exhibiting iron material are framed in red.

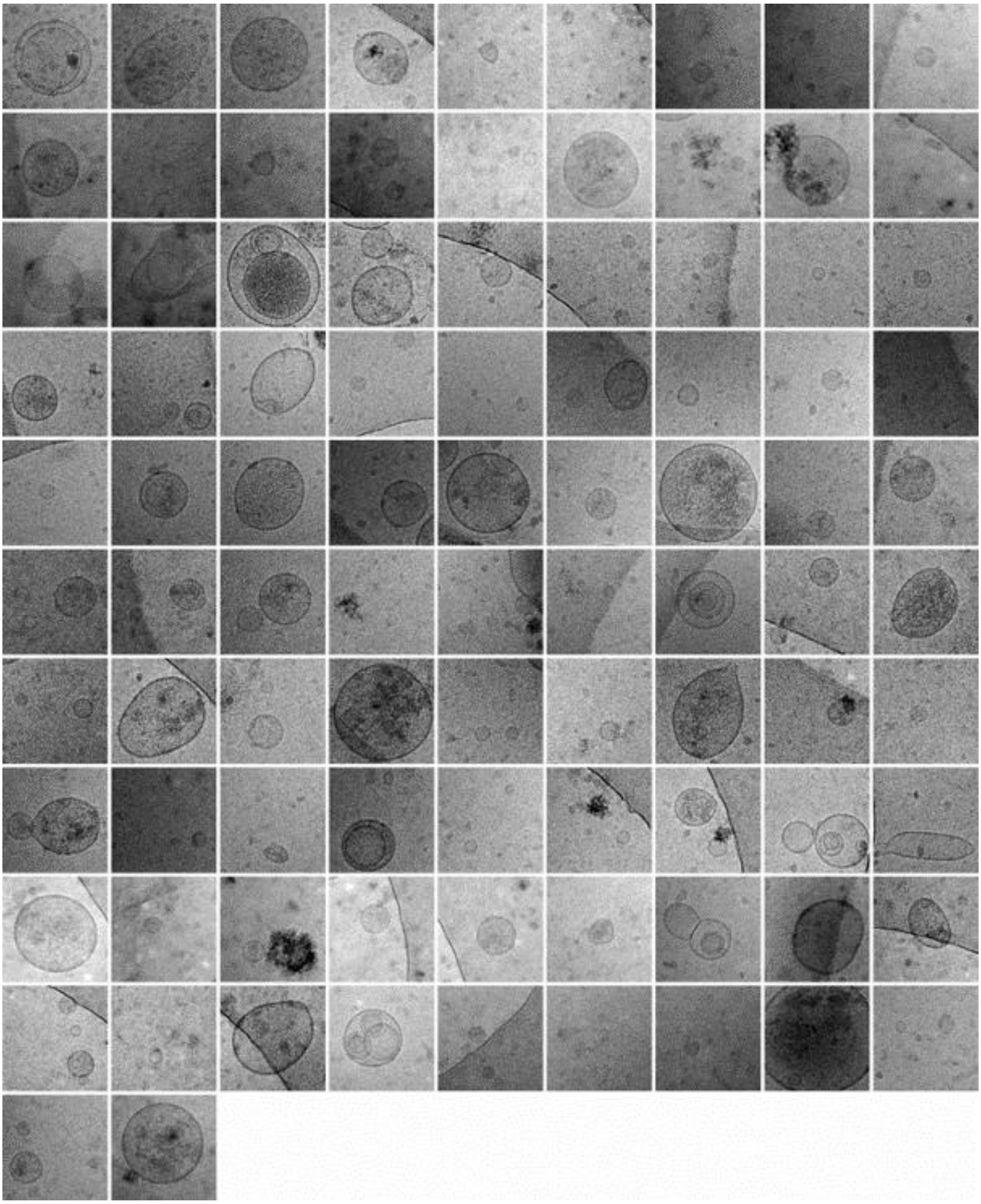

Figure S25. Cryogenic transmission electron microscopy (cryoTEM) of extracellular vesicles (EVs) produced through serum starvation from short-term ferric quinate-treated cells (Starv. $Salt_{3d}$). Each image has a scale of 300 nm per side.

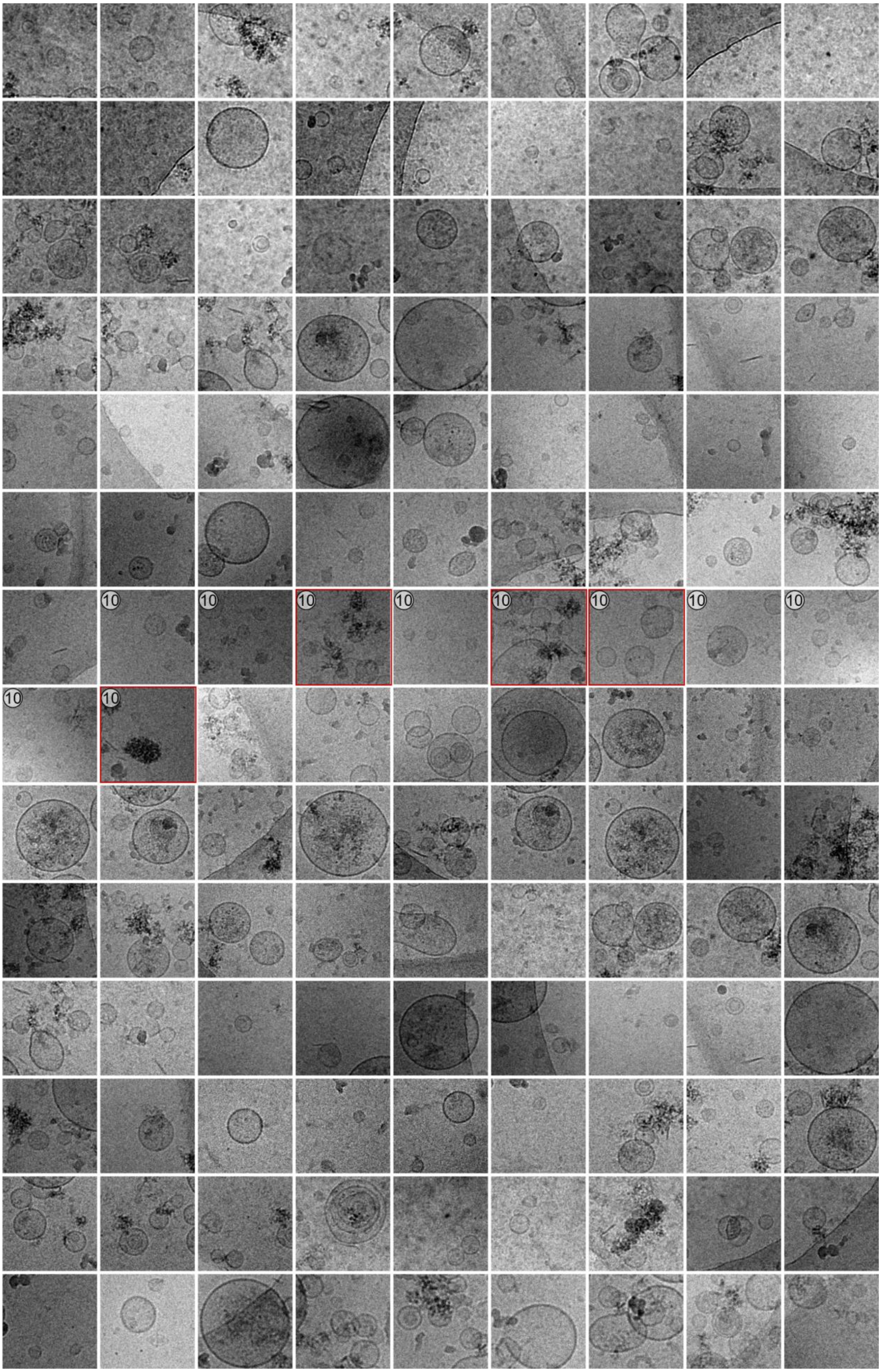
10
10
10
10
10
10
10
10
10
10

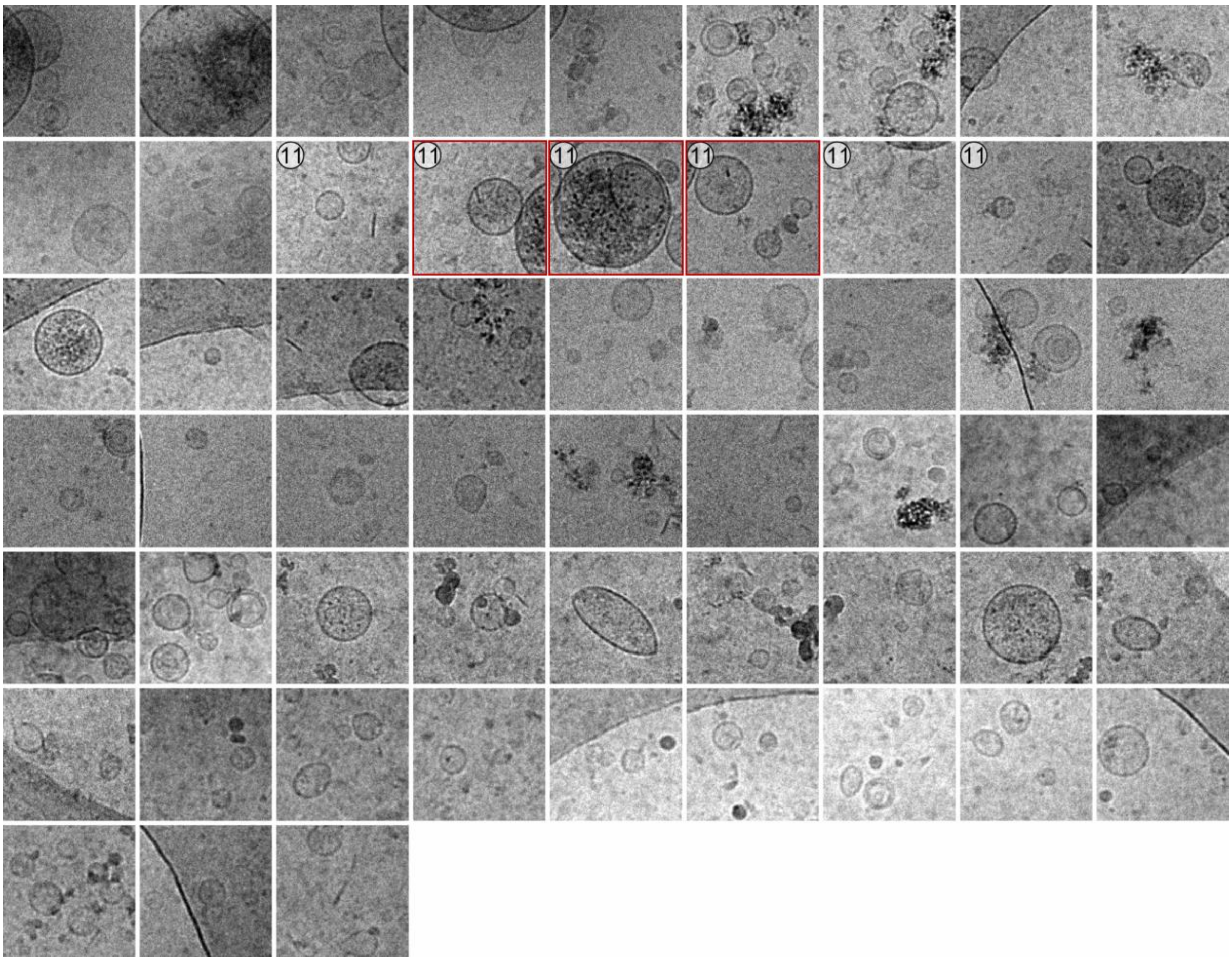

Figure S26. Cryogenic transmission electron microscopy (cryoTEM) of extracellular vesicles (EVs) produced through serum starvation from prolonged ferric quinate-treated cells (Starv. $Salt_{14d}$). Each image has a scale of 300 nm per side. Circled numbers correspond to the respective X-ray energy dispersive spectroscopy (XEDS) spectrum for the indicated areas. Images exhibiting iron material are framed in red.

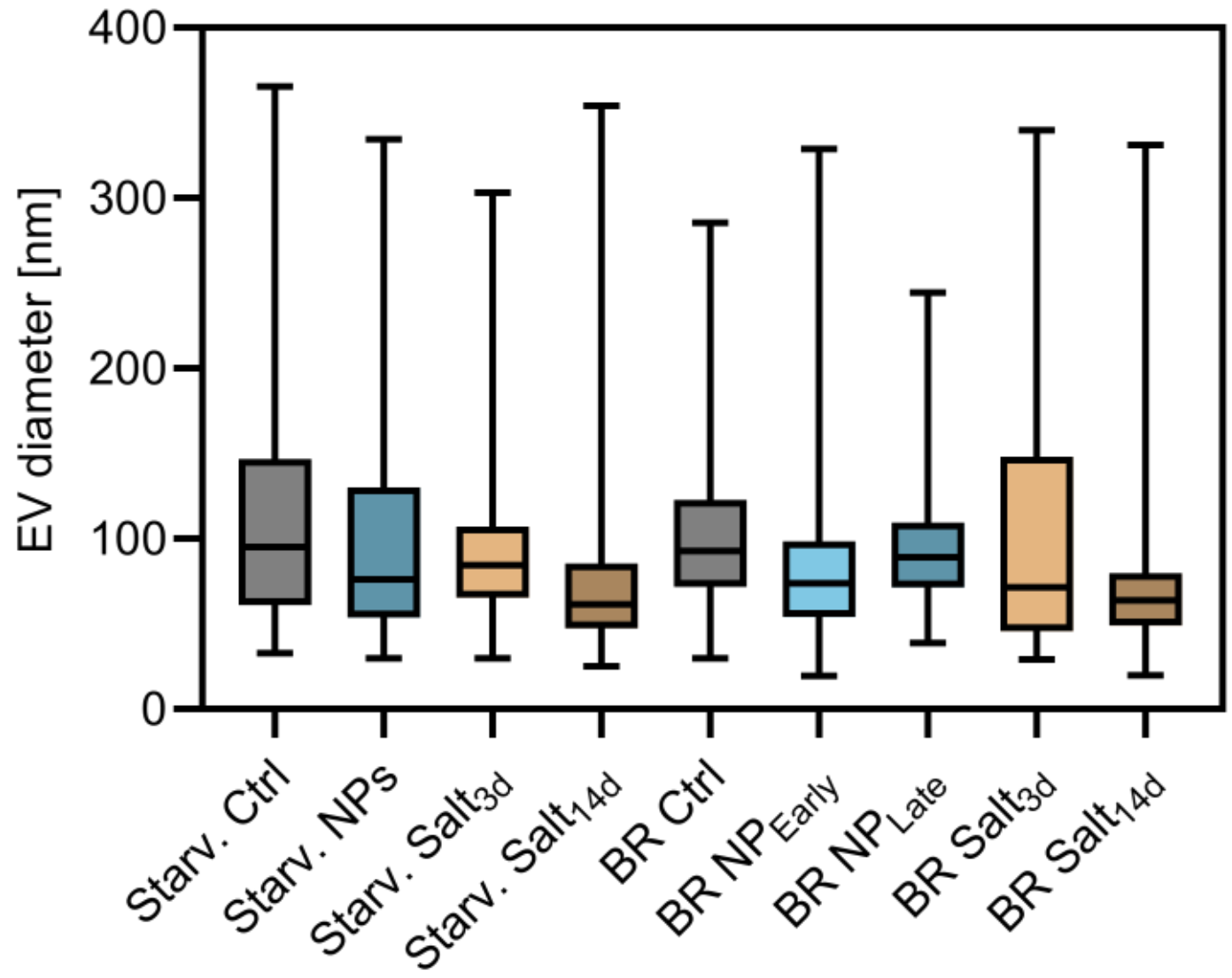

Figure S27. Diameter distribution of EVs, as determined by NTA, produced from mMSCs after 3 hours of turbulence stimulation or 72 hours of serum starvation. The conditions include starvation-mediated production from control cells (Starv. Ctrl), cells incubated with 2 mM IONPs for 30 minutes (Starv. NPs), and cells incubated with 1 mM ferric quinate for 3 days (Starv. $Salt_{3d}$) or 14 days (Starv. $Salt_{14d}$), along with turbulence-mediated production from control cells (BR Ctrl), cells incubated with 2 mM iron oxide nanoparticles (IONPs) for 30 minutes without (BR NP_{Early}) or with a 2-hour chase (BR NP_{Late}), and cells incubated with 1 mM ferric quinate for 3 days (BR $Salt_{3d}$) or 14 days (BR $Salt_{14d}$).

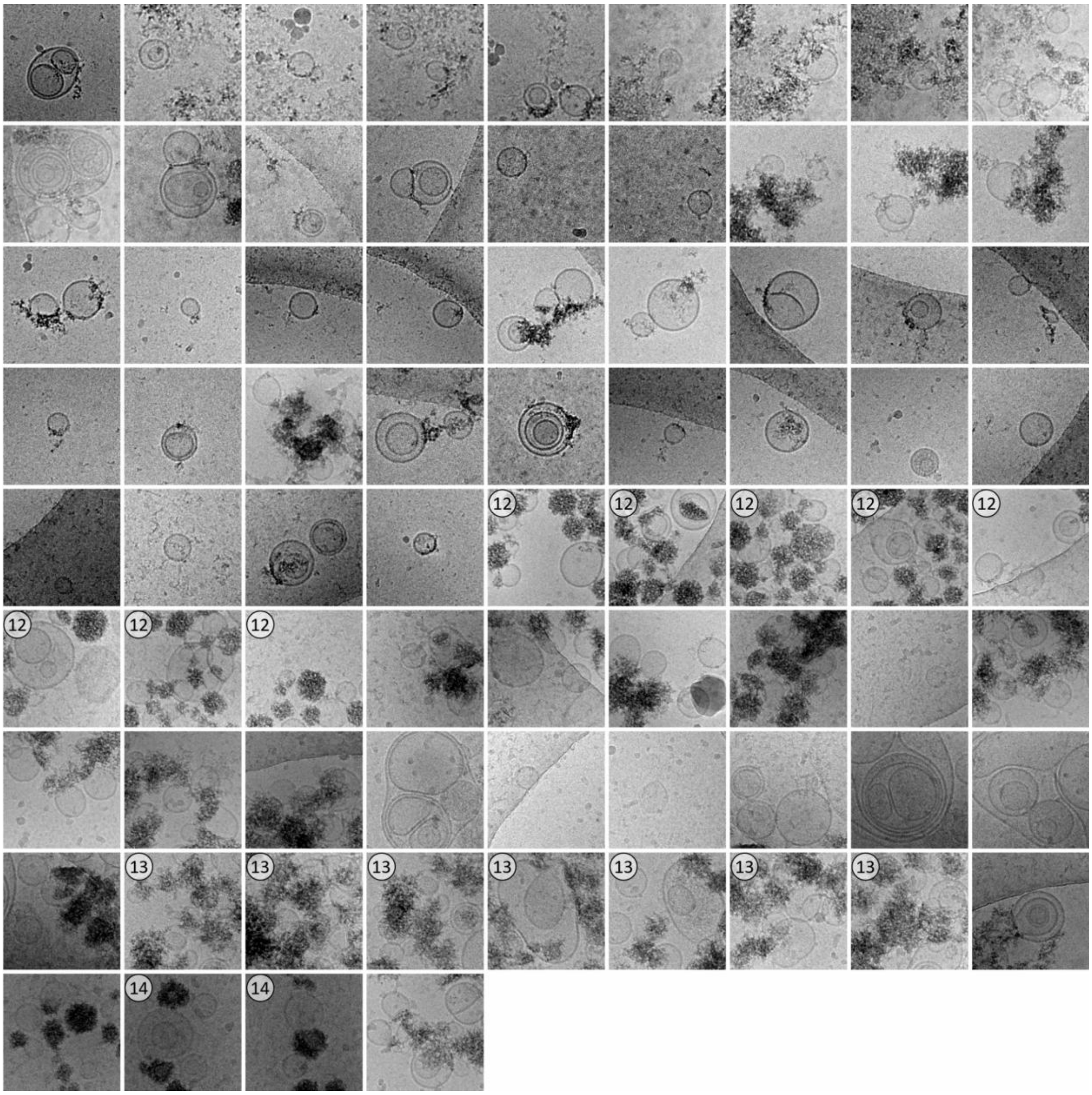

Figure S28. Cryogenic transmission electron microscopy (cryoTEM) of unpurified extracellular vesicles (EVs) produced through turbulence stimulation from early-stage IONP-treated cells. Each image has a scale of 300 nm per side. Circled numbers correspond to the respective X-ray energy dispersive spectroscopy (XEDS) spectrum for the indicated areas.

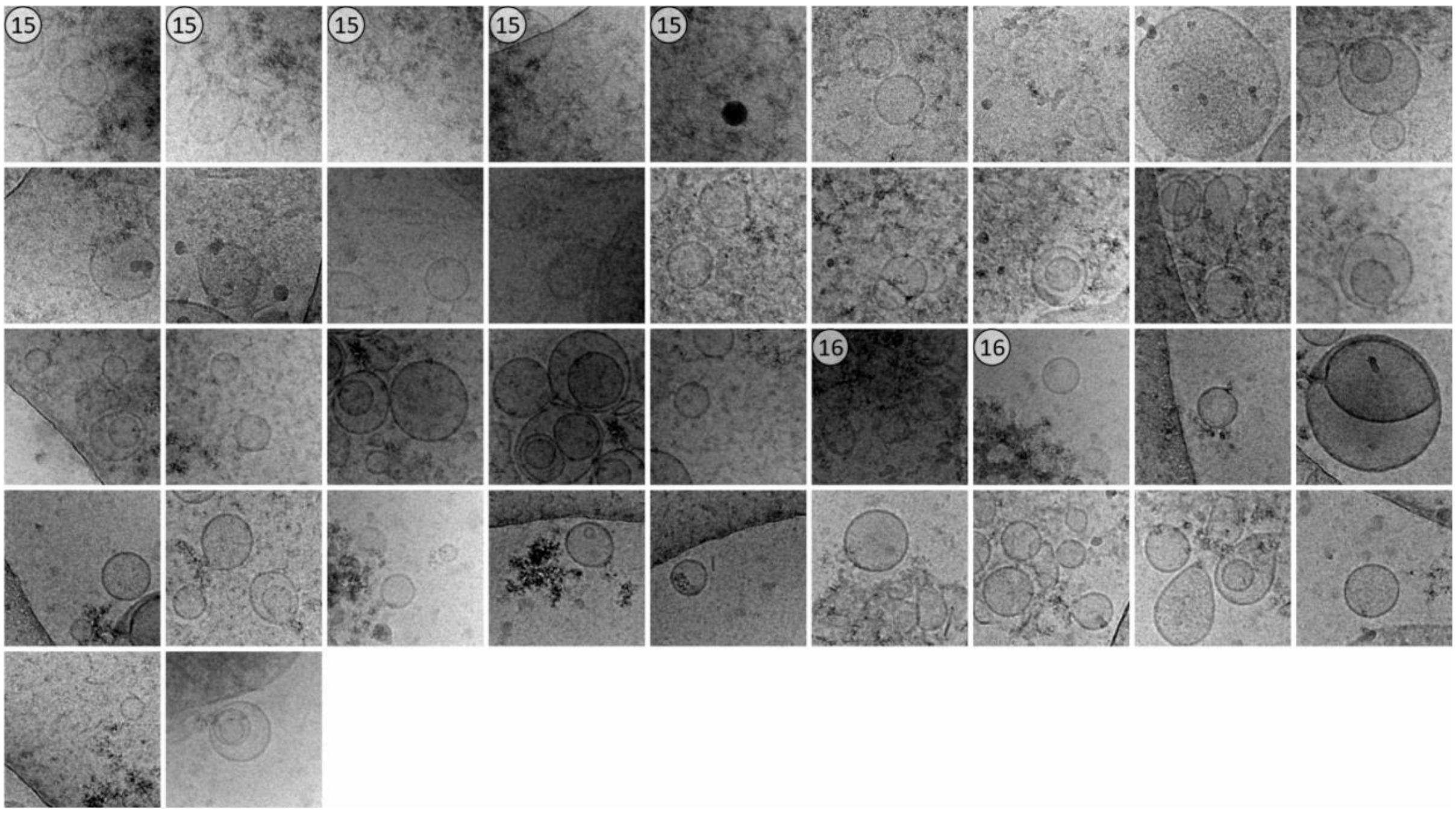

Figure S29. Cryogenic transmission electron microscopy (cryoTEM) of unpurified extracellular vesicles (EVs) produced through turbulence stimulation from late-stage IONP-treated cells. Each image has a scale of 300 nm per side. Circled numbers correspond to the respective X-ray energy dispersive spectroscopy (XEDS) spectrum for the indicated areas.

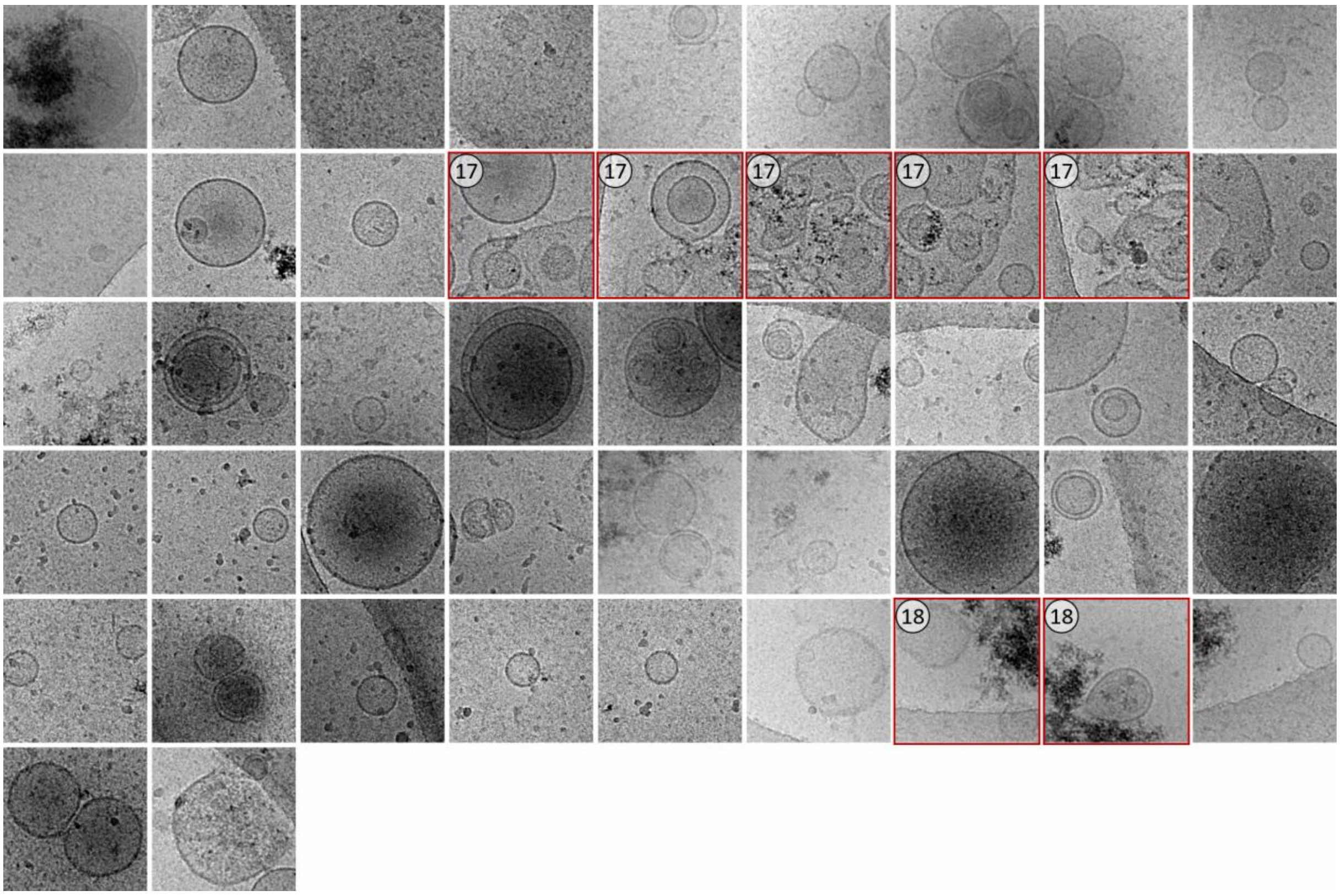

Figure S30. Cryogenic transmission electron microscopy (cryoTEM) of unpurified extracellular vesicles (EVs) produced through turbulence stimulation from short-term ferric quinate-treated cells. Each image has a scale of 300 nm per side. Circled numbers correspond to the respective X-ray energy dispersive spectroscopy (XEDS) spectrum for the indicated areas. Images exhibiting iron material are framed in red.

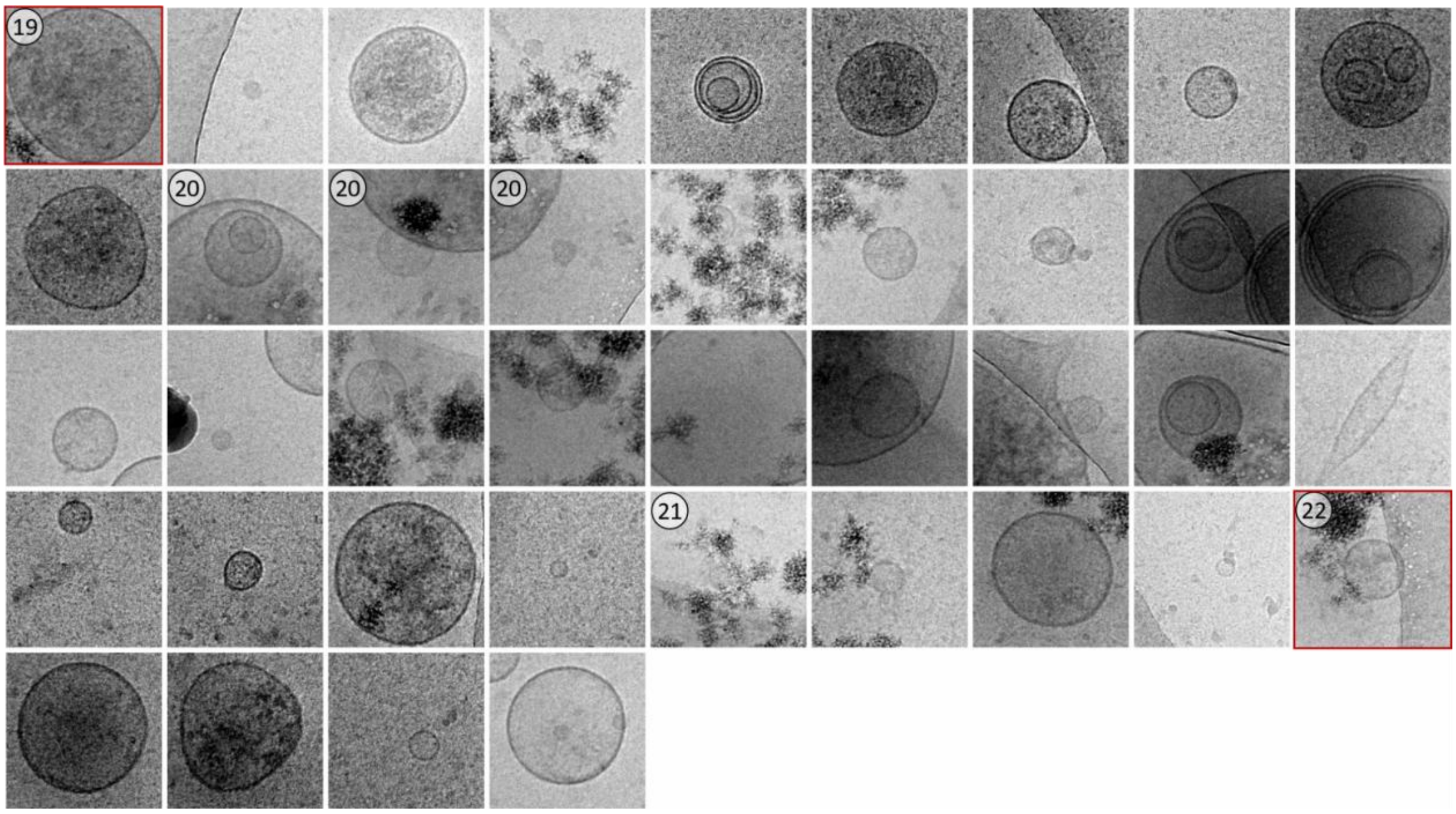

Figure S31. Cryogenic transmission electron microscopy (cryoTEM) of unpurified extracellular vesicles (EVs) produced through serum starvation from IONP-treated cells. Each image has a scale of 300 nm per side. Circled numbers correspond to the respective X-ray energy dispersive spectroscopy (XEDS) spectrum for the indicated areas. Images exhibiting iron material are framed in red.

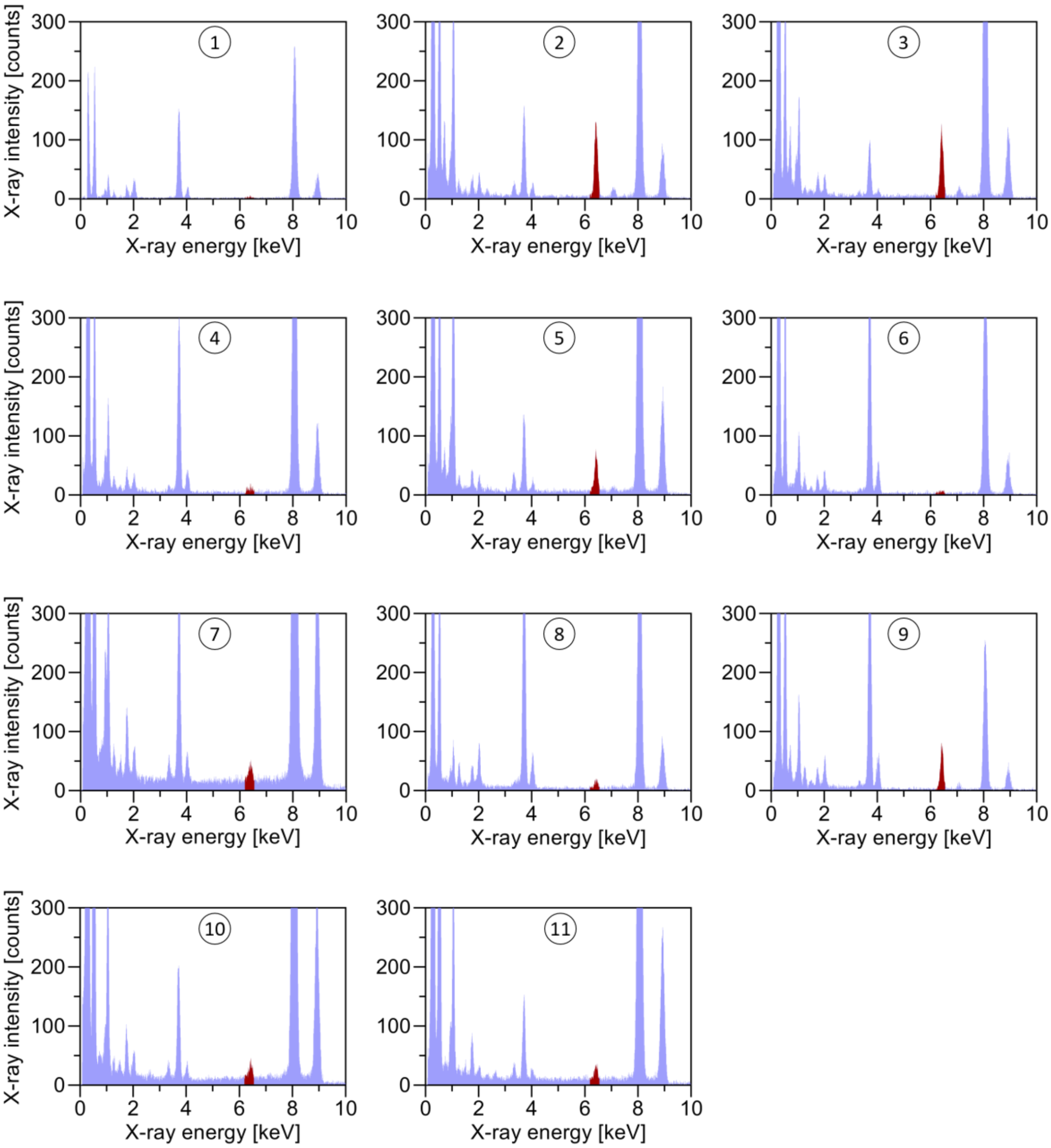

Figure S32. X-ray energy dispersive spectroscopy (XEDS) spectra of EV samples. The red portion of each spectrum highlights the Kα band of iron. Numbers correspond to the associated cryoTEM images.

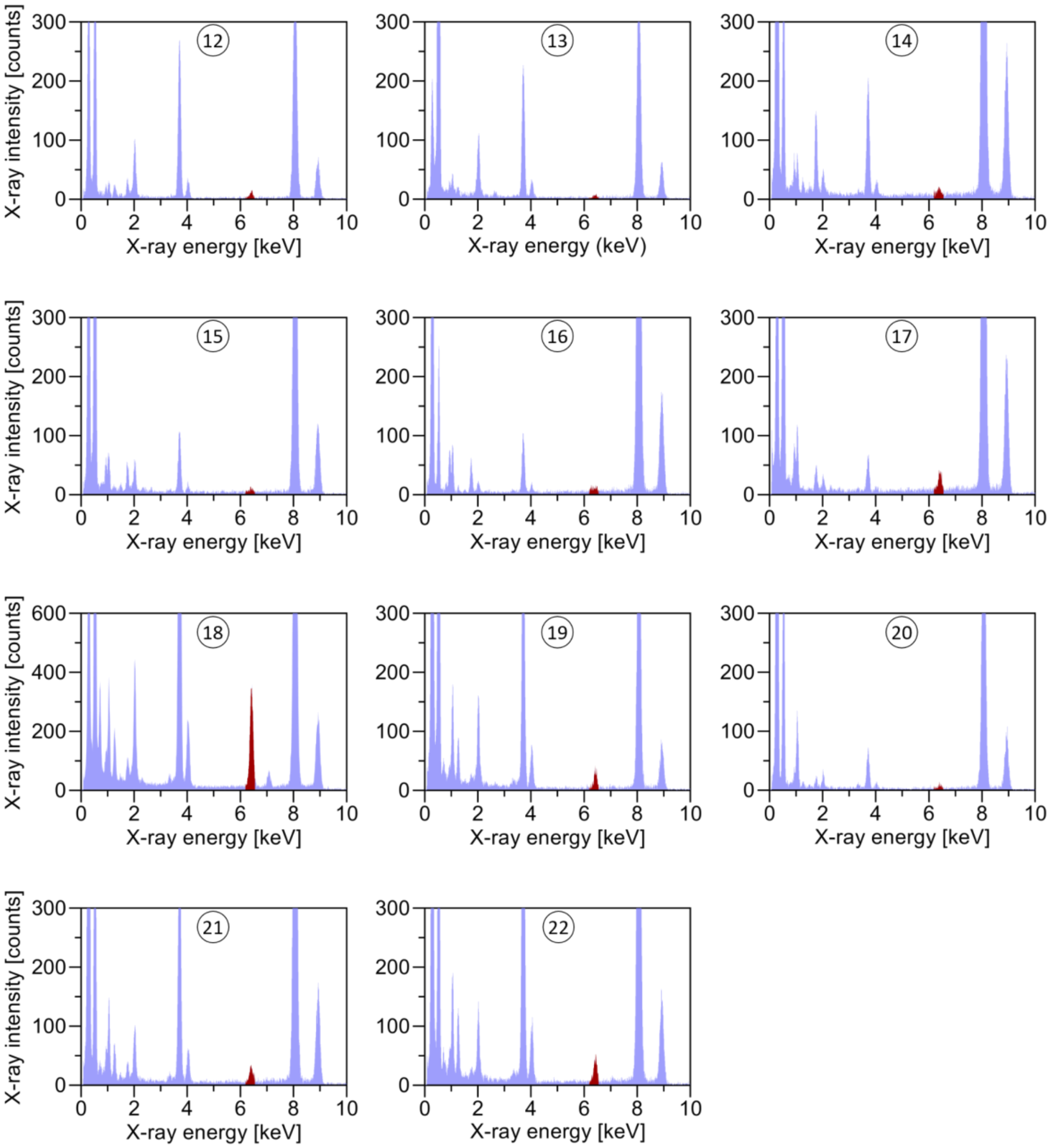

Figure S33. X-ray energy dispersive spectroscopy (XEDS) spectra of unpurified EV samples. The red portion of each spectrum highlights the Kα band of iron. Numbers correspond to the associated cryoTEM images.

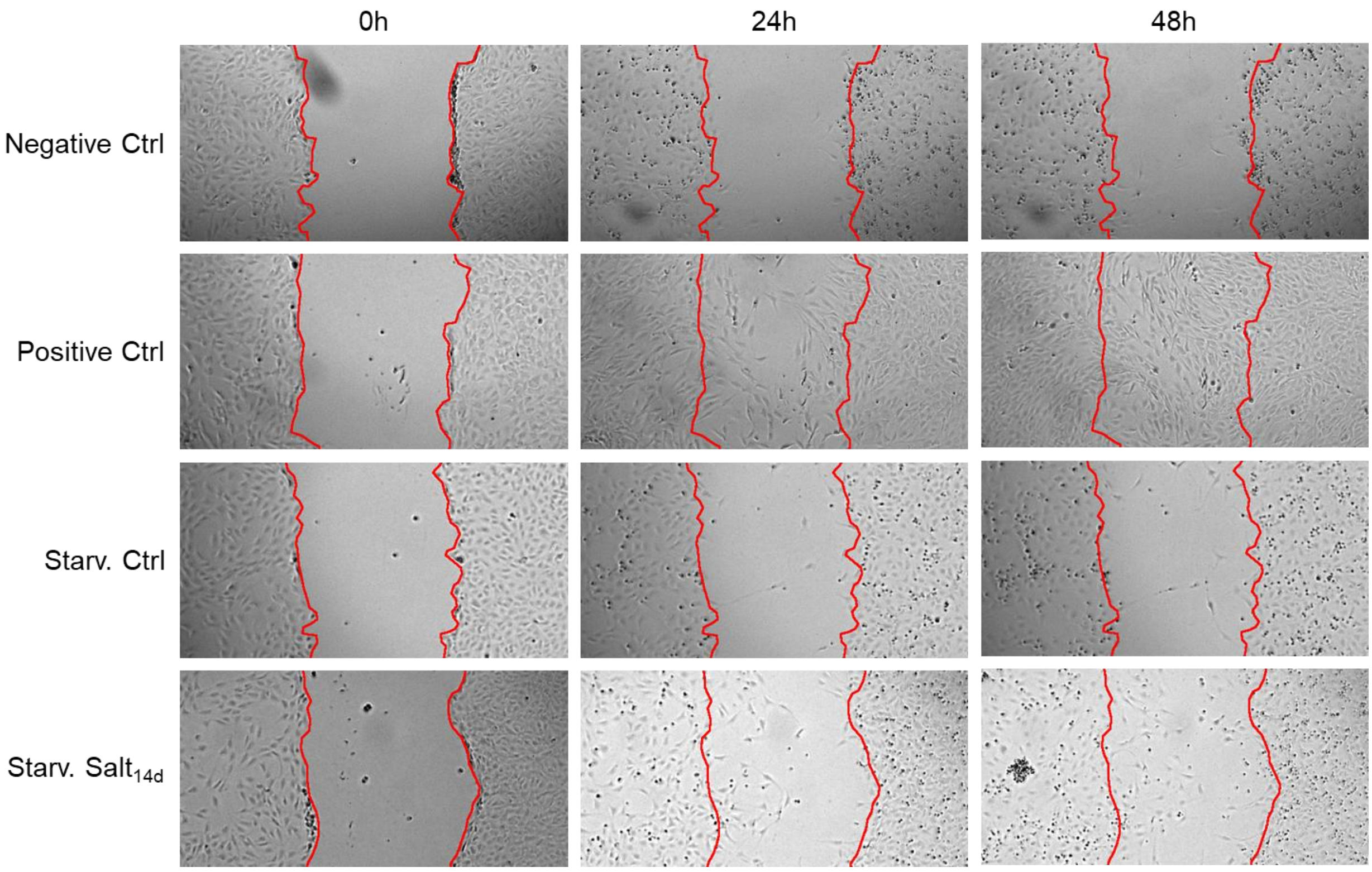

Figure S34. Bright field images of wound closure in murine fibroblasts after 0, 24 and 48 hours of incubation with serum-free medium (Negative Ctrl), medium with 10% FBS (Positive Ctrl), medium supplemented with EVs produced under serum starvation from untreated mMSCs (Starv. Ctrl, $2x10^8$ EVs/mL), and medium supplemented with EVs produced under serum starvation from mMSCs incubated with 1 mM ferric salt for 14 days (Starv. Salt14d, $2x10^8$ EVs/mL).
